# A high-quality assembly reveals genomic characteristics, phylogenetic status, and causal genes for leucism plumage of Indian peafowl

**DOI:** 10.1093/gigascience/giac018

**Published:** 2022-04-06

**Authors:** Shaojuan Liu, Hao Chen, Jing Ouyang, Min Huang, Hui Zhang, Sumei Zheng, Suwang Xi, Hongbo Tang, Yuren Gao, Yanpeng Xiong, Di Cheng, Kaifeng Chen, Bingbing Liu, Wanbo Li, Jun Ren, Xueming Yan, Huirong Mao

**Affiliations:** College of Animal Science, South China Agricultural University, Guangzhou 510642, China; College of Life Science, Jiangxi Science & Technology Normal University, Nanchang 330013, China; College of Life Science, Jiangxi Science & Technology Normal University, Nanchang 330013, China; College of Animal Science, South China Agricultural University, Guangzhou 510642, China; College of Animal Science, South China Agricultural University, Guangzhou 510642, China; College of Animal Science, South China Agricultural University, Guangzhou 510642, China; College of Animal Science and Technology, Jiangxi Agricultural University, Nanchang 330045, China; College of Life Science, Jiangxi Science & Technology Normal University, Nanchang 330013, China; College of Life Science, Jiangxi Science & Technology Normal University, Nanchang 330013, China; College of Life Science, Jiangxi Science & Technology Normal University, Nanchang 330013, China; College of Animal Science and Technology, Jiangxi Agricultural University, Nanchang 330045, China; College of Animal Science and Technology, Jiangxi Agricultural University, Nanchang 330045, China; College of Animal Science, South China Agricultural University, Guangzhou 510642, China; Key Laboratory of Healthy Mariculture for the East China Sea, Ministry of Agriculture and Rural Affairs, Jimei University, Xiamen 361021, China; College of Animal Science, South China Agricultural University, Guangzhou 510642, China; College of Life Science, Jiangxi Science & Technology Normal University, Nanchang 330013, China; College of Animal Science and Technology, Jiangxi Agricultural University, Nanchang 330045, China

**Keywords:** Indian peafowl, genome assembly, phylogeny, PMEL, leucism plumage

## Abstract

**Background:**

The dazzling phenotypic characteristics of male Indian peafowl (*Pavo cristatus*) are attractive both to the female of the species and to humans. However, little is known about the evolution of the phenotype and phylogeny of these birds at the whole-genome level. So far, there are no reports regarding the genetic mechanism of the formation of leucism plumage in this variant of Indian peafowl.

**Results:**

A draft genome of Indian peafowl was assembled, with a genome size of 1.05 Gb (the sequencing depth is 362×), and contig and scaffold N50 were up to 6.2 and 11.4 Mb, respectively. Compared with other birds, Indian peafowl showed changes in terms of metabolism, immunity, and skeletal and feather development, which provided a novel insight into the phenotypic evolution of peafowl, such as the large body size and feather morphologies. Moreover, we determined that the phylogeny of Indian peafowl was more closely linked to turkey than chicken. Specifically, we first identified that *PMEL* was a potential causal gene leading to the formation of the leucism plumage variant in Indian peafowl.

**Conclusions:**

This study provides an Indian peafowl genome of high quality, as well as a novel understanding of phenotypic evolution and phylogeny of Indian peafowl. These results provide a valuable reference for the study of avian genome evolution. Furthermore, the discovery of the genetic mechanism for the development of leucism plumage is both a breakthrough in the exploration of peafowl plumage and also offers clues and directions for further investigations of the avian plumage coloration and artificial breeding in peafowl.

## Introduction

**Figure 1:**
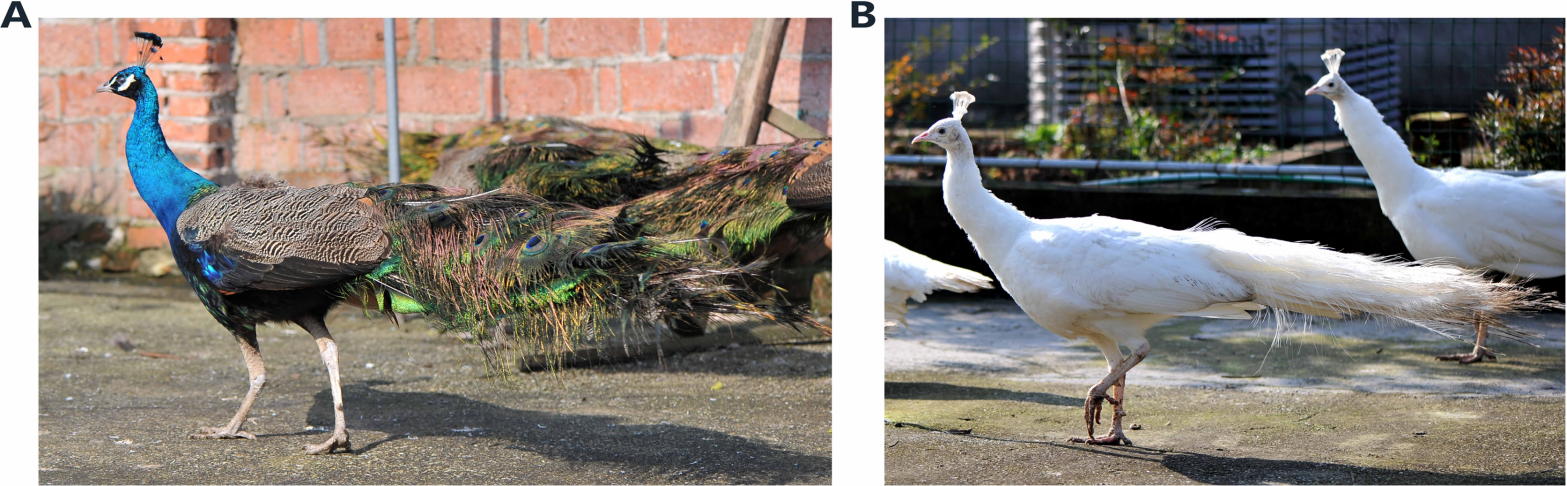
Photographs of (**A**) the Indian blue peafowl and (**B**) leucistic peafowl.

*Pavo cristatus* (NCBI:txid9049), commonly called the Indian peafowl or blue peafowl, represents elegance, honour, beauty, luck, and romance in many Asian cultures (Fig. 1a) [1, 2]. Peafowl belongs to Aves, Galliformes, Phasianidae, *Pavo*, and has 2 species: green peafowl and blue peafowl. The Indian peafowl is the national bird of India and is widely distributed in Bangladesh, Bhutan, India, Nepal, Pakistan, and Sri Lanka [2, 3]. Indian peafowl has exclusive characteristics, even in the Phasianidae family; e.g., it has a larger body size, fan-shaped crests, glittering plumage, and an iridescent tail, and is of great ornamental value. These qualities have attracted scientific and research attention. Moreover, many studies have suggested that Indian peafowl is a protein resource with high nutritional value, including its meat, internal organs, and bones; furthermore, it has medicinal value and is therefore widely bred in many countries [4–6].

With the improvement of whole-genome sequencing technology, an increasing number of avian genomes are being assembled, such as *Numida meleagris* [7], *Phasianus colchicus* [8], and *Fringilla coelebs* [9], which provide basic references for the study of phenotypic characteristics, evolution, economic traits, and environmental adaptation of birds [10]. Comparative genomics analysis is an important tool for revealing the adaptive evolution, phenotypic evolution, and genome characteristics of species [11], and it is widely applied to studies of the evolution and origin of animals or plants [12–14]. The first draft of Indian peafowl genome assembly was released in 2018. However, the length of scaffold and contig N50 of the assembly were only 25.6 and 19.3 kb, respectively [15]. Subsequently, Dhar et al. improved the Indian peafowl genome using Illumina and Oxford Nanopore technology, and the length of scaffold N50 was determined up to 0.23 Mb [16]; however, the assembly quality still needed improvement. Additionally, previous studies of Indian peafowl were mainly focused on courtship behaviour [17], immunity [18], and productivity [19, 20]. Some studies regarding the phylogeny of Indian peafowl have been based on the mitochondrial genome, DNA transposable factors, and partial DNA nucleotide sequences, but few reports have addressed the whole-genome level [20–22]. Therefore, an improved genome of the Indian peafowl is needed to provide baseline data for further studies on this species, including genomic characteristics, and adaptive and phenotypic evolution.

Avian plumage is colourful and attractive; it has functions in protection, courtship, and signal identification and provides an excellent model for the exploration of plumage formation, behaviour, and phenotypic evolution in animals. Interestingly, studies on Indian peafowl plumage colour report that there are many plumage colour mutants, including white, black, variegated, cameo, and oaten [23–25], among which, the most ornamental colour is the white plumage, caused by leucism rather than albinism, so the feathers are white but the eyes contain melanin pigmentation (Fig. 1b). The inherited basis of plumage colour has attracted researchers for a long time. The first reports suggested that the plumage phenotype of peafowl was determined by autosomal genes in a recessive model [24]. A later study verified that a single autosomal locus was in control of all plumage phenotypes in peafowl, where the pied colour appeared in 2 heterozygous mutant alleles, with black on the recessive mutant allele and the all leucism plumage on the homozygous mutant allele as the most dominant [23]. Nevertheless, further studies on the genetic mechanism of the leucism plumage in peafowl were needed to clarify the causative mutations of this phenotype.

A high-quality (near-chromosomal) reference genome of the Indian peafowl was constructed using third-generation *de novo* assembly technology. Based on the assembly, comparative genomics analysis was performed to investigate the biological characteristics of evolution at the genome-wide level through comparing the Indian peafowl genome with the high-quality genomes of other birds, humans, and the mouse. Furthermore, transcriptomic and pooled resequencing data were analysed to identify the genetic mechanism of the leucism plumage variant in Indian peafowl. This work will provide an updated understanding and key reference for genomic characteristics, adaptive and phenotypic evolution, and the genetic mechanism of the leucism plumage trait in Indian peafowl.

## Materials and Methods

### Sample collection

All procedures used for this study that involved animals fully complied with guidelines for the care and use of experimental animals established by the Ministry of Agriculture of China. The ethics committee of South China Agricultural University approved this study. A blood sample was collected from a female Indian peafowl for genome assembly and 51 blood samples from 35 blue feather peafowls and 16 leucistic plumage peafowls for pooled resequencing in Leping Sentai special breeding Co., Ltd, in Jiangxi Province, China, under the principles and standards of animal welfare ethics. Meanwhile, 2 liver and 2 muscle tissue samples were obtained from the female Indian peafowl to assist the process of genome assembly. Additionally, feather pulp samples from 4 blue and 4 leucistic peafowls were collected for RNA sequencing (RNA-seq).

### DNA and RNA extraction

Genomic DNA was extracted from blood samples using a routine phenol-chloroform protocol. The concentration of the extracted DNA was evaluated using a Nanodrop 2000 spectrophotometer (Thermo Fisher Scientific, Waltham, MA, USA), and diluted to a final concentration of 100 ng/μL. The integrity of DNA was checked via electrophoresis on 0.8% agarose gel. Total RNA of feather pulp was extracted using TRIzol reagent (Thermo Fisher Scientific, Waltham, MA, USA). The purity and degradation of RNA was detected by Nanodrop 2000 spectrophotometer and agarose gel electrophoresis.

### *De novo* assembly of the Indian peafowl reference genome

#### Library preparation and sequencing

Genomic DNA was used to make 350-bp insert fragment libraries using the Illumina TruSeq Nano method, starting with 100 ng DNA. Mate pair libraries were made by Nextera Mate Pair Sample Preparation Kit (Illumina) with the gel plus option, and sequenced using Illumina NovaSeq 6000 platform (Illumina NovaSeq 6000 Sequencing System, RRID:SCR_016387). For Pacific Biosciences (PacBio) sequencing, genomic DNA was sheared by a g-TUBE device (Covaris) with 20 kb settings for further preparing a 20 kb Single-Molecule Real Time (SMRT) bell, and then the single-molecule sequencing was completed on a PacBio RS-II platform (PacBio Sequel II System, RRID:SCR_017990). For 10× genomics sequencing, each GEM was amplified by PCR and added P7 sequencing adapters for Illumina sequencing.

#### Genome assembly

The genome assembly of Indian peafowl was performed in 5 steps, which are illustrated in Supplementary Fig. S1. The raw reads were generated from 2 paired-end libraries sequenced on Illumina NovaSeq 6000 platform. The sequencing adapters, contaminated reads, and low-quality reads were removed using megablast v2.2.26 [26]. The genome size was calculated by the following formula: Genome size = kmer_Number/Peak_Depth. Second, PacBio sequencing was used to control and correct errors. The error-corrected data were assembled by falcon (Falcon, RRID:SCR_016089) software [27], and the Overlap-Layout-Consensus algorithm was used to obtain the consensus sequences, which were then corrected by quiver software [28]. Combined with the second-generation sequencing data, the consensus sequences were recalibrated using the pilon (Pilon, RRID:SCR_014731) software [29] to improve the accuracy, and high-quality consensus sequences were obtained. Third, 10× Genomics sequencing was used to assist the genome assembly. The 10× Genomics library was sequenced to obtain linked reads, which were aligned to the consensus sequences obtained from the PacBio sequencing assembly, and then linked reads were added to assemble the super-scaffolds by fragScaff software [30]. Fourth, similar to the third step, Chicago sequencing data were used to assist the mapping of the draft genome assembly. Finally, the Illumina reads were mapped to the draft genome using BWA (BWA, RRID:SCR_010910), [31]. Then, pilon (version 1.22) was used to correct the assembled errors based on the mapped results.

#### Consistency and completeness

The consistency and integrity of the assembled peafowl genome were separately assessed using BUSCO (BUSCO, RRID:SCR_015008) [32] and CEGMA (CEGMA, RRID:SCR_015055) [33, 34], based on single-copy orthologues from the AVES (odb9) database. To evaluate the accuracy, integrity, and sequencing uniformity of the genome assembly, small fragment library reads were selected and aligned to the assembled genome using BWA software. All the genomic sequences were generated by Novogene Inc., Beijing, China.

#### Genome annotation

Genome annotation mainly included 3 aspects: repetitive sequence annotation, gene annotation (including gene structure prediction and gene function prediction), and non-coding RNA annotation (Supplementary Fig. S2). The repetitive sequence annotation included the annotation through homologous sequence alignment and *ab initio* prediction. The RepeatMasker (RepeatMasker, RRID:SCR_012954) and RepeatproteinMask software [35] were used to identify known repetitive sequences against the RepBase (Repbase, RRID:SCR_021169) library [36]. In *ab initio* prediction, LTR_FINDER [37], RepeatScout (RepeatScout, RRID:SCR_014653) [38], and RepeatModeler (RepeatModeler, RRID:SCR_015027) [39] were used to establish the *de novo* repeat sequence library, and then repetitive sequences were predicted by Repeatmasker software. The tandem repeats (TEs) in the genome were found by Tandem Repeat Finder software [40]. In gene annotation, it mainly combined 3 prediction methods: homology-based prediction, *de novo* prediction, and other evidence-backed predictions. Homology-based prediction used the protein sequences of chicken, turkey, common mallard, African ostrich, crested ibis, and Eastern Zhejiang white goose, downloaded from Ensembl (Ensembl, RRID:SCR_002344) (release 74), to align to the Indian peafowl genome using TblastN (TBLASTN, RRID:SCR_011822) [41]. Genewise (GeneWise, RRID:SCR_015054) [42] was used to align to the matched proteins for a precise gene model.

In addition, Augustus (Augustus, RRID:SCR_008417) [43], GlimmerHMM (GlimmerHMM, RRID:SCR_002654) [44], Geneid [45], GenScan  (GENSCAN, RRID:SCR_013362) [46], and SNAP software  (SNAP, RRID:SCR_002127) [47] were used for the *ab initio*predictions of gene structures. The above predictions with transcriptome-based data being combined, EVidenceModeler software (EVidenceModeler, RRID:SCR_014659) [48] was used to integrate the gene set and generate a non-redundant and more complete gene set. Finally, PASA was used to correct the annotation results of EVidenceModeler for the final gene set. Gene function of the final gene set was annotated using the protein database of SwissProt [49], NR [50], Pfam (Pfam, RRID:SCR_004726), [51], KEGG (KEGG, RRID:SCR_012773) [52], and InterPro (InterPro, RRID:SCR_006695) [53]. tRNAscan-SE software [54] was used to search for the transfer RNA (tRNA) sequence of genome, with INFERNAL software (Infernal, RRID:SCR_011809) [55] from Rfam (Rfam, RRID:SCR_007891) [56] to predict microRNAs and snRNA of genome.

### Gene family

The amino acid sequences of the following were downloaded from NCBI database to identify the gene families and single-copy orthologous genes. They are: Japanese quail (*Coturnix japonica*) [57], chicken (*Gallus gallus*) [58], turkey (*Meleagris gallopavo*) [59], northern bobwhite (*Colinus virginianus*) [60], common mallard (*Anas platyrhynchos*) [61], zebra finch (*Taeniopygia guttata*) [62], collared flycatcher (*Ficedula albicollis*) [63], medium ground-finch (*Geospiza fortis*) [12], Tibetan ground-tit (*Pseudopodoces humilis*) [64], rock pigeon (*Columba livia*) [65], peregrine falcon (*Falco peregrinus*) [66], saker falcon (*Falco cherrug*) [67], human (*Homo sapiens*) [68], and mouse (*Mus musculus*) [69]. The longest transcript of each gene was extracted and then the genes with the length of protein sequences shorter than 50 amino acids were filtered. Based on the filtered protein-coding sequences data set, Orthofinder  (OrthoFinder, RRID:SCR_017118) v2.3.7 [70] was used to identify gene families and orthologous gene clusters of 15 species. The single-copy orthologous sequences from the gene families were aligned using MAFFT (MAFFT, RRID:SCR_011811) v7.450 software [71], and then the poorly sequences were removed using Trimal software (trimAl, RRID:SCR_017334) with default parameters [72]. The final result was used as a single data set for the subsequent comparative genome analyses.

### Phylogenetic tree and divergence time

To determine the phylogenetic relationship of 15 species, IQ-tree (IQ-TREE, RRID:SCR_017254) v2.1.2 software was first used to find the best model for constructing phylogenetic tree with options “-m MF” and the species tree with bootstrap 1000 based on the concatenated alignment of single-copy orthologues sequences from 15 species [73]. RAxML (RAxML, RRID:SCR_006086) software was used to construct phylogenetic tree with parameters “-m PROTGAMMALGX -f a” with bootstrap 1000. Divergence time of 15 species was estimated by using MCMCtree program implemented in PAML packages (PAML, RRID:SCR_014932), [74]. Five calibration time (human-mouse (85–97 Mya), human-zebra finch (294∼323Mya), zebra finch-medium ground finch (30.4∼46.8Mya), common mallard-zebra finch (93.2∼104.6Mya) and saker falcon-peregrine falcon (1.66∼3.68Mya)) from TimeTree (TimeTree, RRID:SCR_021162) database [75] were used as constrains in the divergence time estimation. The MCMC process was run to sample 1,000,000 times, sample frequency set to 10, and burn-in 40 000, to finally achieve a convergence until the value of efficient sampling size (ESS) >200 using Tracer (Tracer, RRID:SCR_019121) v1.7.1.

### Genome synteny and collinearity analysis

To compare the genome synteny of peafowl with chicken and turkey, the homologue of the genome was identified using BLASTp (BLASTP, RRID:SCR_001010) (E-value < 1e^–10^). Gene pairs of synteny blocks within the genome were identified using MCScanX [76], and the synteny blocks were showed by circos program from TBtools [77]. To estimate the positively selected genes for peafowl-chicken and peafowl-turkey, the value of Ka/Ks (ω) for each gene pair was calculated by KaKs_calculator [78], and the density curve of values was visualized by R software. The positively selected genes (ω > 1) were conducted based on functional enrichment analysis.

### Gene-family expansion and contraction

To identify the gene family expansion and contraction in peafowl, the gene families in 15 species and phylogenetic tree with divergent times were taken into account to estimate the significance of gene gain and loss in gene family using the CAFE (CAFE, RRID:SCR_005983) v4.2.1 with a random birth and death model and significance of P-values < 0.05 [79]. The parameter λ represents the probability of gene gain and loss in a divergent time. In order to investigate the evolutionary rates of different branches of the tree, the argument with “-t” was used to define 3 different branches for 15 species: the first branch included mouse and human, the second branch was the Phasianidae, and other birds were regarded as the third branch. Then, they were conjunct with the “-s” option to search the optimal λ-value for different branches using maximum likelihood.

### Positive selection analyses

To determine adaptive evolution under positive selection in peafowl, the single-copy orthologous protein sequences shared among the 11 species (peafowl, chicken, turkey, common mallard, zebra finch, collared flycatcher, medium ground-finch, Tibetan ground-tit, rock pigeon, peregrine falcon, and saker falcon) were searched, filtered, and then converted to coding gene sequence (CDS) using the EMBOSS backtranseq program [80]. The CDS were aligned to codon by using the PRANK (prank, RRID:SCR_017228) with the option “-codon” [81]. The above alignments were analysed by CODEML program of the PAML package 4.9 [74]. A branch-site model (TEST-II) (model = 2, NSsites = 2) was conducted to identify the positively selected genes of peafowl. The model assumed that a particular branch (foreground, alternative hypothesis) had a different ω-value from all the sites compared to all other branches (background, null hypothesis), suggesting that positive selection occurred at only a few sites on a particular branch (foreground) [74]. The peafowl was regarded as a foreground branch and other species as a background branch. Additionally, the branch model was used to identify the rapidly evolving genes in peafowl, assuming that the branch of peafowl was an alternative hypothesis (model = 2) and the branches of other species were the null hypothesis (model = 0). The *dN*/*dS* (ω) values between foreground branch and background branch were estimated using likelihood ratio test values based on the Χ^2^ test. When the ω-value in the foreground branch was greater than in the background branch, it suggested that the genes of the foreground branch were under positive selection (*P* < 0.05) and the positively selected sites were determined using the Bayesian empirical Bayes (BEB) method. All the positively selected genes underwent functional enrichment analysis using KOBAS (KOBAS, RRID:SCR_006350) [82].

### Whole-genome resequencing and variant calling

The genomic DNA from 35 blue feather peafowls and 16 leucistic plumage peafowls were pooled, respectively. Then 1.5 μg DNA per pool was used for constructing the sequencing libraries using Truseq Nano DNA HT Sample preparation Kit (Illumina, USA) following manufacturer's constructions. Each pooled DNA sample was fragmented through sonication to a size of 350bp and end repaired, A-tailed, and ligated with the full-length adapter for Illumina sequencing with further PCR amplification. PCR-amplified sequencing libraries were purified (AMPure XP system) and analysed for size distribution on Agilent2100 Bioanalyzer (Agilent 2100 Bioanalyzer Instrument, RRID:SCR_019389) and were quantified using real-time PCR. These libraries constructed above were sequenced on an Illumina NovaSeq platform, and 150-bp paired-end reads were generated with insert size ∼350 bp. The raw data were filtered by removing reads with ≥10% unidentified nucleotides (N), reads with >50% bases having phred quality <5, and reads with >10 nt aligned to the adapter allowing ≤10% mismatches. The clean reads were mapped to the assembled reference genome using BWA with parameters “mem -t 4 -k 32 –M –R.” Alignment files were converted to BAM files using SAMtools (SAMTOOLS, RRID:SCR_002105) software (settings: –bS –t) [83]. In addition, potential PCR duplications were removed using SAMtools command “rmdup.” Single-nucleotide polymorphisms (SNPs) and insertions/deletions (Indels) (<50 bp) were detected using GATK (GATK, RRID:SCR_001876) v 4.0 pipeline [84].

### RNA sequencing on PacBio platform

The complementary DNA (cDNA) of feather was acquired through PrimeScript™ RT reagent Kit with gDNA Eraser (Takara Bio, Inc., Dalian, China) according to the manufacturer's instructions. The cDNA underwent damage repair, end repair, SMRT dumbbell-shaped adapters, and ligation of the adapters to construct a mixed library. Primers and DNA polymerase were then combined to form a complete SMRT bell library. The qualified library was used for sequencing on a PacBio Sequel platform. The clean data were aligned to the reference genome of Indian peafowl by STAR v2.5.3a [85]. The transcript assembly and gene expression levels were conducted by StringTie (StringTie, RRID:SCR_016323) v1.3.3 [126] and featureCounts (featureCounts, RRID:SCR_012919)[127] in Subread (Subread, RRID:SCR_009803) software [86]. Differentially expressed genes (DEGs) between blue and leucistic plumage were identified through DESeq2 (DESeq2, RRID:SCR_015687)[128] in condition of fold change >2 and *P* < 0.01. Subsequently, the functional enrichment analyses of DEGs were annotated through the GO (Gene Ontology) [87] and KEGG databases.

### cDNA amplification

cDNA of feathers was reversely transcribed with PrimeScript™ RT reagent Kit with gDNA Eraser (Takara). The reverse transcription quantitative PCR (RT-qPCR) was conducted in a total volume of 10 µL including 5 µL SYBR Taq II kit (Takara), 0.3 µL Rox Reference Dye (50×), 2.7 µL distilled water, 1 µL cDNA, and 1 µL primers, and performed on a 7900HT RT-qPCR system (ABI). β-actin was selected as the internal reference gene. All primer sequences are shown in Supplementary Table S20.

## Results

### Genomic characteristics of Indian peafowl

**Figure 2:**
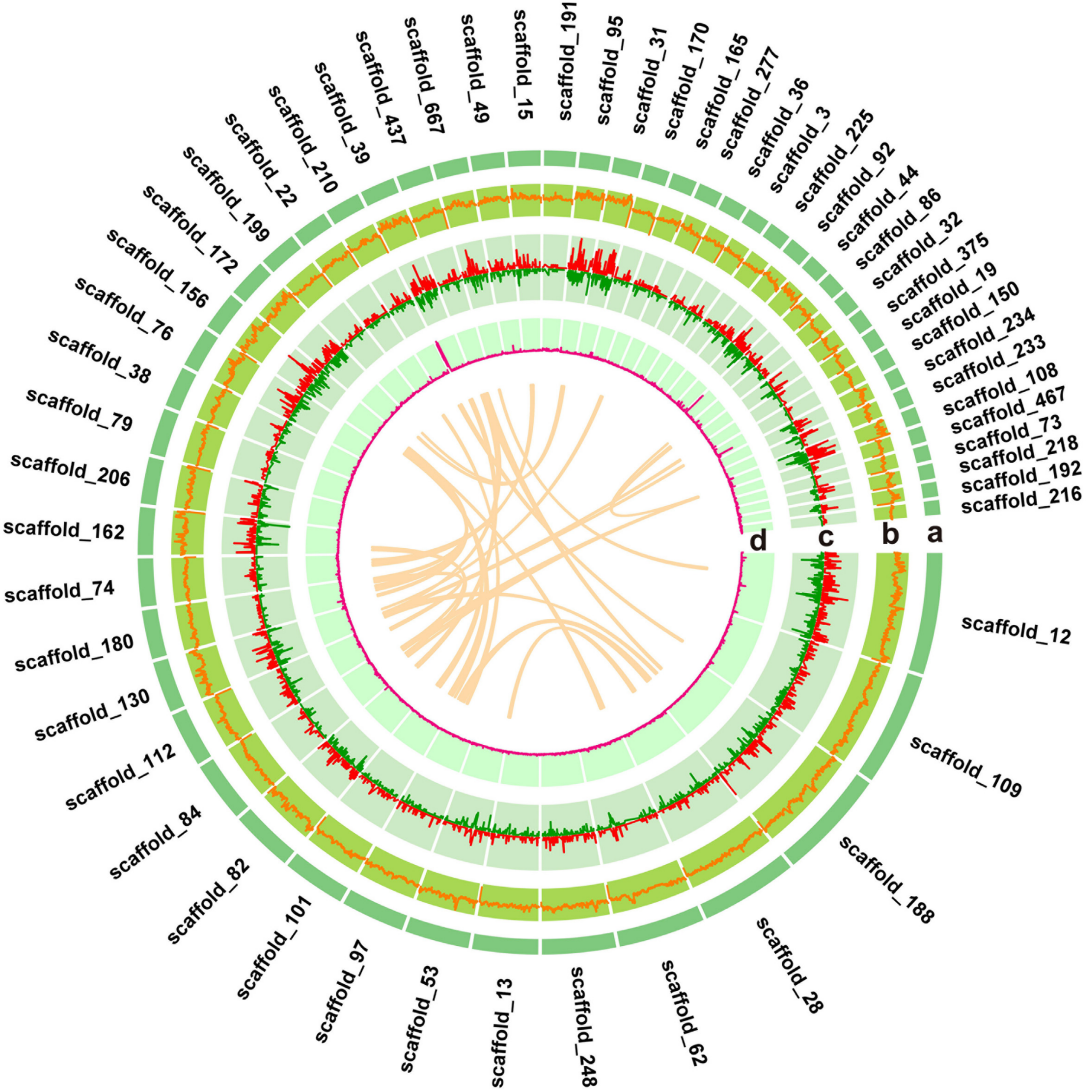
The global maps of *de novo*genome assembly of Indian peafowl. **a**, 55 scaffolds with length >5 Mb (scaffold N70) of the assembled Indian blue peafowl. The perimeter of the ring represents the length of scaffolds, and the light orange links in the middle circle indicate the synteny in the peafowl genome. The GC density, gene density, and tandem repeat sequence density of peafowl genome are displayed in **b, c**, and **d**, respectively. Red and green bars in the gene density diagram represent the positive strand (+) and negative strand (−) in peafowl genome.

Third-generation PacBio SMRT sequencing technology and second-generation Illumina sequencing technology were used and combined with 10X genomics to assemble the Indian peafowl genome. We obtained a sequencing volume of 164.03 Gb using an Illumina NovaSep 6000 platform, 112.57 Gb of sequencing data on a 10X Genomics sequencing platform, and 110.74 Gb of sequencing data using the PacBio sequencing platform (Supplementary Table S1). In total, 387.34 Gb of sequencing data and a total coverage of 362× was obtained from the 3 sequencing strategies with the lengths of contig N50 and scaffold N50 separately up to 6.2 and 11.4 Mb, respectively, which exhibited a 50-fold improvement in the scaffold N50 compared to the previously published Indian blue peafowl genome reported by Jaiswal et al. [15] and Dhar et al. [16] (Fig. 2, Table 1, and Supplementary Table S2). The distribution of 17-mer showed a major peak at 154× (Supplementary Fig. S3). The Indian peafowl genome size was estimated to be 1.05 Gb. The present peafowl assembly was anchored into 726 scaffolds, and guanine-cytosine (GC) content was 42.03% with a normal ratio of A, T, G, and C (Fig. 2 and Supplementary Table S2–S3).

**Table 1:** Quality metrics for the present peafowl genome assembly and for other peafowl genome assemblies published in previous studies

Metric	This study	Shubham et al. (2018)[129]	Ruby et al. (2019)[130]
Sequencing technology	Illumina NovaSeq 6000, PacBio RS-II, 10X Genomics, Chicoga	Illumina NextSeq 500	Illumina HiSeq, ONT
Total sequencing depth	362×	136×	236×
Total scaffolds	726	98,687	179,332
Scaffolds N50 (bp)	11,421,185	25,613	190,304
Contigs N50 (bp)	6,188,159	19,387	103,131
Longest scaffold length (bp)	38,857,732	286,113	2,488,982
Total sequence length (bp)	1,046,718,946	1,137,150,029	1,027,510,962
Total number of predicted protein-coding genes	19,465	15,970	23,153

ONT: Oxford Nanopore technology.

We assessed the completeness and base accuracy of our Indian peafowl genome assembly using CEGMA and BUSCO. Assembly of the draft genome presented a high mapping rate (98.05%) and coverage rate (99.87%) and low homozygous SNP rate (0.0002%) by mapping to the short reads, generally reflecting the high accuracy of genome assembly (Supplementary Tables S4 and S5). The BUSCO results showed that 88.71% of 248 core genes selected from 6 eukaryotic model organisms were covered. Additionally, 97.4% complete genes (including 96.8% complete and single-copy genes and 0.6% complete and duplicated genes) were predicted, and 1.7% fragmented genes and 0.9% missing genes were identified from 2,586 genes in the Aves dataset (Supplementary Table S6). Collectively, these important indicators implied relatively high genome coverages and continuity for the Indian peafowl genome, providing an important resource for molecular breeding and evolutionary studies of peafowl.

According to the homologous alignment and *ab initio*prediction, the Indian peafowl genome comprised 15.20% non-redundant repeat sequences, including 1.27% tandem repeats, 14.12% transposable elements, and 7.35% transposable element proteins (Supplementary Table S7). A total of 14.56% of transposable elements were identified after combined TEs, 0.70% of which were DNA transposons, 3.93% long terminal repeats (LTRs), 0.01% short interspersed nuclear elements, and 10.68% long interspersed nuclear elements (Supplementary Fig. S4 and Supplementary Tables S7 and S8). Altogether, 19,465 non-redundant protein-coding genes were predicted, of which 15,766 (81%) were annotated to function according to 6 public databases (Table 1 and Supplementary Tables S9 and S10). Additionally, 354 microRNAs, 308 tRNAs, 151 ribosomal RNAs, and 334 small nuclear RNAs were also identified (Supplementary Table S11). Overall, this assembly had improved continuity, completeness, and accuracy.

### Gene families and phylogenetic relationship of 15 species

**Figure 3:**
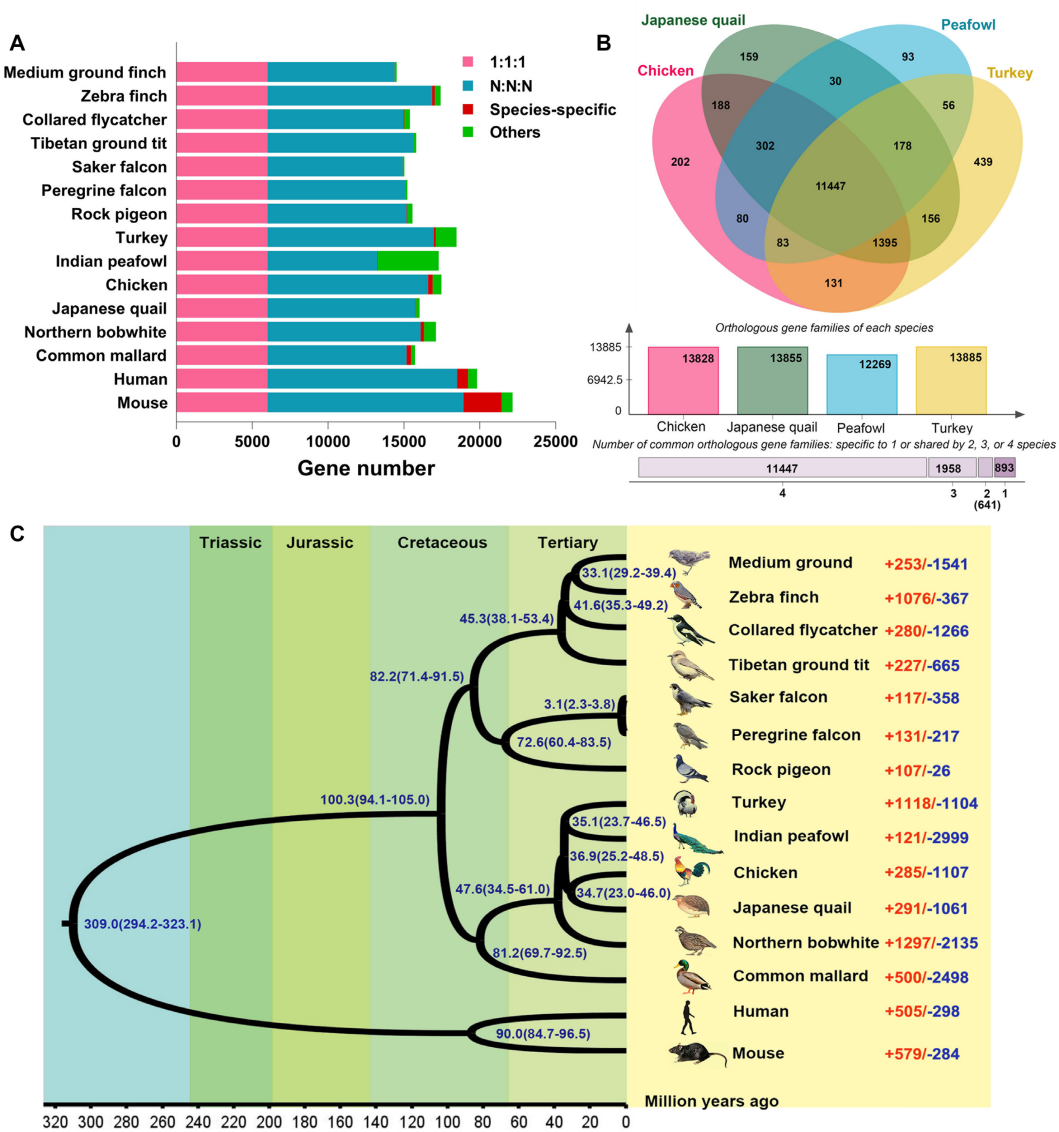
Gene family and genome evolution among the peafowl and 14 other species. **A**, Statistics of orthologs among 15 species. “1:1:1” indicates that the single-copy orthologs are shared by 15 species with 1 copy. “N:N:N” represents any other orthologous group (missing in 1 species). Species-specific shows the specific orthologs in each species. Other orthologs are unclustered into gene families. **B**, Venn diagram of the shared orthologous gene families among the Phasianidae species (peafowl, Japanese quail, chicken, and turkey). The numbers represents the unique or common gene family among the species. **C**, The phylogenetic relationship tree among 15 species was constructed by maximum likelihood with JTT model based on the single-copy orthologous sequences, with human and mouse as outgroups. The divergence time of species was estimated by 5 calibration times from the TimeTree database, including human–mouse (85–97 Mya), human–zebra finch (294–323 Mya), zebra finch–medium ground finch (30.4–46.8 Mya), common mallard–zebra finch (93.2–104.6 Mya), and saker falcon–peregrine falcon (1.66–3.68 Mya). Of them, the divergence time of human and mouse is used as a comparison at the bottom of the figure, with periods such as Tertiary, Cretaceous, Jurassic, and Triassic indicated by different colours. In addition, the expansion and contraction of the gene families in 15 species are indicated to the right of the species name. The red (+) and blue (−) numbers represent the expanded and contracted genes, respectively.

The protein sequences of 15 species were used to search the orthologues using OrthoFinder [88]. The results showed that a total of 18,038 orthogroups were identified in 15 species, of which 5,999 single-copy orthologues were shared among these species (Fig. 3A). In addition, 93 gene families were specific to peafowl and 11,447 gene families were shared by peafowl and other Phasianidae (chicken, turkey, and Japanese quail; Fig. 3B). The peafowl species-specific gene families were mainly involved in immune response and biological process; e.g., *FOXP3, FZD3*, and *TP53* participate in many immunological processes and play an important role in melanoma and bone homeostasis (Supplementary Table S12) [89–91].

We concatenated 5,999 single-copy orthologues of 15 species and then aligned them to construct a phylogenetic tree with a bootstrap value of 1,000 using the maximum likelihood method (Supplementary Figs S5 and S6). The results show that the Galliformes order is clustered, within which the Phasianidae family formed a group. Moreover, peafowl were found to be closer to turkey than chicken in the Phasianidae family; these findings are inconsistent with those reported by Jaiswal et al. [15]. We found that the relationship of chicken and quail was closer than turkey; white duck, belonging to the Anseriformes order, was closer to the Galliformes order (Fig. 3C). Additionally, the divergence time of all species was estimated and calibrated through the divergence time between human and mouse, human and zebra finch, zebra finch and medium ground finch, common mallard and zebra finch, and saker falcon and peregrine falcon from the TimeTree database. The divergence between Galliformes and Anseriformes was estimated to be 81.2 million years ago (Mya). The divergence between the northern bobwhite and Phasianidae family is represented by the calibration point of northern bobwhite and turkey. The divergence between the peafowl and turkey was ∼35.1 Mya, sharing a common ancestor with the chicken ∼36.9 Mya (Fig. 3C). However, divergence between chicken and Japanese quail was estimated to be 34.7 Mya within the range of divergence (33.2–42.3 Mya) according to TimeTree [92], suggesting that the relationships between the common ancestor of peafowl and turkey, and chicken and Japanese quail were very close, as well as the relationship between these 4 species. The divergence of pheasant birds took place in the Tertiary era; this marks the advent of the modern biological era, which was the peak period of divergence for animals and plants. At this time, new generation replaced the ancient types, with an increase in the number of similar species, the common and diverse divergence of birds, and a rapid evolution of more species.

### Genome synteny and collinearity among the Indian peafowl, chicken, and turkey

**Figure 4:**
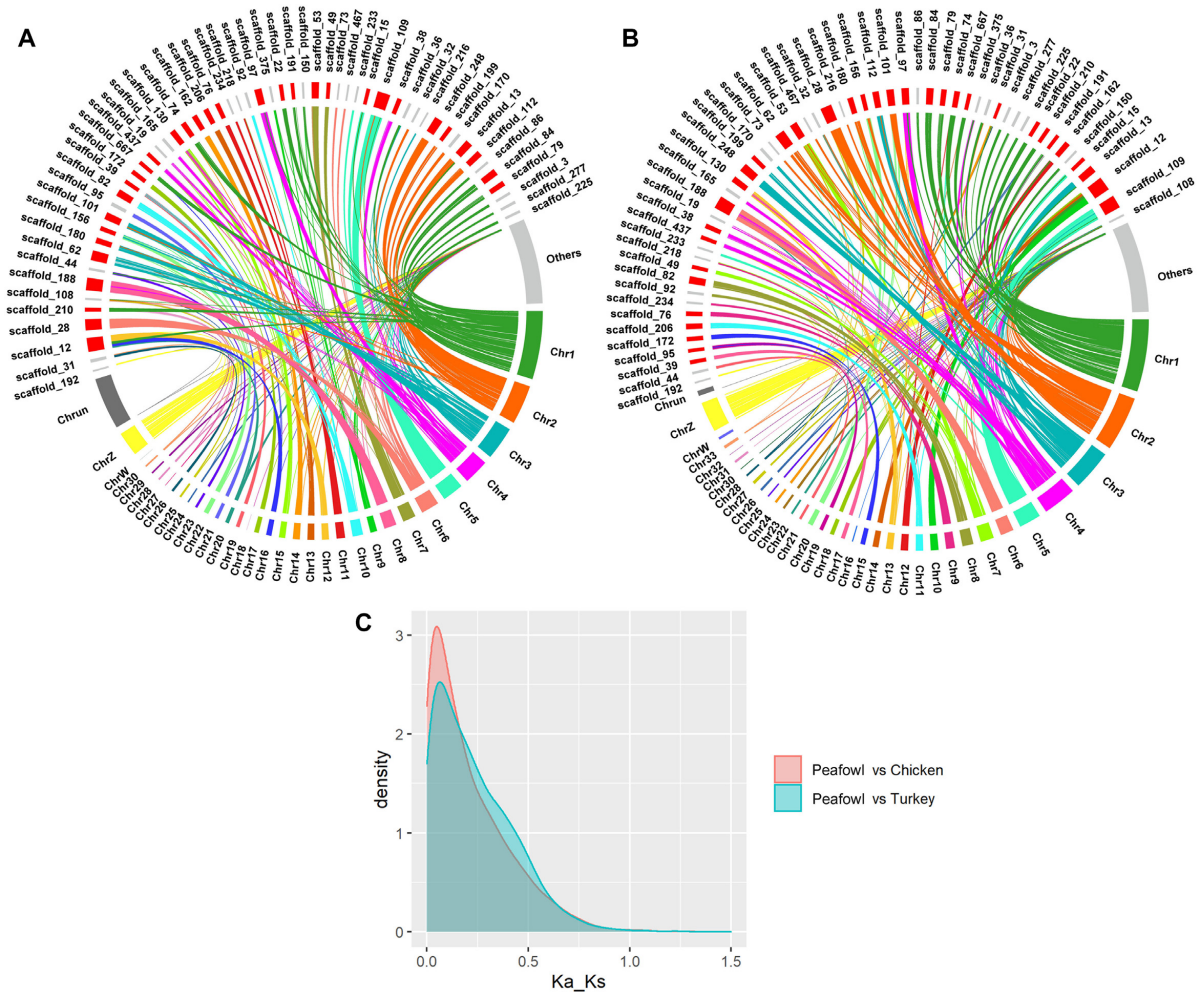
Genome synteny and collinearity among the Indian peafowl, chicken, and turkey. **A**, A syntenic map of the peafowl and turkey genomes. The perimeter of the ring represents the length of chromosomes labelled by different colours or scaffolds. It displays the scaffolds with length >5 Mb (scaffolds N70) of the assembled Indian blue peafowl, of which, the first 32 scaffolds are indicated in red and other scaffolds in gray. **B**, Syntenic map of the peafowl and chicken genomes. The first 35 scaffolds are indicated in red, and other scaffolds in gray. **C**, Distribution of Ka/Ks ratio in the genomes of peafowl, turkey, and chicken.

Collinearity analysis can reflect the homology of different species and genetic relationships. Genes with a pairwise ratio of nonsynonymous to synonymous substitutions (*dN*/*dS*) could be used to infer positive selection and contribute to understanding the evolutionary characteristics in species. In this study, pairwise synteny was compared between peafowl and chicken, and peafowl and turkey, and the ratio of *dN*/*dS* was calculated. Scaffold lengths greater than scaffold N70 (5 Mb) in the peafowl genome and other collinear scaffolds were marked as others were displayed (Fig. 4A and B). Moreover, the distribution density of the *dN*/*dS* ratio was calculated and is shown in Fig. 4C. Ninety-seven positively selected genes (*dN*/*dS* > 1) in peafowl compared to chicken were associated with biological processes and immune-related pathways (*IL4, CD3D, CD3E*, and *HLA-DMB*) (*P* < 0.05); e.g., T helper 1 (T_h_1) and T_h_2 cell differentiation, T-cell receptor signaling pathway, and intestinal immune network for IgA production. Furthermore, compared with turkey, 43 positively selected genes were notebly enriched in GO terms of organelle (GO:0043226), extracellular space (GO:0005615), and epithelium migration (GO:0090132), and the pathways of glutathione metabolism (*GPX1, GPX2*, and *GPX4*) and thyroid hormone synthesis (*GPX1, DUOXA2*, and *GPX2*) (*P* < 0.05) (Supplementary Tables S13 and S14), which are involved in gastrointestinal health, anti-stress, growth development, and metabolism. Notably, as a common positive selection gene, *EDN1* was reported to participate in many biological processes, such as epithelium migration and differentiation, pigmentation, and their receptors (EDNRs) widely distributed in various tissues in chicken [93]. These enrichment results indicated that the positively selected genes in peafowl were mainly related to intestinal immunity, anti-stress, growth development and metabolism, and pigmentation, compared with turkey and chicken in the evolutionary process. These features were beneficial for peafowl to enhance adaptability, improve disease resistance and anti-stress ability, enrich plumage colour, and better adapt to the living environment during long-term artificial breeding.

### Gene family expansion and contraction across the Indian peafowl genome

Likelihood analysis could identify the evolutionary rate and the notable expansion and contraction of gene families in species [79]. In this study, changes of gene family in peafowl were examined with a likelihood ratio test. Compared to the gene families in other species, the results suggest that 121 expansions and 2,999 contractions of gene families (*P* < 0.05) were detected in peafowl (Fig. 3C), of which, 21 significantly gained genes were mainly involved in energy metabolism and storage (*GIMAP1, GIMAP2*, and *GIMAP8*) and immune response (*CD244*) (*P* < 0.05), such as the GO terms of natural killer cell activation involved in immune response (GO:0002323), MHC class I protein binding (GO:0042288), positive regulation of interleukin-8 production (GO:0032757), positive regulation of interferon-γ production (GO:0032729), and lipid droplet (GO:0005811) (Supplementary Table S15). Conversely, 23 significantly contracted genes were mainly relevant to biological processes such as fatty acid degradation (*ALDH3A2*) (GO:0001561), myocardium development (GO:0048739), muscle contraction and cardiac disease (*MYH6, MYH7*, and *MYH7b*), olfactory receptor activity (*OR52B2, OR52K1*, and *OR4S1*) (GO:0004984), and the pathways of olfactory transduction, metabolism, and cardiac muscle contraction (Supplementary Table S16). For example, the expression of *MYH6* and *MYH7* directly dictates the slow- or fast-twitch phenotype in skeletal muscle and plays a vital role in cardiomyocyte energetics and metabolism [94, 95]. The olfactory genes were importantly characteristic during adaptive evolution in birds [96]. During their long-term domestication, peafowl have been artificially raised and fed a manufactured diet; as a result, their ability to find food and fly has declined, which likely caused the contraction of genes related to the sense of smell and the regulation of skeletal muscle movement. In addition, we observed that Phasianidae had a higher rate of birth and death than that of the other 2 branches, indicating that this family underwent a rapid evolution.

### Positively selected genes in the Indian peafowl genome

To reveal the adaptive divergence and evolution of peafowl, positive selection was analysed by using the branch-site model in the CODEML program. Significantly positive sites were evaluated by BEB values (BEB ≥ 0.95), which demonstrated that the sites were under positive selection in branch-site model A (foreground). In the branch of peafowl (foreground), 3,417 genes were under significantly positive selection based on BEB values (*P* < 0.05). These genes were annotated and classified through the analysis of GO ontology and KEGG pathways to further explore the impact of adaptive evolution on peafowl. According to the results of functional enrichment analyses, we briefly summarized that these positively selective genes mainly participated in the process of lipid metabolism (i.e., GO:0005811, GO:0030169, and GO:0008289), limb and skeletal development (i.e., GO:0060173, GO:0001503, and GO:0030509), immune response (i.e., GO:0070498, GO:0043123, and GO:1901224), pigmentation (GO:0042470 and GO:0030318), sensory perception (i.e., GO:0008542, GO:0008542, and GO:0007605), and other GO terms (Supplementary Table S17). Additionally, the pathways of positively selected genes were notably enriched in metabolic pathways, PI3K-Akt signaling pathway, NF-κB signaling pathway, pathways in cancer, MAPK signaling pathway, TNF signaling pathway, Jak-STAT signaling pathway, mTOR signaling pathway, FoxO signaling pathway, fatty acid metabolism, IL-17 signaling pathway, cholesterol metabolism, Ta_h_17 cell differentiation, and so on (Supplementary Table S18), which were mainly associated with immunity, energy metabolism, and cell growth and differentiation.

The branch model was used to identify a total of 10 rapidly evolving genes in peafowl, including *BCL7A, MEF2C, MED27, COPS7A, NMNAT2, SLC25A25, TNIP2, ETS1, CCDC6*, and *GSG1L*. Functional enrichment showed that significant pathways included those pathways in cancer; nicotinate and nicotinamide metabolism; thyroid cancer; renal cell carcinoma; parathyroid hormone synthesis, secretion, and action; thyroid hormone signalling pathway; apelin signalling pathway; fluid shear stress; and atherosclerosis (*P* < 0.05). Significant GO terms were involved in melanocyte differentiation, skeletal muscle and bone development, immunity, and response to stress (Supplementary Table S19). Notably, *MEF2C* was involved in most GO terms and pathways and played a vital role in bone and muscle development, immunity, and melanocyte differentiation; therefore, it may have been an important gene in the rapid evolution of peafowl [97–99].

### Genes with allele frequency between blue and leucism plumage in Indian peafowl

**Figure 5:**
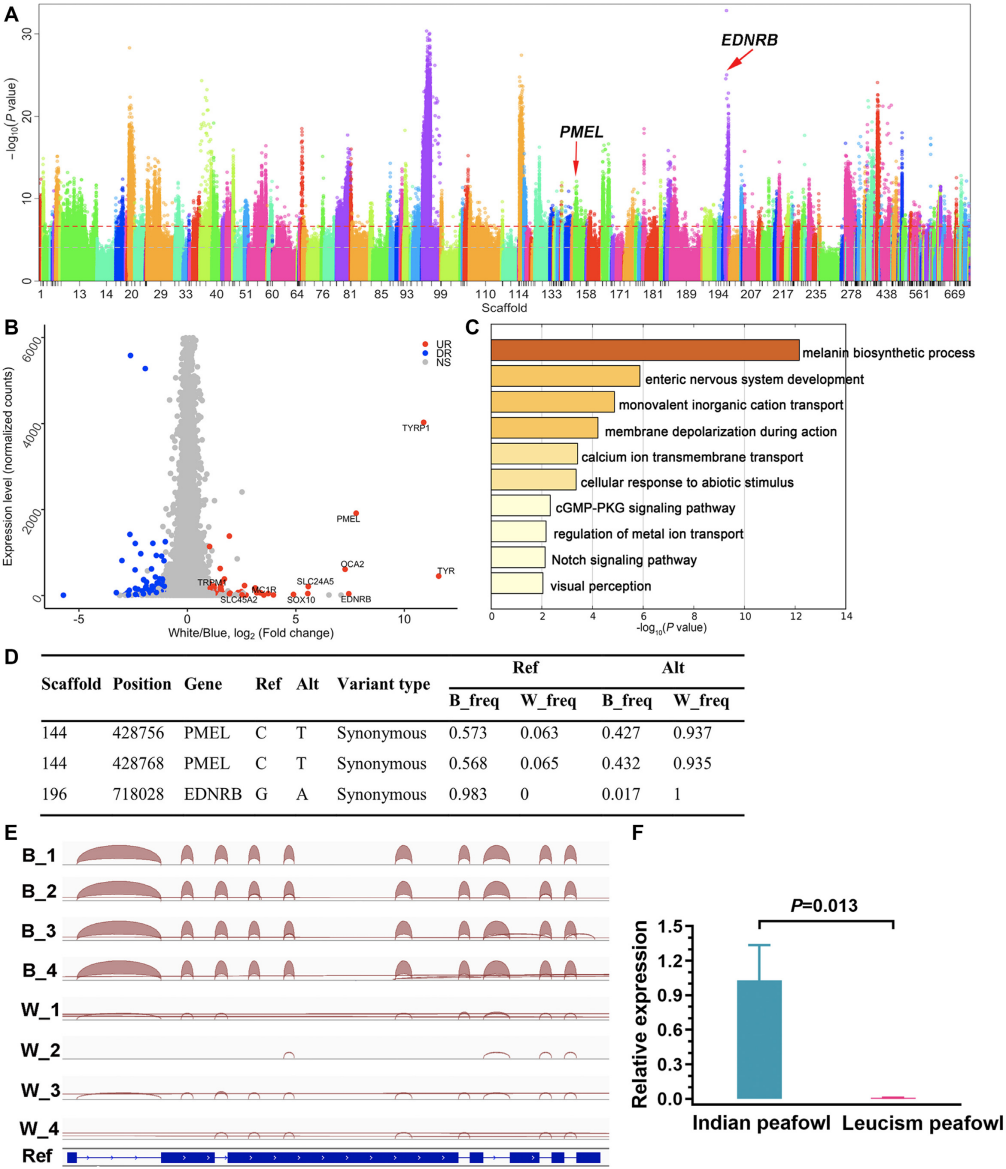
Causal genes for leucism plumage in blue and leucistic peafowl. **A**, Allele frequency differences between blue and leucistic peafowl. Scaffolds are distinguished by different colours. The candidate SNPs along with causal genes are marked by arrows, including *EDNRB* and *PMEL*. **B**, Differentially expressed genes (DEGs) related to the plumage pigmentation. The red and blue dots indicate up- and downregulated genes in blue and leucistic peafowl, respectively. A total of 69 downregulated genes and 52 upregulated genes were identified, of which 10 upregulated genes were associated with the melanin deposition, and so marked out. **C**, KEGG and GO enrichment of DEGs related to the plumage pigmentation in blue and leucistic peafowl. The darker the colour, the more significant the difference. The top significant pathway was enriched in the process of melanin synthesis based on the criterion of *P* < 0.05 as significant. **D**, Allele frequencies of DEGs in blue (B) and leucistic (W) peafowl. The top 2 differential sites were located in *PMEL* and *EDNRB*. **E**, *PMEL* transcripts in the feather pulp of blue (B) and leucistic (W) peafowl. The RNA sequencing reads of *PMEL* were aligned to the assembly peafowl genome in the feather tissue of blue and leucistic peafowl. The red arc represents the mRNA expression level of *PMEL*. Apparently, *PMEL* was normally expressed in blue peafowl but almost not expressed in leucistic peafowl. **F**, RT‐qPCR of *PMEL* transcripts in the feather pulp of blue and leucistic peafowl. The relative expression of *PMEL* was significantly decreased in leucistic peafowl compared to the mRNA expression of *PMEL* in blue peafowl (*P* = 0.013).

To localize the genomic region underlying plumage colour, the allele frequency between blue and leucistic peafowl was analysed. The clean data of 2 pooled resequencings were aligned to the assembled peafowl genome using Samtools with option “mpileup” and filtered to calculate allele frequency differences using Population2 software [100]. The significance of allele frequency differences was estimated by Fisher exact test. Upstream and downstream of 50 kb with a −log10 (*P*-value) >30 were extracted as potential candidate regions. As a result, we found that *EDNRB* in scaffold 196 and *PMEL* in scaffold 144 were significantly related to plumage pigmentation (Fig. 5A). Additionally, based on RNA-seq data, 69 downregulated genes and 52 upregulated genes between blue and leucistic peafowl were detected, of which 10 upregulated genes (*TRYP1, TYR, PMEL, EDNRB, OCA2, SLC24A5, SOX10, MC1R, SLC45A2*, and *TRPM1*) were associated with melanin deposition (Fig. 5B). The functional enrichment of DEGs showed that the most significant pathway was enriched in the process of melanin synthesis (*P* < 0.05; Fig. 5C). To further investigate differences in allele imbalance in DEGs, we used resequencing data to identify the allelic imbalance by calculating the allele frequency of 10 pigmentation-related genes in the blue and leucistic peafowl and annotated the function of sites using snpEff software [101]. Only 2 differential sites were located in *PMEL* and 1 in *EDNRB*, but none of the differential sites were obviously functional mutations, such as missense mutations, splicing mutations, or nonsense mutations (Fig. 5D). Collectively, overlapping with these results based on resequencing and RNAseq data, we determined that the formation of leucistic plumage was most likely related to the differential expression of *PMEL* and *EDNRB* in peafowl.

### Candidate causative gene for the leucism plumage phenotype in blue and leucism peafowl

To detect the *PMEL* and *EDNRB* transcripts in blue and leucistic peafowl, we examined the RNA-seq data of *PMEL* and *EDNRB* using the integrative genomics viewer (IGV) application. The results indicated that there was no difference in the transcript of *EDNRB* in the 2 types of feather pulp (Supplementary Fig. S7), suggesting that *ENDRB* was normally expressed in blue and leucistic peafowl. Compared to the transcript of *PMEL* in blue peafowl, we found that this gene was hardly expressed in leucistic peafowl (Fig. 5E). Moreover, to further determine the mRNA expression of *PMEL* in leucistic peafowl, reverse transcription quantitative PCR (RT-qPCR) of *PMEL* was conducted in blue and leucistic peafowl (Supplementary Table S20). RNA samples were extracted from feather pulp and used for subsequent PCR. Surprisingly, we observed that the mRNA expression of *PMEL* in leucistic peafowl was significantly reduced in comparison to that in blue peafowl (*P* = 0.013; Fig. 5F), which was consistent with the results of RNA-seq data. Hence, we confirmed that *PMEL* was a strong candidate causative gene for the formation of leucism plumage in blue and leucistic peafowl. Further investigations are needed regarding the mechanism for the downregulated expression of *PMEL* in leucistic peafowl.

## Discussion

With the development of sequencing technology, the reduction of sequencing costs, and the improvement of assembly methods, an increasing number of genome sequence maps of various species have been published, making whole-genome sequencing an important method for conducting basic genetic research on species. Recently, many avian genomes have been assembled, providing excellent material from which to study the genetic mechanisms of evolution, behaviour, and pathology. In this study, 3 sequencing strategies were combined to construct the Indian peafowl genome, and 1.05 Gb total draft genome sequence was obtained, with a sequencing depth of up to 362×. Moreover, the lengths of contig N50 and scaffold N50 were, respectively, achieved at 6.2 and 11.4 Mb, which was close to the chromosomal level. Compared with other avian genomes and the draft genomes of peafowl assembled by Jaiswal et al. and Dhar et al. [15, 16] the present Indian peafowl genome showed a notable improvement of assembly quality, including consistency, accuracy, and integrity. This draft genome of peafowl is a considerable improvement in terms of the quality of genome assembly and strongly supports the subsequent comparative genomic analysis.

In recent years, since the rapid development of genomics and the accumulation of genomic data, comparative genomics has become a research hot spot that can now explain biological functions and evolutionary characteristics at a genome-wide level. In particular, avian genomes are favoured for the investigation of adaptive evolution and species-specific biological characteristics by discovering novel genes and gene function through comparative genomics analysis. In this study, comparative genomics analysis was conducted on peafowl and other avian species to explore the unique biological characteristics of peafowl during evolution. First, the construction of phylogenetic relationships is key and a basis for many comparative genomic analyses. Generally, most phylogenetic relationships of birds are constructed on the basis of mitochondrial DNA, the cytochrome b gene, nuclear genes, or a combination of these [102–104]. Meanwhile, many studies using different data types to construct the tree have shown that there are controversial uncertainties in the phylogenetic classification of birds and that more evidence is needed for verification. In this study, single-copy homologous amino acid sequences from whole-genome sequencing data were used to construct a phylogenetic tree of 15 species; results suggested that the position of peafowl was closer to that of turkey than to chicken. This result was similar to those of previous studies [16, 15]. Moreover, the estimated divergence time between peafowl and turkey was near the divergence time among chicken and the ancestors of peafowl and turkey, and belonged to the Tertiary era. We speculated that the number of species and outgroup or dataset have an effect on phylogeny and divergence time [105]. Notably, we observed that *FOXP3* and *TP53* were mainly species-specific genes of peafowl compared to other Phasianidae. *FOXP3* is necessary for the development of regulatory T lymphocytes and is essential for maintaining immune homeostasis and immune self-tolerance to environmental antigens by eliminating natural reactive T cells in the thymus and peripheral organs. Meanwhile, *FOXP3* plays an important role in bone and haematopoietic homeostasis, inflammatory bone loss diseases, and abnormal bone weight, which can affect lymphoid haematopoiesis by acting on the development and function of osteoclasts [90]. *TP53* plays an important role in inhibiting the progression of bone and soft-tissue sarcoma. The loss of *TP53* activity can promote the osteogenic differentiation of bone marrow stromal cells and the development of osteosarcoma of these cells, which can prevent their malignant transformation [89]. In this study, the enrichment of these genes specific to chicken and turkey in peafowl showed that a healthy development and immunity of bones was important in peafowl evolution because this was conducive to achieving breeders' demand for rapid growth, large size, and strong disease resistance in domestication.

Species-specific immune-related genes are always positively selected in the adaptive evolution of many species. In this study, the number of GO terms and pathways related to immunity in peafowl was greater than that of others, such as the expansive genes and rapidly evolving genes involved in the process of MHC class I protein binding, TNF signalling pathway, NF-κB signalling, IL-17 signalling pathway, and T_h_17 cell differentiation. Likewise, we found that many olfactory genes and myosin genes were lost in peafowl. Myosin is a functional and structural protein that directly regulates muscle contraction, movement, and cardiac function in animals [106]. Olfaction plays a crucial role in avian life, contributing to the recognition of food, courtship, or the detection of danger [107, 108]. Birds can recognize close relatives to avoid inbreeding and distinguish the direction of migration by using their acute sense of olfaction [109, 110]. However, peafowl in this study were artificially farmed and the manufactured feed supplied throughout domestication has caused a gradual degradation of their ability to find food in the wild and to fly, which may explain the loss of myosin family genes and olfactory family and contribute to reducing energy expenditure.

Most birds have a small body size owing to the selective pressure to reduce body weight and energy expenditure [111]. However, the peafowl is well known to have a large body size, huge tail, and beautiful plumage, all of which are likely to have gradually evolved owing to better adaption to ecological environment. In this study, the enrichment analysis of positive selection genes mainly yielded those involved in skeletal development, bone morphology, and energy metabolism and storage, such as the mTOR signalling pathway, MAPK signalling pathway, BMP signalling pathway, limb development, lipid droplet, and lipid binding. mTOR is a central integrator of cellular growth and metabolism, and the mTOR signalling pathway plays a vital role in innate and adaptive immune responses and the regulation of energy balance [112, 113]. BMP is an important member of the transforming growth factor-β (TGF-β) superfamily through regulating the activity of downstream genes to participate in many important biological processes, such as nervous system differentiation, tooth and bone development, and cancer [114, 115]. The MAPK signalling pathway also participates in the regulation of feather growth and development [116]. Moreover, we observed that *MEF2C*, as a rapidly evolving gene, could regulate muscle and cardiovascular development and not only is a core component of development in regulating muscle, nerve, cartilage-like, immune, and endothelial cells but is also necessary for normal chondrocyte hypertrophy and ossification [131, 132]. Cartilage formation is a key process in vertebrate bone development and health maintenance, and most bones are developed through cartilage ossification. Potthoff et al. suggest that *MEF2C* can directly regulate transcription of the myosin gene, and the loss of *MEF2C* in skeletal muscle causes improper sarcomere organization, which reveals the key role of *MEF2C* in maintaining sarcomere integrity and skeletal muscle maturation after birth [33]. Arnold et al. indicate that the transcription factor *MEF2C* could regulate muscle and cardiovascular development, and control skeletal development by activating the genetic program of chondrocyte hypertrophy [134]. Hence, in this study, we found that *MEF2C* underwent rapid evolution in peafowl and may be conducive to the development and morphology of bones and the maintenance of body shape. This may well explain the evolutionary phenotype characteristics of the increasing weight and body size of peafowl in order to meet breeders' needs during domestication. Furthermore, the iridescent plumage and long tail are also attractive. Many positively selected genes associated with pigmentation, such as *TYR, SZT2, NF1, ARCN1, KIT, HPS5, FIG4, LYST, RACK1, USP13, HPS6, OCA2, MITF*, and *BCL2* were also identified. All the above results contribute to understanding the phenotypic characteristics, such as large body size, long tail, and dazzling plumage in peafowl, during evolutionary adaptation.

To date, a number of studies examining the genetic mechanism of plumage colour in avians have been reported [117, 118]. In the present study, the mechanism behind the leucism plumage phenotype in peafowl was explored, combining transcriptome analysis and RT-qPCR with resequencing data. Plumage colours are often determined by causal genes that may have a difference in allele frequency between different plumage colour populations. We used resequencing data to make selective signal analysis by detecting the allele frequency difference in blue and leucistic peafowl; results suggested that only *PMEL* and *EDNRB* were involved in pigmentation. Meanwhile, we used RNA-seq data to determine the DEGs between blue and leucistic peafowl, and results indicated that 10 significantly upregulated genes were associated with melanin deposition. Subsequently, we used resequencing data to identify the allele imbalance difference sites of 10 upregulated genes and found that only *PMEL* and *EDNRB* had differential sites. Although there were many significant sites in the allele frequency difference, they did not cause differences in transcripts and resulted in the differential expression of related genes in the analysis of DEGs, with the exception of *PMEL* and *EDNRB*. By overlapping the results based on resequencing and RNA-seq data, we determined that *PMEL* and *EDNRB* were candidate genes for the formation of leucism plumage in peafowl. Furthermore, we observed the transcripts of *PMEL* and *EDNRB* based on RNA-seq data by IGV visualization and discovered that *PMEL* was hardly expressed in leucistic peafowl compared to blue peafowl; moreover, *EDNRB* was normally expressed in both variants. Finally, we verified the low mRNA expression of *PMEL* in leucistic peafowl by RNT-qPCR; this result suggested that *PMEL* was a strong candidate causative gene for the formation of leucism plumage. The formation and deposition of melanin mainly occurs on the amyloid fibres of melanosomes. As a key signal molecule, *PMEL* could directly initiate the formation of melanosomes and promote their synthesis [119]. Moreover, many studies reported that mutations of *PMEL* could cause its low expression, leading to decreased melanogenesis and further resulting in hypopigmentation phenotypes in animals like silver horses, white chicken, and yellowish Japanese quail [120–122]. Here, we detected that the low expression of *PMEL* was associated with leucism plumage in peafowl. However, to further investigate the causal mutations of *PMEL* low expression, the mutations of *PMEL* were examined and annotated, but no functional mutation sites were found. We hypothesized that the low expression of *PMEL* transcription was probably caused by changes in regulatory elements located in the upstream 5 kb promoter region of *PMEL* and thus impeded melanin synthesis. Unfortunately, there were no mutations in the core promoter region and transcription factor binding sites predicted by promoter prediction websites. In addition, resequencing data were also used to detect the structural variation of the *PMEL* gene and its upstream region. Moreover, the transcriptome data were used to detect SNP and Indel variation, as well as PCR amplification of the *PMEL* gene and its upstream 5 kb promoter region using Sanger sequencing; however, no possible variations were found. In view of these findings, we speculated that the *PMEL* gene was likely to exist as a complex structure because it could not be completely measured through sequencing and the causal sites were not identified; this needs further exploration. Nevertheless, for the first time to our knowledge, we identified that *PMEL* is a causal gene of leucism plumage, providing a novel insight into the formation of the leucism phenotype in blue and leucistic peafowl. The results revealed the genetic mechanism of leucism plumage at the whole-genome transcriptome level.

## Conclusion

This study performed an improved assembly of higher quality and greater sequencing depth of the peafowl genome. First, the assembled genome is superior to 2 previous draft genomes of peafowl, in terms of both the sequencing depth and assembly quality. Second, based on the draft genome, the study determined that peafowl are closer to turkey than chicken at the genome-wide level. Moreover, the comparative genomic analysis indicated that the evolution of Indian peafowl metabolism, immunity, skeletal development, and feather development may be related to the unique characteristics of peafowl in domestication; this investigation was conducted to provide baseline information about the phenotypic evolution of peafowl. Finally, the study was the first to report a combination of resequencing and transcriptome analysis in Indian peafowl and to reveal the molecular mechanism of leucism plumage formation. Altogether, the present study provides a novel reference genome of the systematic evolution of peafowl and other birds that can assist in understanding the formation of plumage colouration and suggests new theories for the artificial breeding of peafowl.

## Data Availability

The whole-genome sequence data reported in this article have been deposited in the NCBI under accession number No. PRJNA745383. The resequencing raw data have been deposited in the NCBI SRA [124] under accession No. PRJNA665082. The transcriptomic raw data have been deposited in the NCBI under accession No. PRJNA661158. All supporting data and materials are available in the *GigaScience* GigaDB database [125].

## Additional Files

**Supplementary Figure S1**. Pipeline of the draft genome assembly of Indian blue peafowl

**Supplementary Figure S2**. Workflow of the genome annotation of Indian blue peafowl

**Supplementary Figure S3**. 17-kmer frequency distribution of peafowl genome

**Supplementary Figure S4**. Divergence distribution of transposable element of peafowl genome by using RepeatMasker software

**Supplementary Figure S5**. Phylogenetic tree of 15 species constructed with IQ-tree

**Supplementary Figure S6**. Phylogenetic tree of 15 species constructed with RAxML

**Supplementary Figure S7**. EDNRB transcripts in the feather tissue of peafowl by IGV visualization

**Supplementary Table S1**. Statistics of genome assembly data of peafowl

**Supplementary Table S2**. Summary of*de novo* genome assembly of peafowl

**Supplementary Table S3**. Percentage of the base contents of peafowl genome

**Supplementary Table S4**. Statistics of paired-end read mapping in peafowl genome

**Supplementary Table S5**. Number of SNPs of peafowl genome

**Supplementary Table S6**. Assembly assessment of completeness by using BUSCOs

**Supplementary Table S7**. Whole-genome repetitive sequences of Indian peafowl genome predicted by homologous alignment and *de novo* search

**Supplementary Table S8**. Composition of repetitive sequences in peafowl genome

**Supplementary Table S9**. Prediction of protein-coding genes for peafowl genome

**Supplementary Table S10**. Statistics of functional annotation of protein-coding genes in the peafowl genome assembly

**Supplementary Table S11**. Statistics of non-coding RNAs in the assembly of peafowl

**Supplementary Table S12**. Functional enrichment of species-specific genes in peafowl compared with the Phasianidaes (chicken, turkey, and Japanese quail)

**Supplementary Table S13**. Functional categories of positively selected genes (*dN*/*dS* > 1) between peafowl and chicken

**Supplementary Table S14**. Functional categories of positively selected genes (*dN*/*dS* > 1) between peafowl and turkey

**Supplementary Table S15**. Functional enrichment of significantly expansive genes in peafowl

**Supplementary Table S16**. Functional enrichment of significantly contractive genes in peafowl

**Supplementary Table S17**. GO term enrichment of positively selected genes in peafowl under branch-site model

**Supplementary Table S18**. KEGG pathways of positively selected genes in peafowl under branch-site model

**Supplementary Table S19**. Functional categories of positively selected genes in peafowl under branch model

**Supplementary Table S20**. Primer sequences of *PMEL* for RT-qPCR

giac018_GIGA-D-21-00190_Original_Submission

giac018_GIGA-D-21-00190_Revision_1

giac018_GIGA-D-21-00190_Revision_2

giac018_Response_to_Reviewer_Comments_Revision_1

giac018_Response_to_Reviewer_Comments_Revision_2

giac018_Reviewer_1_Report_Original_Submission Dustin Rubenstein -- 8/17/2021 Reviewed

giac018_Reviewer_1_Report_Revision_1 Dustin Rubenstein -- 11/17/2021 Reviewed

giac018_Reviewer_2_Report_Original_Submission Yang Liu, Ph.D -- 9/20/2021 Reviewed

giac018_Reviewer_2_Report_Revision_1 Yang Liu, Ph.D -- 11/28/2021 Reviewed

giac018_Reviewer_2_Report_Revision_2 Yang Liu, Ph.D -- 12/25/2021 Reviewed

giac018_Supplemental_Files

## Abbreviations

BEB: Bayesian empirical Bayes; BLAST: Basic Local Alignment Search Tool; bp: base pairs; BUSCO: Benchmarking Universal Single-Copy Orthologs; BWA: Burrows-Wheeler Aligner; cDNA: complementary DNA; CDS: coding gene sequence; CEGMA: Core Eukaryotic Genes Mapping Approach; DEG: differentially expressed genes; GATK: Genome Analysis Toolkit; Gb: gigabase pairs; GO: Gene Ontology; kb: kilobase pairs; KEGG: Kyoto Encyclopedia of Genes and Genomes; KOBAS: KEGG Orthology-Based Annotation System; MAFFT: multiple alignment fast Fourier transfom; Mb: megabase pairs; Mya: million years ago; NCBI: National Center for Biotechnology Information; PacBio: Pacific Biosciences; PASA: Program to Assemble Spliced Alignments; RAxML: Randomized Axelerated Maximum Likelihood; RNA-seq: RNA sequencing; SMRT: Single-Molecule Real Time; SNP: single-nucleotide polymorphism; SRA: Sequence Read Archive; TE: tandem repeat; tRNA: transfer RNA.

## Funding

This work was supported by Educational Commission of Jiangxi Province of China (GJJ190177) and by the Key Research and Development Program of Jiangxi Province of China (20171BBF60003).

## Ethics Approval and Consent to Participate

All procedures used for this study and involving animals fully complied with guidelines for the care and use of experimental animals established by the Ministry of Agriculture of China. The Animal Care and Use Committee of the South China Agricultural University approved this study.

## Competing Interests

The authors declare that they have no competing interests.

## Authors' Contributions

X.Y., H.M., and J.R. designed the study and wrote the manuscript. S.L. and H.C. analyzed the data and wrote the manuscript. S.L., H.C., H.M., and W.L. revised the manuscript. S.L., H.Z., and B.L. conducted the validation experiments. S.L., J.O., M.H., S.Z., S.X., H.T., Y.G., Y.X., D.C., K.C., H.M., and X.Y. collected samples and performed the sequencing and genotyping experiments. All authors contributed to and approved the final manuscript.
