# Supplementary material for: A high-quality assembly reveals genomic characteristics, phylogenetic status, and causal genes for leucism plumage of Indian peafowl
Source: Gigascience. 2022 Apr 6;11:giac018. doi: 10.1093/gigascience/giac018 (PMC8985102; doi:10.1093/gigascience/giac018)
Supplement: giac018_GIGA-D-21-00190_Original_Submission [file giac018_giga-d-21-00190_original_submission.pdf]

# GigaScience

# A high-quality assembly reveals genomic characteristics, phylogenetic status and causal genes for white feather of Indian peafowl

--Manuscript Draft--

| | |
|---|---|
| Manuscript Number: | GIGA-D-21-00190 |
| Full Title: | A high-quality assembly reveals genomic characteristics, phylogenetic status and causal genes for white feather of Indian peafowl |
| Article Type: | Research |
| Funding Information: | educational commission of jiangxi province of china — Dr. huirong Mao<br>key research and development program of jiangxi province — Dr. huirong Mao |
| Abstract: | Background<br><br>Indian peafowl ( Pavo cristatus ) attracts people's attention because of the exclusively dazzling phenotypic characteristics. However, little is known about the phenotypic evolution and phylogeny of Indian peafowl at the whole-genome level. So far, there has been no report on the genetic mechanism of the formation of white feather in white feather peafowl.<br><br>Results<br><br>This study assembled a draft genome of Indian peafowl with a genome size of 1.05 Gb (the sequencing depth is 362×), and N50 of the contig and scaffold was up to 6.2 Mb and 11.4 Mb, respectively. Compared with other birds, Indian peafowl changed in terms of metabolism, immunity, skeletal development and feather development, which provided a novel insight into the phenotypic evolution of peafowl, such as the large body size and feather morphologies. It was confirmed that the phylogeny of Indian peafowl was closer to that of turkey than that of chicken. Specially, it was identified that PMEL was a causal gene leading to the formation of white plumage in blue and white feather peafowl.<br><br>Conclusions<br><br>This study provides a peafowl genome with high-quality as well as a novel understanding in the phenotypic evolution and phylogeny of peafowl among other birds. The results contribute a valuable reference genome to the study of the avian genome evolution. In addition, the discovery of the genetic mechanism of white plumage not only is a breakthrough in the exploration of peafowl plumage, but also provides clues and new ideas for further investigations of the avian plumage coloration and artificial breeding in peafowl. |
| Corresponding Author: | huirong Mao<br>Jiangxi Agricultural University<br>Nanchang, Jiangxi CHINA |
| Corresponding Author Secondary Information: | |
| Corresponding Author's Institution: | Jiangxi Agricultural University |
| Corresponding Author's Secondary Institution: | |
| First Author: | Shaojuan Liu |
| First Author Secondary Information: | |
| Order of Authors: | Shaojuan Liu |
| | Hao Chen |
| | |

Powered by Editorial Manager® and ProduXion Manager® from Aries Systems Corporation

| | |
|---|---|
| | Jing Ouyang |
| | Min Huang |
| | Hui Zhang |
| | Sumei Zheng |
| | Suwang Xi |
| | Hongbo Tang |
| | Yuren Gao |
| | Yanpeng Xiong |
| | Di Cheng |
| | Kaifeng Chen |
| | Bingbing Liu |
| | Wanbo Li |
| | Xueming Yan |
| | huirong Mao |
| | Jun Ren |
| **Order of Authors Secondary Information:** | |
| **Additional Information:** | |
| **Question** | **Response** |
| Are you submitting this manuscript to a special series or article collection? | No |
| **Experimental design and statistics**<br><br>Full details of the experimental design and statistical methods used should be given in the Methods section, as detailed in our Minimum Standards Reporting Checklist. Information essential to interpreting the data presented should be made available in the figure legends.<br><br>Have you included all the information requested in your manuscript? | Yes |
| **Resources**<br><br>A description of all resources used, including antibodies, cell lines, animals and software tools, with enough information to allow them to be uniquely identified, should be included in the Methods section. Authors are strongly encouraged to cite Research Resource | Yes |

Powered by Editorial Manager® and ProduXion Manager® from Aries Systems Corporation

| | |
|---|---|
| Identifiers (RRIDs) for antibodies, model organisms and tools, where possible.<br><br>Have you included the information requested as detailed in our Minimum Standards Reporting Checklist? | |
| **Availability of data and materials**<br><br>All datasets and code on which the conclusions of the paper rely must be either included in your submission or deposited in publicly available repositories (where available and ethically appropriate), referencing such data using a unique identifier in the references and in the “Availability of Data and Materials” section of your manuscript.<br><br>Have you have met the above requirement as detailed in our Minimum Standards Reporting Checklist? | Yes |

*Powered by Editorial Manager® and ProduXion Manager® from Aries Systems Corporation*

Click here to access/download;Manuscript;Main Document.docx

# A high-quality assembly reveals genomic characteristics, phylogenetic status and causal genes for white feather of Indian peafowl

Shaojuan Liu[1#], Hao Chen[3#], Jing Ouyang[3], Min Huang[1], Hui Zhang[1], Sumei Zheng[1], Suwang Xi[2], Hongbo Tang[3], Yuren Gao[3], Yanpeng Xiong[3], Di Cheng[2], Kaifeng Chen[2], Bingbing Liu[1],Wanbo Li[4], Xueming Yan[3*], Huirong Mao[2*], Jun Ren [1*]

[1] College of Animal Science, South China Agricultural University, Guangzhou 510642, China

[2] School of Animal Science and Technology, Jiangxi Agricultural University, Nanchang 330045, China

[3] College of Life Science, Jiangxi Science & Technology Normal University, Nanchang 330013, China

[4] Key Laboratory of Healthy Mariculture for the East China Sea, Ministry of Agriculture and Rural Affairs, Jimei University, Xiamen 361021, China

[#] Both authors contribute equally to this paper.

[*] Corresponding author.

E-mail address: maohuirong82@hotmail.com (H. M); xuemingyan@hotmail.com (X. Y)

# Abstract

**Background**: Indian peafowl (*Pavo cristatus*) attracts people's attention because of the exclusively dazzling phenotypic characteristics. However, little is known about the phenotypic evolution and phylogeny of Indian peafowl at the whole-genome level. So far, there has been no report on the genetic mechanism of the formation of white feather in white feather peafowl.

**Results**: This study assembled a draft genome of Indian peafowl with a genome size of 1.05 Gb (the sequencing depth is 362×), and N50 of the contig and scaffold was up to 6.2 Mb and 11.4 Mb, respectively. Compared with other birds, Indian peafowl changed in terms of metabolism, immunity, skeletal development and feather development, which provided a novel insight into the phenotypic evolution of peafowl, such as the large body size and feather morphologies. It was confirmed that the phylogeny of Indian peafowl was closer to that of turkey than that of chicken. Specially, it was identified that *PMEL* was a causal gene leading to the formation of white plumage in blue and white feather peafowl.

**Conclusions:** This study provides a peafowl genome with high-quality as well as a novel understanding in the phenotypic evolution and phylogeny of peafowl among other birds. The results contribute a valuable reference genome to the study of the avian genome evolution. In addition, the discovery of the genetic mechanism of white plumage not only is a breakthrough in the exploration of peafowl plumage, but also provides clues and new ideas for further investigations of the avian plumage coloration and artificial breeding in peafowl.

**Keywords:** Indian peafowl; Genome assembly; Phylogeny; *PMEL*; White feather

# Introduction

*Pavo cristatus*, commonly called Indian peafowl or blue peafowl, is the king of birds, representing elegance, honour, beauty, luck, and romance in many Asian cultures (Figure 1a) [1, 2]. Peafowl, belonging to the Aves, Galliformes, Phasianidae, and Pavo, has two species: green peafowl and blue peafowl. Indian peafowl, as the national bird of India, has been widely distributed in Bangladesh, Bhutan, India, Nepal, Pakistan, and Sri Lankan [2, 3]. Commonly, the male Indian peafowl is more attractive than female because of its larger body size and more glittering plumage. Indian peafowl is one of the largest and most beautiful birds in pheasant as a valuable ornamental display with their fan-shaped crests, brightly blue plumage, and glaring tails. Moreover, Indian peafowl is well known as a protein resource, because their meat, internal organs, and bones have high nutritional values, even with medicinal values [4-6].

With the improvement of whole genome sequencing technology, more and more avian genomes were assembled, such as chicken (*Gallus gallus*) [7], turkey (*Meleagris gallopavo*) [8], duck (*Anas platyrhynchos*) [9], and other birds [10], which provided basic references for the study of phenotypic characteristics, evolution, economic traits, and environmental adaptation of birds. As for peafowl, the first draft genome of peafowl was released in 2018. However, the length of scaffold and contig N50 of the assembling were only 25.6 kb and 19.3 kb, respectively [11]. Subsequently, Dhar, et al. improved peafowl genome by using Illumina and Oxford Nanopore technology (ONT), and the length of scaffold N50 was up to 0.23 Mb [12]. The previous studies of peafowl focused on courtship behaviour [13], immunity [14], and productivity [15]. Additionally, most researches on the phylogenetic relationship of peafowl and the Phasianidae were based on the mitochondrial genomes, DNA transposable factors, and partial DNA nucleotide

sequences, and the conclusions of these researches are still controversial [16-18]. Therefore, a better quality assembly of peafowl genome is needed to provide baseline data for further studies on peafowl. It has been suggested that the grey peafowl pheasant (*Polyplectron bicalcaratum*) could be the ancestor of peafowl [19]. However, the phylogenetic relationships of peafowl with other pheasants are still unclear, though there have been several recent attempts to investigate it. Study on Hainan peafowl pheasant provided evidences to argue whether it is a subspecies of the grey peafowl pheasant [20]. Zhou et al. (2015) analysed the phylogenetic status of Indian peafowl in Phasianidae by using a complete mitochondrial genome, and found out its closest genetic affinity with green peafowl [16]. Sun et al. (2014) revealed the close relationships among *Pavo, Polyplectron* and *Argusianus* within the Phasianidae [21]. It is expected that a more clarified taxonomic status of the peafowl in Phasianidae can be investigated via the high-throughput sequencing technology.

Previous studies reported that there were many plumage colour mutants, including white, black, variegated, cameo, and oaten [22-24], among which, the most ornamental colour was the white plumage, belonging to leucism rather than albinism since the feather was white but the eyes had melanin pigmentation (Figure 1b). The inherited basis of plumage colour has attracted researchers for a long time. The first report on it suggested that the plumage phenotype of peafowl was determined by autosomal genes in a recessive model [24]. Another study verified that a single autosomal locus was in control of all plumage phenotypes in peacock, where the pied colour appeared in two heterozygous mutant alleles, with the black on recessive mutant allele, and the all-white plumage in homozygous mutant allele as the most dominant [23]. Nevertheless, further

studies on the genetic mechanism of the white plumage in peafowl are needed, i.e., to clarify the causative mutations of this phenotype.

Therefore, a high-quality (near-chromosomal) reference genome of Indian peafowl was constructed by using the third-generation de novo assembly technology. Based on the assembly, it was devoted to investigate the molecular evolution and phylogenetic classification of peafowl in the Phasianidae at the genome-wide level. Subsequently, comparative genomics analysis was performed to investigate the biological characteristics of evolution through comparing the genome of Indian peafowl with the high-quality genomes of other birds, human and mouse. Furthermore, the transcriptomic and pooled resequencing data were analysed to identify the genetic mechanism of the white plumage in Indian peafowl. This work will provide an updated understanding of and key reference for genomic characteristics, phylogenetic status and genetic mechanism of white feather in Indian peafowl.

## Materials and Methods

### Sample collection

All procedures used for this study and involved in animals fully complied with guidelines for the care and utility of experimental animals established by the Ministry of Agriculture of China. The ethics committee of South China Agricultural University approved this study. A blood sample was collected from a female Indian peafowl for genome assembling, and 51 blood samples from 35 blue feather peafowls and 16 white feather peafowls for pooled resequencing in Leping Sentai special breeding Co., Ltd in Jiangxi Province, China, under the principles and standards of animal welfare ethics. Meanwhile, two liver and two muscle tissues were sampled from a female Indian

peafowl to assist the process of assembling the Indian peafowl genome. Additionally, feather pulps from 8 blue and 8 white peafowls were collected for RNA-seq.

**DNA and RNA extraction**

Genomic DNA was extracted from blood samples by using a routine phenol-chloroform protocol. The concentration of the extracted DNA was evaluated by using a Nanodrop 2000 spectrophotometer (Thermo Fisher Scientific, Waltham, MA, USA), and diluted to a final concentration of 100 ng/μL. The integrity of DNA was checked via electrophoresis on 0.8% agarose gel. Total RNA of feather pulp was extracted by using TRIzol reagent (Thermo Fisher Scientific, Waltham, MA, USA). The purity and degradation of RNA was detected by Nanodrop 2000 spectrophotometer and agarose gel electrophoresis.

**De novo assembling of the Indian peafowl reference genome**

Library Preparation and Sequencing: Genomic DNA was used to make a 350 bp insert fragment libraries by using the Illumina TruSeq Nano method, starting with 100 ng DNA. Mate pair libraries were made by using the Nextera Mate Pair Sample Preparation Kit (Illumina) with the gel plus option, and sequenced by using Illumina NovaSeq 6000 platform. For PacBio sequencing, genomic DNA was sheared by a g-TUBE device (Covaris) with 20 kb settings for further preparing a 20 kb Single-Molecule Real Time (SMRT) bell, and then the single-molecule sequencing was completed on a PacBio RS-II platform. For 10X genomics sequencing, each GEM was amplified by PCR and added P7 sequencing adapters for Illumina sequencing.

Genome Assembly: The genome assembling of Indian peafowl was performed in five steps, which was illustrated in Supplementary Figure S1. The raw reads were

generated from two paired-end libraries sequenced on Illumina NovaSeq 6000 platform. The sequencing adapters, contaminated reads, and low-quality reads were removed by using megablast v2.2.26 [25]. The genome size was calculated by using the formula: Genome size = kmer_Number / Peak_Depth. Secondly, PacBio sequencing was used to control and correct errors. The error corrected data were assembled by falcon software [26], and the Overlap-Layout-Consensus algorithm was used to obtain the consensus sequences, which were then corrected by quiver software [27]. Combined with the second-generation sequencing data, the consensus sequences were recalibrated by using the pilon software [28] to improve the accuracy, and high-quality consensus sequences were obtained. Thirdly, the 10X genomics sequencing was used to assist the genome assembly. The 10X genomics library was sequenced to obtain linked-reads, which were aligned to the consensus sequences obtained from the PacBio sequencing assembly, and then linked reads were added to assemble the super-scaffolds by fragScaff software [29]. Fourthly, similar to the third step, Chicago sequencing data was used to assist assembly to map the draft genome assembly. Finally, the Illumina reads were mapped to the draft genome by using BWA (Burrows-Wheeler Aligner) [30]. Then, pilon (version 1.22) was used to correct the assembled errors based on the mapped results.

Consistency and completeness: The consistency and integrity of the assembled peafowl genome were separately assessed by using the BUSCO (Benchmarking Universal Single-Copy Orthologs) [31] and CEGMA (Core Eukaryotic Genes Mapping Approach) [32, 33], based on single-copy orthologues from the AVES (odb9) database. In order to evaluate the accuracy, integrity and sequencing uniformity of the genome assembly, small fragment library reads were selected and aligned to the assembled

genome by using BWA software. All the genomic sequences were generated by Novogene Inc, Beijing, China.

Genome Annotation: Genome annotation mainly included three aspects: repetitive sequence annotation, gene annotation (including gene structure prediction and gene function prediction) and non-coding RNA (ncRNA) annotation (Supplementary Figure S2). The repetitive sequence annotation included the annotation through homologous sequence alignment and ab initio prediction. The RepeatMasker and RepeatproteinMask software [34] were employed to identify known repetitive sequences against the RepBase library [35]. In ab initio prediction, LTR_FINDER [36], RepeatScout [37], and RepeatModeler [38] were used to establish the de novo repeat sequence library, and then repetitive sequences were predicted by Repeatmasker software. The Tandem Repeats (TEs) in the genome were found by Tandem Repeat Finder software [39]. In gene annotation, it mainly combined three prediction methods: homology-based prediction, de novo prediction, and other evidence-backed predictions. Homology-based prediction used the protein sequences of Gallus gallus, Meleagris gallopavo, Peking duck, Struthio camelus, Nipponia nippon, and Eastern Zhejiang white goose, downloaded from Ensembl (release 74), to align to the Indian peafowl genome by using TblastN [40]. Genewise [41] was then used to align to the matched proteins for a precise gene model.

In addition, Augustus [42], GlimmerHMM [43], Geneid [44], GenScan [45], and SNAP software [46] were used for the ab initio predictions of gene structures. The above predictions with transcriptome-based data being combined, EVidenceModeler software [47] was used to integrate the gene set and generate a non-redundant and more

complete gene set. Finally, PASA was used to correct the annotation results of EVidenceModeler for the final gene set. Gene function of the final gene set was annotated by using the protein database of SwissProt [48], NR [49], Pfam [50], KEGG [51], and InterPro [52]. tRNAscan-SE software [53] was used to search for the tRNA sequence of genome, with INFERNAL software (http://infernal.janelia.org/) [54] from Rfam [55] to predict miRNA and snRNA of genome.

**Gene family**

The amino acid sequences of the following were downloaded from NCBI database to identify the gene families and single-copy orthologous genes. They are: Japanese quail (*Coturnix japonica*) [56], chicken (*Gallus gallus*) [57], turkey (*Meleagris gallopavo*) [8], northern bobwhite (*Colinus virginianus*) [58], common mallard (*Anas platyrhynchos*) [59], zebra finch (*Taeniopygia guttata*) [60], collared flycatcher (*Ficedula albicollis*) [61], medium ground-finch (*Geospiza fortis*) [62], tibetan ground-tit (*Pseudopodoces humilis*) [63], rock pigeon (*Columba livia*) [64], peregrine falcon (*Falco peregrinus*) [65], saker falcon (*Falco cherrug*) [66], human (*Homo sapiens*) [67], and mouse (*Mus musculus*) [68]. The longest transcript of each gene was extracted and then the genes with the length of protein sequences shorter than 50 amino acids were filtered. Based on the filtered protein-coding sequences data set, Orthofinder v2.3.7 [69] was used to identify gene families and orthologous gene clusters of 15 species. The single-copy orthologous sequences from the gene families were aligned by using MAFFT v7.450 software [70], and then the poorly sequences were removed by using Trimal software with default parameters [71]. The final result was used as a single data set for the subsequent comparative genome analyses.

## Phylogenetic tree and divergence time

To determine the phylogenetic relationship of 15 species, IQ-tree v2.1.2 software was first used to find the best model for constructing phylogenetic tree with options "-m MF" and the species tree with bootstrap 1000 based on the concatenated alignment of single-copy orthologues sequences from 15 species [72]. RAxML software was used to construct phylogenetic tree with parameters "-m PROTGAMMALGX -f a" with bootstrap 1000. Divergence time of 15 species was estimated by using MCMCtree program implemented in PAML packages [73]. Five calibration time (human-mouse (85~97Mya), human-zebra finch (294~323Mya), zebra finch-medium ground finch (30.4~46.8Mya), common mallard-zebra finch (93.2~104.6Mya) and saker falcon-peregrine falcon (1.66~3.68Mya)) from TimeTree database [74] were used as constrains in the divergence time estimation. The MCMC process was run to sample 1,000,000 times, sample frequency set to 10, and burn-in 40,000, to finally achieve a convergence by using Tracer v1.7.1 (http://tree.bio.ed.ac.uk/software/tracer/).

## Genome Synteny and Collinearity Analysis

To compare the genome synteny of peafowl with chicken and turkey, the homologue of the genome was identified by using BLASTp (E-value < $1e^{-10}$). Gene pairs of synteny blocks within the genome were identified by using MCScanX [75], and the synteny blocks were showed by circos program from TBtools [76]. To estimate the positively selected genes for peafowl-chicken and peafowl-turkey, the value of Ka/Ks (ω) for each gene pair was calculated by KaKs_calculator [77], and the density curve of values was visualized by R software. The positively selected genes (ω > 1) were conducted based on functional enrichment analysis.

## Gene-family expansion and contraction

To identify the gene family expansion and contraction in peafowl, the gene families in 15 species and phylogenetic tree with divergent times were taken into account to estimate the significance of gene gain and loss in gene family by using the CAFE v4.2.1 with a random birth and death model and significance of P-values < 0.05 [78]. The parameter λ represents the probability of gene gain and loss in a divergent time. In order to investigate the evolutionary rates of different branches of the tree, the argument with "-t" was used to define three different branches for 15 species: the first branch included mouse and human, the second branch was the Phasianidaes, and other birds were regarded as the third branch. Then, they were conjunct with the "-s" option to search the optimal λ value for different branches by using the maximum likelihood.

## Positive Selection Analyses

To determine the adaptive evolution under the positive selection in peafowl, the single-copy orthologous protein sequences shared among the 11 species (peafowl, chicken, turkey, common mallard, zebra finch, collared flycatcher, medium ground-finch, tibetan ground-tit, rock pigeon, peregrine falcon and saker falcon) were searched, filtered, and then converted to coding gene sequence (CDS) by using EMBOSS backtranseq program [79], and the CDS were then aligned to codon by using PRANK with the option "-codon" [80]. The above alignments were analysed by using CODEML program of the PAML package 4.9 (Yang, 2007). A branch-site model (TEST-II) (model = 2, NSsites = 2) was conducted to identify the positively selected genes of peafowl. The model assumed that a particular branch (foreground, alternative hypothesis) had a different ω value from all the sites compared to all other branches (background, null hypothesis), suggesting that positive selection occurred at only a few

sites on a particular branch (foreground) [73]. The peafowl was regarded as a foreground branch and other species as a background branch. Additionally, the branch model was used to identify the rapidly evolving genes in peafowl, assuming that the branch of peafowl was as an alternative hypothesis (model = 2) and the branches of other species were as the null hypothesis (model = 0). The dN/dS (ω) values between foreground branch and background branch were estimated by using Likelihood Ratio Test (LRT) values based on chi-square test. When the ω value in the foreground branch was greater than that of the background branch, it suggested that the genes of the foreground branch were under positive selection ($P < 0.05$), and the positively selected sites were determined by using the Bayesian Empirical Bayes method. All the positively selected genes were performed in functional enrichment analysis by using KOBAS [81].

**Whole-genome resequencing and variant calling**

The genomic DNA from 35 blue feather peafowls and 16 white feather peafowls were pooled, respectively. Then 1.5 μg DNA per pool was used for constructing the sequencing libraries by using Truseq Nano DNA HT Sample preparation Kit (Illumina, USA) following manufacturer's constructions. Each pooled DNA was fragmented through sonication to a size of 350bp and end repaired, A-tailed, and ligated with the full-length adapter for Illumina sequencing with further PCR amplification. PCR-amplified sequencing libraries were purified (AMPure XP system) and analysed for size distribution on Agilent2100 Bioanalyzer, and were quantified by using real-time PCR. These libraries constructed above were sequenced on an Illumina NovaSeq platform and 150bp paired-end reads were generated with insert size around 350 bp. The raw data were filtered by removing reads with ≥10% unidentified nucleotides (N), reads with > 50% bases having phred quality < 5, and reads with > 10 nt aligned to the

adapter allowing ≤10% mismatches. The clean reads were mapped to the assembled reference genome by using BWA with parameters "mem -t 4 -k 32 –M –R". Alignment files were converted to BAM files by using SAMtools software (settings: –bS –t) [82]. In addition, potential PCR duplications were removed by using SAMtools command "rmdup". Single nucleotide polymorphisms (SNPs) and insertions/ deletions (Indels) (< 50 bp) were detected by using Genome Analysis Toolkit v 4.0 (GATK) pipeline [83].

**RNA sequencing (RNA-seq) on PacBio platform**

The cDNA of feather was acquired through PrimeScript™ RT reagent Kit with gDNA Eraser (TaKaRa Bio. Inc, Dalian, China) according to the manufacturer's instructions. The cDNA was performed damage repair, end repair, SMRT (single-molecule, real-time) dumbbell-shaped adapters, and ligation of the adapters to construct a mixed library. Primers and DNA polymerase were then combined to form a complete SMRT bell library. The qualified library was used for sequencing on a PacBio Sequel platform. The clean data were aligned to the reference genome of Indian peafowl by using STAR v2.5.3a [84]. The Transcript assembly and gene expression levels were conducted by using StringTie v1.3.3 (Pertea, et al., 2015) and featureCounts (Liao, et al., 2014) in Subread software [85]. Differentially expressed genes (DEGs) between blue and white feather were identified through DESeq2 (Love, et al., 2014) in condition of fold change >2 and P-value < 0.01. Subsequently, the functional enrichment analyses of DEGs were annotated through GO (Gene Ontology) [86] and KEGG (Kyoto Encyclopedia of Genes and Genomes) database.

**cDNA amplification**

cDNA of feathers was reversely transcribed with PrimeScript™ RT reagent Kit with gDNA Eraser (TaKaRa). The reverse transcription quantitative PCR (RT-qPCR) was conducted in a total volume of 10 µl including 5 µl SYBR Taq II kit (TaKaRa), 0.3 µl Rox Reference Dye (50x), 2.7 µl distilled water, 1 µl cDNA and 1 µl primers, and performed on a 7900HT RT-qPCR system (ABI). β-actin was selected as the internal reference gene. All primer sequences were presented in Supplementary Table S20.

# Results

## Genomic characteristics of Indian peafowl

The third-generation PacBio single-molecule real-time sequencing technology and the second-generation Illumina sequencing technology were used and combined with 10X genomics to assemble the Indian peafowl genome. The distribution of 17-kmer showed a major peak at 154× (Supplementary Figure S3). Based on the total number and corresponding k-mer depth of 154, the Indian peafowl genome size was estimated to 1.05 Gb. A total of two libraries were constructed with a sequencing volume of 164.03 Gb and a coverage depth of 154× performed on Illumina NovaSep 6000 platform. 10X Genomics sequencing platform yielded 112.57 Gb sequencing data with a coverage depth of 92×, and 110.74 Gb sequencing data was produced by using the PacBio sequencing platform with a coverage depth of 103× (Supplementary Table S1). In total, 387.34 Gb sequencing data and a total coverage 362× were obtained from the three sequencing strategies with the lengths of scaffold N50 and contigs N50 separately up to 6.2 Mb and 11.4 Mb, which exhibited 446-fold and 50-fold improvement in the scaffold N50 compared to previously published Indian blue peafowl genome reported by Jaiswal, et al. [11] and Dhar, et al. [12] (Figure 2, Table 1 and Supplementary Table S2). Current peafowl assembly anchored into 726 scaffolds with 1.05 Gb of sequences

and guanine-cytosine (GC) content was 42.03% with normally ratio of A, T, G and C (Figure 2 and Supplementary Table S2 - S3). Notably, the GC contents of scaffold 108 and scaffold 31 were more than 50%, and the gene density of them was relatively conferted (Figure 2). Moreover, it was apparently observed that the density of tandem repeat sequences in scaffold 39 was the higher than any other scaffolds (Figure 2).

To assess the completeness and base accuracy of the assembled Indian peafowl genome above, the short reads were aligned back to the draft genome with high mapping rate (98.05%), high coverage rate (99.87%) and low homozygous SNP rate (0.0002%), generally reflecting the accuracy of genome assembly (Supplementary Table S4-S5). The draft assembly was further evaluated by CEGMA, and the results showed that 88.71% of 248 core genes selected from 6 eukaryotic model organisms could be covered. Additionally, 97.4% complete genes (including 96.8% complete and single-copy genes and 0.6% complete and duplicated genes) were predicted, 1.7% fragmented genes and 0.9% missing genes were identified from 2,586 genes in Aves dataset by using the BUSCO (Supplementary Table S6). Collectively, these important indicators implied relatively high genome coverages and continuity for Indian blue peafowl genome, providing an important resource for molecular breeding and evolutionary studies for peafowl. The Indian peafowl genome with integrated annotations has been deposited in the Genome Warehouse in National Genomics Data Center, Beijing Institute of Genomics (BIG), Chinese Academy of Sciences, under accession number GWHAZTP00000000 publicly accessible at https://bigd.big.ac.cn/gwh [87].

Taking the Gallus gallus, Meleagris gallopavo, Peking duck, Struthio camelus, Nipponia nippon, and Eastern Zhejiang white goose genome as references, and

according to the homologous alignment and ab initio prediction, the peafowl genome comprised 15.20% non-redundant repeat sequences, including 1.27% tandem repeats, 14.12% transposable elements and 7.35% transposable element protein (Supplementary Table S7). A total of 14.56% of transposable elements was identified after combine TEs, 0.70% of which was DNA transposons, 3.93% was long terminal repeats (LTRs), 0.01% was short interspersed nuclear elements (SINE), and 10.68% was the long interspersed nuclear elements (LINE) (Supplementary Figure S4 and Supplementary Table S7-S8). Altogether, 19,465 non-redundant protein-coding genes were predicted, of which 15,766 (81%) were annotated to function by six public databases, including NR, Swissprot, KEGG, InterPro, GO and Pfam (Table 1 and Supplementary Table S9-S10). Additionally, 354 microRNAs (miRNAs), 308 transfer RNAs (tRNAs), 151 ribosomal RNAs (rRNAs) and 334 small nuclear RNAs (snRNAs) were also identified (Supplementary Table S11). Notably, compared with the other birds such as turkey, chicken, duck or previously reported draft peafowl genome, more non-redundant protein-coding genes and repetitive sequences as well as non-coding RNA in this draft peafowl genome were predicted. Overall, this assembly has more continuity, completeness, and accuracy, and the annotation of core eukaryotic genes and universal single-copy orthologs have also been improved.

**Gene families and phylogenetic relationship within the *Phasianidaes***

The protein sequences of 15 species were used to search the orthologues by using the OrthoFinder [88]. The results showed that a total of 18,038 orthogroups were identified

in 15 species, of which 5,999 single-copy orthologues were shared among these species (Figure 3a). In addition, 93 gene families were identified specific to peafowl and 11,447 gene families were shared by peafowl and other Phasianidaes (chicken, turkey and Japanese quail) (Figure 3b). The peafowl species-specific gene families were mainly involved in the immune response and biological process such as *FOXP3*, *FZD3*, and *TP53* participated in many immunological process and played an important role in melanoma and bone homeostasis (Supplementary Table S12) [89-91]. *FOXP3* is necessary for the development of regulatory T lymphocytes and is essential for maintaining the immune homeostasis and immune self-tolerance to the environmental antigens by eliminating natural reactive T cells in the thymus and peripheral organs. Meanwhile, *FOXP3* plays an important role in the bone and hematopoietic homeostasis, inflammatory bone loss diseases and abnormal bone weight, which can affect lymphoid hematopoiesis by acting on the development and function of osteoclasts [89]. *TP53* plays an important role in inhibiting the progression of bone and soft tissue sarcoma. The loss of *TP53* activity can promote the osteogenic differentiation of bone marrow stromal cells and the development of osteosarcoma of bone marrow mesenchymal stem cells, which can prevent the malignant transformation of bone marrow mesenchymal stem cells [90]. In this study, the enrichment of these genes specific to chicken and turkey in peafowl showed that the healthy development and immunity of bones were of great significance in peafowl evolution, which was conducive to achieving people's demand for rapid growth, large size, and strong disease resistance in domestication of peafowl.

To determine the phylogenetic relationship within the Phasianidae family, 5,999 single-copy orthologues were concatenated and aligned to construct phylogenetic tree

with a bootstrap value of 1,000 by using the maximum likelihood method (Supplementary Figure S5 and S6). The results showed that the Galliformes order were clustered, of which the Phasianidae family formed a group. Moreover, peafowl was found closer to turkey than chicken in the Phasianidae family that was inconsistent with Jaiswal, et al. reported [11], the relationship of chicken and quail was closer than turkey, and duck belonging to the Anseriformes order was close to the Galliformes order (Figure 3c). Additionally, the divergence time of all species was estimated and calibrated through the divergence time between human and mouse, human and zebra finch, zebra finch and medium ground finch, common mallard and zebra finch, and saker falcon and peregrine falcon from the TimeTree database. The divergence between Galliformes and Anseriformes was estimated to be 81.2 million years ago (Mya). The divergence between northern bobwhite and Phasianidae family was represented by the calibration point of northern bobwhite and turkey. The divergence between the peafowl and turkey was about 35.1 Mya, sharing a common ancestor with chicken about 36.9 Mya (Figure 3c). However, divergence between chicken and Japanese quail was estimated to be 34.7 Mya, within the range of divergence (33.2~42.3 Mya) from TimeTree [92], which suggested that the relationship between the common ancestor of peafowl and turkey, chicken and Japanese quail was very close to the relationship between these four species. The divergence time of pheasant birds is in the Tertiary era, which marks the advent of the modern biological era and is the heyday of the divergence of animals and plants. It presents that the new generation replaces the ancient types, with the increasing of the same species, common and diverse divergence of birds, and rapid evolution of more species during the Tertiary era.

**Genome synteny and collinearity among the Indian peafowl, chicken, and turkey**

Collinearity analysis can reflect the homology of different species and genetic relationship. Genes with the pairwise ratio of nonsynonymous to synonymous substitutions (dN/dS) could be used to infer the positive selection and contributed to understanding the evolutionary characteristics in species. In this study, the pairwise synteny was compared to peafowl and chicken, peafowl and turkey, and the ratio of dN/dS was calculated. The scaffolds of length greater than the scaffold N70 (5 Mb) in peafowl genome and other collinear scaffolds marked as others (Figure 4a and 4b) were displayed. Moreover, the distribution density of the dN/dS ratio was calculated and showed in Figure 4c. 97 positively selected genes (dN/dS > 1) in peafowl compared to chicken were associated with the biological process and immune-related pathways (*IL4*, *CD3D*, *CD3E* and *HLA-DMB*) ($P < 0.05$), such as, Th1 and Th2 cell differentiation, T cell receptor signaling pathway, and intestinal immune network for IgA production. Besides, compared with turkey, 43 positively selected genes were significantly enriched in the GO terms of organelle (GO:0043226), extracellular space (GO:0005615), and epithelium migration (GO:0090132), and the pathways of glutathione metabolism (*GPX1*, *GPX2* and *GPX4*) and thyroid hormone synthesis (*GPX1*, *DUOXA2* and *GPX2*) ($P < 0.05$) (Supplementary Table S13 and S14), which were involved in gastrointestinal health, anti-stress, growth development and metabolism. Notably, as a common positive selection gene, *EDN1* was reported to participate in many biological process, such as epithelium migration and differentiation, pigmentation and their receptors (EDNRs) widely distributed in various tissues in chicken [93]. These enrichment results indicated that the positively selected genes in peafowl were mainly related to intestinal immunity, anti-stress, growth development and metabolism, and pigmentation compared with turkey and chicken in the evolutionary process, which was beneficial

for peafowl to enhance adaptability, improve disease resistance and anti-stress ability, enrich plumage colour, and better adapt to the living environment of long-term artificial breeding.

**Gene family expansion and contraction across the Indian peafowl genome**

Likelihood analysis could identify the evolutionary rate and the significant expansion and contraction of gene family in species [78]. In this study, the changes of gene family in peafowl with likelihood ratio test were examined. Compared to the gene families in other species, the results suggested that 121 expansions and 2,999 contractions of gene families ($P < 0.05$) were detected in peafowl (Figure 3c), of which, 21 significantly gained genes were mainly involved in energy metabolism and storage (*GIMAP1*, *GIMAP2* and *GIMAP8*) and immune response (*CD244*) ($P < 0.05$), such as the GO terms of natural killer cell activation involved in immune response (GO:0002323), MHC class I protein binding (GO:0042288), positive regulation of interleukin-8 production (GO:0032757), positive regulation of interferon-gamma production (GO:0032729), and lipid droplet (GO:0005811) (Supplementary Table S15). Conversely, 23 significantly contracted genes were mainly relevant to the biological process such as fatty acid degradation (*ALDH3A2*) (GO:0001561), myocardium development (GO:0048739), muscle contraction and cardiac disease (*MYH6*, *MYH7*, and *MYH7b*), olfactory receptor activity (*OR52B2*, *OR52K1* and *OR4S1*) (GO:0004984), and the pathways of olfactory transduction, metabolism and cardiac muscle contraction (Supplementary Table S16). For example, the expression of *MYH6* and *MYH7* directly dictated the slow- or fast-twitch phenotype in skeletal muscle and played a vital role in cardiomyocyte energetics and metabolism [94, 95]. The olfactory genes were importantly characteristic during the adaptive evolution in birds [96].

Peafowl is artificially raised and feed-derived food as a result of the declining of finding food and flighting ability in the wild under the long-term domestication, which is likely to cause the contraction of genes related to the sense of smell and the regulation of skeletal muscle movement in peafowl. In addition, it was observed that the branch of Phasianidae had a higher rate of birth and death than that of other two branches, indicating that the family Phasianidae experienced a rapid evolution.

**Positively selected genes in the Indian peafowl genome**

To reveal the adaptive divergence and evolution of peafowl, positive selection was analysed by using the branch-site model in CODEML program. The significantly positive sites were evaluated by the Bayes Empirical Bayes values (BEB ≥ 0.95), which demonstrated that the sites were under positive selection in branch-site model A (foreground). In the branch of peafowl (foreground), 3,417 genes were under significantly positive selection based on the Bayes Empirical Bayes values ($P < 0.05$). These genes were annotated and classified through the analysis of GO ontology and KEGG pathways in order to further explore the impact of adaptive evolution on peafowl. According to the results of functional enrichment analyses, it was briefly summarized that these positively selective genes were mainly participated in the process of lipid metabolism (i.e., GO:0005811, GO:0030169, and GO:0008289), limb and skeletal development (i.e., GO:0060173, GO:0001503, and GO:0030509), immune response (i.e., GO:0070498, GO:0043123, and GO:1901224), pigmentation (GO:0042470 and GO:0030318), sensory perception (i.e., GO:0008542, GO:0008542, and GO:0007605), and other GO terms (Supplementary Table S17). Additionally, the pathways of positively selected genes were significantly enriched in metabolic pathways, PI3K-Akt signaling pathway, NF-kappa B signaling pathway, pathways in cancer, MAPK

signaling pathway, TNF signaling pathway, Jak-STAT signaling pathway, mTOR signaling pathway, FoxO signaling pathway, fatty acid metabolism, IL-17 signaling pathway, cholesterol metabolism, Th17 cell differentiation, and so on (Supplementary Table S18), which were mainly associated with immunity, energy metabolism, and cell growth and differentiation.

Furthermore, the branch model was used to identify a total of 10 rapidly evolving genes in peafowl, including *BCl7A*, *MEF2C*, *MED27*, *COPS7A*, *NMNAT2*, *SLC25A25*, *TNIP2*, *ETS1*, *CCDC6* and *GSG1L*. Functional enrichment showed that the significantly pathways included pathways in cancer, nicotinate and nicotinamide metabolism, thyroid cancer, renal cell carcinoma, parathyroid hormone synthesis, secretion and action, thyroid hormone signaling pathway, apelin signaling pathway, fluid shear stress and atherosclerosis ($P < 0.05$). The significant GO terms were involved in melanocyte differentiation, skeletal muscle and bone development, immunity, and response to stress (Supplementary Table S19). Particularly, it was observed that *MEF2C* participated in most of GO terms and pathways, which might have important implications in the rapid evolution of peafowl. Cartilage formation was a key process in vertebrate bone development and health maintenance that most bones were developed through cartilage ossification. *MEF2C* could regulate muscle and cardiovascular development, and was not only a core component of development in regulating muscle, nerve, cartilage-like, immune and endothelial cells, but also necessary for normal chondrocyte hypertrophy and ossification [97, 98]. Potthoff, et al. suggested that *MEF2C* could directly regulate the transcription of myosin gene, and the loss of *MEF2C* in skeletal muscle caused improper sarcomere organization, which revealed the key role of *MEF2C* in maintaining sarcomere integrity and skeletal muscle

maturation after birth [99]. Arnold, et al. indicated that the transcription factor *MEF2C* could regulate muscle and cardiovascular development, and control skeletal development by activating the genetic program of chondrocyte hypertrophy [100]. In this study, it was observed that *MEF2C* underwent rapid evolution in peafowl, which was conducive to the development and morphology of bones and the maintenance of body shape of peafowl. This may be due to the increase in weight and body size of peafowl in order to meet people's needs during the domestication process, and could be well explain the evolutionary phenotype characteristics of peafowl.

**Genes with allele frequency between blue and white feather Indian peafowl**

To localize the genomic region underlying the plumage colour, the allele frequency between blue and white feather peafowl was analysed. The clean data of two pooled resequencing were aligned to the assembled peafowl genome by using the Samtools with option "mpileup", and filtered to calculate allele frequency differences by using Population2 software [101]. The significance of allele frequency differences was estimated by Fisher's exact test. The up- and downstream of 50 Kb with -log10 (P-value) greater than 30 as potential candidate regions was extracted . As a result, it was found that *EDNRB* in scaffold 196 and *PMEL* in scaffold 144 were significantly related to plumage pigmentation (Figure 5a). Additionally, 69 down-regulated genes and 52 up-regulated genes between blue and white peafowl were detected, of which ten up-regulated genes (*TRYP1*, *TYR*, *PMEL*, *EDNRB*, *OCA2*, *SLC24A5*, *SOX10*, *MC1R*, *SLC45A2* and *TRPM1*) were associated with melanin deposition (Figure 5b). The functional enrichment of DEGs showed the most significant pathway was enriched in the process of melanin synthesis based on the criterion of $P < 0.05$ as significant (Figure 5c). In order to further investigate the allele imbalance difference sites of DEGs, the

allele frequency of ten pigmentation-related genes in the blue and white peafowl was calculated, and the function of sites was annotated by using the snpEff software [102]. An observation showed that two differential sites were located in *PMEL* and one in *EDNRB*, but none of the differential sites were obviously functional mutations, such as missense mutations, splicing mutations, or nonsense mutations (Figure 5d). These results suggested that the formation of white feathers was most likely related to the differential expression of *PMEL* and *EDNRB* in peafowl.

**Candidate causative gene for the phenotype of white feather in blue and white feather peafowl**

To detect the *PMEL* and *EDNRB* transcripts in blue and white feather peafowl, the RNA-seq data of *PMEL* and *EDNRB* were examined by using the integrative genomics viewer (IGV) application, and it was observed no difference in the transcript of *EDNRB* in the two types of feather pulp (Supplementary Figure S7), suggesting that *ENDRB* was normally expressed in blue and white feather peafowl. When compared the transcript of *PMEL* in blue and white feather peafowl, it was found that *PMEL* was almost not expressed in white feather peafowl (Figure 5e). To further determine the mRNA expression of *PMEL* in white feather peafowl, reverse transcription quantitative PCR (RT-qPCR) of *PMEL* was conducted in blue and white feather peafowl (Supplementary Table S20). RNA samples were extracted from feather pulps and used for subsequent PCR. Surprisingly, it was observed that the mRNA expression of *PMEL* in white feather peafowl significantly reduced in comparison to that in the blue feather peafowl ($P = 0.013$) (Figure 5f), which was consistent with the result of RNA-seq data. Hence, it was argued that *PMEL* was a strong candidate causative gene for the formation of white feathers in blue and white feather peafowl. Further investigations

are needed to conduct on the mechanism for the downregulated expression of *PMEL* in white feather peafowl.

## Discussion

With the development of sequencing technology, the reduction of sequencing costs and the improvement of assembly methods, the increasing genome sequence maps of various species have been published, making the whole genome sequencing to be an important method for conducting basic genetic research on species. Recently, many avian genomes have been assembled, providing good materials to study the genetic mechanisms of evolution, behaviour and pathology. In this study, three sequencing strategies were combined to construct the India peafowl genome, and totally 1.05 Gb draft genome sequence was obtained, and even the sequencing depth was up to 362×. Moreover, the lengths of contigs N50 and scaffold N50 were respectively achieved to 6.2 Mb and 11.4 Mb, which was closer to the chromosomal level. Compared with other avian genomes and the draft genomes of peafowl assembled by Jaiswal, et al. and Dhar, et al. [11, 12], the Indian blue peafowl genome in this study showed a significantly improved assembly quality, including consistency, accuracy and integrality. This draft genome of peafowl was a considerable improvement in terms of the quality of genome assembly and provided a strong support and guarantee for the subsequent comparative genomic analysis.

The phylogeny of birds has always been a matter of great concern and controversy. Generally, researches on phylogenetic relationship of birds are much more based on mitochondrial DNA, cytochrome b gene, nuclear genes or a combination of them [103-105]. Jaiswal's study demonstrated that the phylogenetic trees of peafowl constructed

by the data of nuclear intron regions and mitochondrial genomes supported that peafowl and chicken were clustered and closer than turkey [106]. Whereas, the phylogenetic tree based on retroposon insertion and the chromosomal showed that peafowl was closer to turkey than to chicken [107]. Similarly, Kaiser's research suggested that the result by using genome-based retrotransposon data to construct the taxonomic status of Phasianidae including chicken, turkey, Japanese quail and peafowl was consistent with Shibusawa's study, but was disaccord with the observation based on nucleotide sequence that the relationship of chicken and turkey was closer than peafowl [108]. Many studies based on different data types to construct the tree proved that it was uncertain and controversial in the phylogenetic classification of Phasianidae families and may need to search for more evidence to verify. In this study, the single copy homologous amino acid sequence from the whole genome sequencing data was used to construct phylogenetic tree, and it was observed that the position of peafowl was closer to turkey than to chicken, which was disagreed with previous studies [11, 12]. In addition, according to the divergent time, it was estimated that the divergence between peafowl and turkey was near to the divergence time among chicken and the ancestors of peafowl and turkey, which indicated that the relationship among them was very close, and inconsistent phylogenetic trees were constructed under different strategies.

In recent years, since the rapid development of genomics and the accumulation of genomic data, comparative genomics has become a research hotspot, which could explain biological functions and evolutionary characteristics at the genome-wide level. Especially, avian genomes were favour of people to investigate the adaptive evolution

and species-specific biological characteristics by discovering novel genes and gene function through comparative genomics analysis. In this study, comparative genomics analysis was conducted on peafowls and other birds to explore the uniquely biological characteristics of peafowl during the evolution.

Species-specific immune-related genes were always positively selected in the adaptive evolution in many species. In this study, the number of GO terms and pathways related to immunity in peafowl was more than that of others, such as the expansive genes and rapidly evolving genes involved in the process of MHC class I protein binding, TNF signaling pathway, NF-kappaB signaling, IL-17 signaling pathway and Th17 cell differentiation. Likewise, it was observed that many olfactory genes and myosin genes were lost in peafowl. Mysosin was a functional protein and structural protein, which directly regulated muscle contraction, movement and cardiac functions in animals [109]. Olfaction performed a crucial role in life of birds, which contributed to recognise foods, make courtship, or detect danger [110, 111]. It was reported that birds could recognize close relatives to avoid inbreeding and distinguish direction in migration by using their acute sense of olfaction [112, 113]. However, in this study, peafowl was artificial farmed and the feed-derived food was sufficient, causing the gradual degradation of the ability to find food in the wild and fly, which may explain the loss of myosin family and olfactory family and contribute to inbreeding and reduction of energy expenditure.

Generally, most of the body size of birds was small due to the pressure of body weight and reduction in energy expenditure [114]. However, the peafowl is known to have large body size, huge tail, and beautiful plumage, which is likely to be gradually evolved due to better adaption to ecological environment. In this study, the enrichment

analysis of positive selection genes was mainly involved in skeletal development, bone morphology, energy metabolism and storage, such as the mTOR signaling pathway, MAPK signaling pathway, BMP signaling pathway, limb development, lipid droplet, and lipid binding. mTOR was a central integrator of cellular growth and metabolism, and the mTOR signaling pathway played a vital role in innate and adaptive immune responses and regulating energy balance [115, 116]. BMP was an important member of the transforming growth factor-β (TGF-β) superfamily through regulating the activity of downstream genes to participate in many important biological processes, such as nervous system differentiation, tooth and bone development, and cancer [117, 118]. MAPK signalling pathway also participated in the regulation of feather growth and development [119]. Moreover, as the rapid evolving genes, *MEF2C* exhibited many functions such as bone and muscle development, immune, and melanocyte differentiation [120-122]. Furthermore, the glittering plumage and long tail also deeply attracted people's attention. Many positively selected genes associated with pigmentation like *TYR*, *SZT2*, *NF1*, *ARCN1*, *KIT*, *HPS5*, *FIG4*, *LYST*, *RACK1*, *USP13*, *Hps6*, *OCA2*, *MITF* and *BCL2* were also identified. All of the above results could be well contributed to understanding the phenotypic characteristics, such as large body size, long tail, and dazzling plumage in peafowl during the adaptive evolutionary process.

To date, a number of researches about the genetic mechanism of plumage colour in avian have been reported [123, 124]. In the present study, the mechanism of the formation of white plumage phenotype was explored in peafowl combined transcriptome analysis and RT-qPCR with resequencing data. As for the result of allele frequency difference, the *PMEL* and *EDNRB* related to melanin were screened.

Meanwhile, it was detected that the *PMEL* and *EDNRB* were contained in the DEGs from the transcriptome analysis, and it was found that the enrichment of DEGs was also involved in melanin biosynthetic process, which was in accordance with the result of allele frequency difference. Then, it was identified that the *PMEL* and *EDNRB* were the candidate genes of white plumage through the results of the allele frequency difference of DEGs sites. Combined with the difference of the *PMEL* and *EDNRB* transcript by the results of IGV visualization and RT-qPCR, it was confirmed that *PMEL* was the causative gene of white plumage in blue and white feather peafowl. The formation and deposition of melanin mainly occurred on amyloid fibres of melanosomes. As a key signal molecule, *PMEL* could directly initiate the formation of melanosomes and promote the synthesis of melanosomes [125]. Moreover, it was reported in many studies that mutations of *PMEL* could cause low expression of *PMEL* leading to melanogenesis, further resulting in hypopigmentation phenotypes in animals like silver horse, white chicken and yellowish Japanese quail [126-128]. In this study, it was also detected that the low expression of *PMEL* was associated with the white plumage in peafowl. However, in order to further investigate the causal mutations of *PMEL* low-expressed, the mutations of *PMEL* were examined and annotated, but not any functional mutation sites was found. It was supposed that the low expression of *PMEL* transcript was probably caused by the changes of the regulatory elements located in the upstream 5 kb promoter region of *PMEL*, and then the melanin synthesis was impeded. Unfortunately, there were no mutations in the core promoter region and transcription factor binding sites predicted by promoter prediction websites. In addition, the resequencing data were also used to detect the structural variation of the *PMEL* gene and its upstream, and the transcriptome data were used to detect the SNP and Indel variation, as well as PCR

amplification of the *PMEL* gene and its upstream 5 kb promoter region for Sanger sequencing, but none possible variations was found. In view of this, it was speculated that the *PMEL* gene was likely to exist as a complex structure so that it couldn't be completely measured through sequencing and the causal sites weren't identified, which was needed to be further explored. Nevertheless, it was firstly identified that *PMEL* was a causal gene of white plumage, providing a novel insight into the formation of white feather phenotype in blue and white feather peafowl. The results revealed the genetic mechanism of white plumage at the whole-genome transcriptome level.

## Conculsion

In conclusion, for the first time, the study accomplished the assembly of a higher quality and greater sequencing depth of peafowl genome closer to the chromosomal level. Firstly, the assembly genome is superior to other two draft genomes of peafowl either in terms of the sequencing depth or the assembly quality. Secondly, the study determined the phylogenetic relationship of peafowl among the Phasianidae, and found that peafowl was closer to turkey than to chicken by the analysis of single-copy orthologues genes based on the above assembly draft genome. Moreover, the comparative genome analysis indicated that Indian peafowl evolved on metabolism, immunity, skeletal development and feather development may approximatively be related to the unique characteristics of peafowl in domestication, which was conducted to provide baseline information about the phenotypic evolution of peafowl. Finally, the study was the first report to perform a combination of resequencing and transcriptome analysis in Indian peafowl, and revealed the molecular mechanism of white plumage formation in peafowl. Altogether, this study work provided a novel reference genome in the systematic evolution of peafowl and other birds, which was helpful to understand

the formation of plumage coloration in peafowl and other colorful birds and provided a new idea for artificial breeding in peafowl.

## Data availability

The whole genome sequence data reported in this paper have been deposited in the Genome Warehouse in National Genomics Data Center [87], Beijing Institute of Genomics (BIG), Chinese Academy of Sciences, under accession number GWHAZTP00000000 that is publicly accessible at https://bigd.big.ac.cn/gwh. The resequencing raw data has been deposited in the NCBI Sequence Read Archive (SRA) (https://submit.ncbi.nlm.nih.gov/subs/sra/) under accession number PRJNA665082. The transcriptomic raw data has been deposited in the NCBI under accession number PRJNA661158.

## Author Contributions

X.Y., H.M. and J.R. designed the study and wrote the paper. S.L. and H.C. analyzed the data and wrote the paper. S.L., H.C., H.M. and W.L. revised the paper. S.L., H.Z. and B.L. conducted the validated experiments. S.L., J.O., M.H., S.Z., S.X., H.T., Y.G., Y.X., D.C., K.C., H.M. and Y.X. collected samples and performed the sequencing and genotyping experiments. All authors contributed and approved the final manuscript.

## Funding

This work was supported by Educational Commission of Jiangxi Province of China (No. GJJ190177) and by the Key Research and Development Program of Jiangxi Province of China (No. 20171BBF60003).

## Ethics approval and consent to participate

All procedures used for this study and involved in animals fully complied with guidelines for the care and utility of experimental animals established by the Ministry of Agriculture of China. The Animal Care and Use Committee of the South China Agricultural University approved this study.

## Competing financial interests

The authors declare that they have no competing financial interests.

## References


1. Gadagkar R: Is the peacock merely beautiful or also honest? Current Science 2003, 85(7):1012-1020.
2. Kushwaha S, Kumar AJJWR: A review on Indian peafowl (Pavo cristatus) Linnaeus, 1758. 2016, 4(4):42-59.
3. Ramesh K, McGowan P: On the current status of Indian Peafowl Pavo cristatus (Aves: Galliformes: Phasianidae): keeping the common species common. Journal of Threatened Taxa 2009, 1:106-108.
4. Talha MMH, Mia MM, Momu JMJIJoDR: Morphometric, productive and reproductive traits of Indian peafowl (Pavo cristatus) in Bangladesh. 2018, 8(02):19039-19043.
5. Paranjpe D, Dange PJb: A tale of two species: human and peafowl interactions in human dominated landscape influence each others behaviour. 2019:412254.
6. Mushtaq-ul-Hassan M, Ali Z, Arshad MI, Mahmood S, Research MM-u-HJIJoV: Effects of mating sex ratios in Indian peafowl (Pavo cristatus) on production

performance at Wildlife Research Institute, Faisalabad (Pakistan). 2012, 13(2):143-146.

7. Sequence and comparative analysis of the chicken genome provide unique perspectives on vertebrate evolution. Nature 2004, 432(7018):695-716.

8. Dalloul RA, Long JA, Zimin AV, Aslam L, Beal K, Blomberg Le A, Bouffard P, Burt DW, Crasta O, Crooijmans RP et al: Multi-platform next-generation sequencing of the domestic turkey (Meleagris gallopavo): genome assembly and analysis. PLoS biology 2010, 8(9).

9. Zhang Z, Jia Y, Almeida P, Mank JE, van Tuinen M, Wang Q, Jiang Z, Chen Y, Zhan K, Hou S et al: Whole-genome resequencing reveals signatures of selection and timing of duck domestication. Gigascience 2018, 7(4).

10. Jarvis ED, Mirarab S, Aberer AJ, Li B, Houde P, Li C, Ho SY, Faircloth BC, Nabholz B, Howard JT et al: Whole-genome analyses resolve early branches in the tree of life of modern birds. Science 2014, 346(6215):1320-1331.

11. Jaiswal SK, Gupta A, Saxena R, Prasoodanan VPK, Sharma AK, Mittal P, Roy A, Shafer ABA, Vijay N, Sharma VK: Genome Sequence of Peacock Reveals the Peculiar Case of a Glittering Bird. Front Genet 2018, 9:392-392.

12. Dhar R, Seethy A, Pethusamy K, Singh S, Rohil V, Purkayastha K, Mukherjee I, Goswami S, Singh R, Raj A et al: De novo assembly of the Indian blue peacock (Pavo cristatus) genome using Oxford Nanopore technology and Illumina sequencing. Gigascience 2019, 8(5):giz038.13. Dakin R, McCrossan O, Hare JF, Montgomerie R,

Amador Kane S: Biomechanics of the Peacock's Display: How Feather Structure and Resonance Influence Multimodal Signaling. PloS one 2016, 11(4):e0152759.

14. Wang Y, Zhao H, Liu J, Shao Y, Xing M: Molecular cloning and transcriptional regulation of Indian peafowl (Pavo cristatus) IFN-α gene. Cell stress & chaperones 2019, 24(2):323-332.

15. Samour J, Naldo J, Rahman H, Sakkir M: Hematologic and plasma biochemical reference values in Indian peafowl (Pavo cristatus). Journal of avian medicine and surgery 2010, 24(2):99-106.

16. Zhou TC, Sha T, Irwin DM, Zhang YP: Complete mitochondrial genome of the Indian peafowl (Pavo cristatus), with phylogenetic analysis in phasianidae. Mitochondrial DNA 2015, 26(6):912-913.

17. Shen YY, Dai K, Cao X, Murphy RW, Shen XJ, Zhang YP: The updated phylogenies of the phasianidae based on combined data of nuclear and mitochondrial DNA. PloS one 2014, 9(4):e95786.

18. Naseer J, Anjum K, Khan W, Imran M, Ishaque M, Hafeez S, Munir MA, Nazir MA: Phylogenetic analysis based studies on genetic variation of cytochrome B gene of Indian peafowl (Pavo cristatus) in Pakistan. Indian Journal of Animal Research 2018, 52:343-346.

19. Bush KL, Strobeck C: Phylogenetic relationships of the phasianidae reveals possible non-pheasant taxa. The Journal of heredity 2003, 94(6):472-489.

20. Chang J, Wang B, Zhang YY, Liu Y, Liang W, Wang JC, Shi HT, Su WB, Zhang ZW: Molecular evidence for species status of the endangered Hainan peacock pheasant. Zoological science 2008, 25(1):30-35.

21. Sun K, Meiklejohn KA, Faircloth BC, Glenn TC, Braun EL, Kimball RT: The evolution of peafowl and other taxa with ocelli (eyespots): a phylogenomic approach. Proceedings Biological sciences 2014, 281(1790).

22. Ouyang YN, Yang ZY, Da-Lin LI, Huo JL, Qian K, Miao YWJJoYAU: Genetic Divergence between Pavo muticus and Pavo cristatus by Cyt b Gene. 2009.

23. Somes RG, Burger RE: Inheritance of the White and Pied Plumage Color Patterns in the Indian Peafowl (Pavo cristatus). 1993, 14(1):53-55.

24. Somes RG, Burger REJJoH: Plumage Color Inheritance of the Indian Blue Peafowl (Pavo Cristatus): Blue, Black-Shouldered, Cameo, and Oaten. 1991(1):1.

25. Chen Y, Ye W, Zhang Y, Xu Y: High speed BLASTN: an accelerated MegaBLAST search tool. Nucleic acids research 2015, 43(16):7762-7768.

26. Chin C-S, Peluso P, Sedlazeck FJ, Nattestad M, Concepcion GT, Clum A, Dunn C, O'Malley R, Figueroa-Balderas R, Morales-Cruz AJNm: Phased diploid genome assembly with single-molecule real-time sequencing. 2016, 13(12):1050-1054.

27. Chin CS, Alexander DH, Marks P, Klammer AA, Drake J, Heiner C, Clum A, Copeland A, Huddleston J, Eichler EE et al: Nonhybrid, finished microbial genome assemblies from long-read SMRT sequencing data. Nature methods 2013, 10(6):563-569.

28. Walker BJ, Abeel T, Shea T, Priest M, Abouelliel A, Sakthikumar S, Cuomo CA, Zeng Q, Wortman J, Young SK et al: Pilon: an integrated tool for comprehensive microbial variant detection and genome assembly improvement. PloS one 2014, 9(11):e112963.

29. Adey A, Kitzman JO, Burton JN, Daza R, Kumar A, Christiansen L, Ronaghi M, Amini S, Gunderson KL, Steemers FJ et al: In vitro, long-range sequence information for de novo genome assembly via transposase contiguity. Genome research 2014, 24(12):2041-2049.

30. Li H, Durbin R: Fast and accurate short read alignment with Burrows-Wheeler transform. Bioinformatics 2009, 25(14):1754-1760.

31. Simão FA, Waterhouse RM, Ioannidis P, Kriventseva EV, Zdobnov EM: BUSCO: assessing genome assembly and annotation completeness with single-copy orthologs. Bioinformatics 2015, 31(19):3210-3212.

32. Parra G, Bradnam K, Korf I: CEGMA: a pipeline to accurately annotate core genes in eukaryotic genomes. Bioinformatics 2007, 23(9):1061-1067.

33. Parra G, Bradnam K, Ning Z, Keane T, Korf I: Assessing the gene space in draft genomes. Nucleic acids research 2009, 37(1):289-297.

34. Tempel S: Using and understanding RepeatMasker. Methods in molecular biology (Clifton, NJ) 2012, 859:29-51.

35. Jurka J, Kapitonov VV, Pavlicek A, Klonowski P, Kohany O, Walichiewicz J: Repbase Update, a database of eukaryotic repetitive elements. Cytogenetic and genome research 2005, 110(1-4):462-467.

36. Xu Z, Wang H: LTR_FINDER: an efficient tool for the prediction of full-length LTR retrotransposons. Nucleic acids research 2007, 35(Web Server issue):W265-268.

37. Price AL, Jones NC, Pevzner PA: De novo identification of repeat families in large genomes. Bioinformatics 2005, 21 Suppl 1:i351-358.

38. Flynn JM, Hubley R, Goubert C, Rosen J, Clark AG, Feschotte C, Smit AF: RepeatModeler2 for automated genomic discovery of transposable element families. Proceedings of the National Academy of Sciences of the United States of America 2020, 117(17):9451-9457.

39. Benson G: Tandem repeats finder: a program to analyze DNA sequences. Nucleic acids research 1999, 27(2):573-580.

40. Kent WJ: BLAT--the BLAST-like alignment tool. Genome research 2002, 12(4):656-664.

41. Birney E, Clamp M, Durbin R: GeneWise and Genomewise. Genome research 2004, 14(5):988-995.

42. Stanke M, Keller O, Gunduz I, Hayes A, Waack S, Morgenstern B: AUGUSTUS: ab initio prediction of alternative transcripts. Nucleic acids research 2006, 34(Web Server issue):W435-439.

43. Majoros WH, Pertea M, Salzberg SL: TigrScan and GlimmerHMM: two open source ab initio eukaryotic gene-finders. Bioinformatics 2004, 20(16):2878-2879.

44. Alioto T, Blanco E, Parra G, Guigó R: Using geneid to Identify Genes. Current protocols in bioinformatics 2018, 64(1):e56.

45. Burge C, Karlin S: Prediction of complete gene structures in human genomic DNA. Journal of molecular biology 1997, 268(1):78-94.

46. Korf I: Gene finding in novel genomes. BMC bioinformatics 2004, 5:59.

47. Haas BJ, Salzberg SL, Zhu W, Pertea M, Allen JE, Orvis J, White O, Buell CR, Wortman JR: Automated eukaryotic gene structure annotation using EVidenceModeler and the Program to Assemble Spliced Alignments. Genome Biol 2008, 9(1):R7.

48. Bairoch A, Apweiler R: The SWISS-PROT protein sequence database and its supplement TrEMBL in 2000. Nucleic acids research 2000, 28(1):45-48.

49. O'Leary NA, Wright MW, Brister JR, Ciufo S, Haddad D, McVeigh R, Rajput B, Robbertse B, Smith-White B, Ako-Adjei D et al: Reference sequence (RefSeq) database at NCBI: current status, taxonomic expansion, and functional annotation. Nucleic acids research 2016, 44(D1):D733-745.

50. El-Gebali S, Mistry J, Bateman A, Eddy SR, Luciani A, Potter SC, Qureshi M, Richardson LJ, Salazar GA, Smart A et al: The Pfam protein families database in 2019. Nucleic acids research 2019, 47(D1):D427-d432.

51. Kanehisa M, Goto S: KEGG: kyoto encyclopedia of genes and genomes. Nucleic acids research 2000, 28(1):27-30.

52. Zdobnov EM, Apweiler R: InterProScan--an integration platform for the signature-recognition methods in InterPro. Bioinformatics 2001, 17(9):847-848.

53. Lowe TM, Eddy SR: tRNAscan-SE: a program for improved detection of transfer RNA genes in genomic sequence. Nucleic acids research 1997, 25(5):955-964.

54. Nawrocki EP, Eddy SR: Infernal 1.1: 100-fold faster RNA homology searches. Bioinformatics 2013, 29(22):2933-2935.

55. Griffiths-Jones S, Moxon S, Marshall M, Khanna A, Eddy SR, Bateman A: Rfam: annotating non-coding RNAs in complete genomes. Nucleic acids research 2005, 33(Database issue):D121-124.

56. Nishibori M, Hayashi T, Tsudzuki M, Yamamoto Y, Yasue H: Complete sequence of the Japanese quail (Coturnix japonica) mitochondrial genome and its genetic relationship with related species. Animal genetics 2001, 32(6):380-385.

57. Bellott DW, Skaletsky H, Cho TJ, Brown L, Locke D, Chen N, Galkina S, Pyntikova T, Koutseva N, Graves T et al: Avian W and mammalian Y chromosomes convergently retained dosage-sensitive regulators. Nat Genet 2017, 49(3):387-394.

58. Oldeschulte DL, Halley YA, Wilson ML, Bhattarai EK, Brashear W, Hill J, Metz RP, Johnson CD, Rollins D, Peterson MJ et al: Annotated Draft Genome Assemblies for the Northern Bobwhite (Colinus virginianus) and the Scaled Quail (Callipepla squamata) Reveal Disparate Estimates of Modern Genome Diversity and Historic Effective Population Size. G3 (Bethesda, Md) 2017, 7(9):3047-3058.

59. Gregory MK, James MJ: Functional characterization of the duck and turkey fatty acyl elongase enzymes ELOVL5 and ELOVL2. The Journal of nutrition 2014, 144(8):1234-1239.

60. Korlach J, Gedman G, Kingan SB, Chin CS, Howard JT, Audet JN, Cantin L, Jarvis ED: De novo PacBio long-read and phased avian genome assemblies correct and add

to reference genes generated with intermediate and short reads. Gigascience 2017, 6(10):1-16.

61. Ellegren H, Smeds L, Burri R, Olason PI, Backström N, Kawakami T, Künstner A, Mäkinen H, Nadachowska-Brzyska K, Qvarnström A et al: The genomic landscape of species divergence in Ficedula flycatchers. Nature 2012, 491(7426):756-760.

62. Zhang G, Li C, Li Q, Li B, Larkin DM, Lee C, Storz JF, Antunes A, Greenwold MJ, Meredith RW et al: Comparative genomics reveals insights into avian genome evolution and adaptation. Science 2014, 346(6215):1311-1320.

63. Cai Q, Qian X, Lang Y, Luo Y, Xu J, Pan S, Hui Y, Gou C, Cai Y, Hao M et al: Genome sequence of ground tit Pseudopodoces humilis and its adaptation to high altitude. Genome Biol 2013, 14(3):R29.

64. Shapiro MD, Kronenberg Z, Li C, Domyan ET, Pan H, Campbell M, Tan H, Huff CD, Hu H, Vickrey AI et al: Genomic diversity and evolution of the head crest in the rock pigeon. Science 2013, 339(6123):1063-1067.

65. Zhan X, Pan S, Wang J, Dixon A, He J, Muller MG, Ni P, Hu L, Liu Y, Hou H et al: Peregrine and saker falcon genome sequences provide insights into evolution of a predatory lifestyle. Nat Genet 2013, 45(5):563-566.

66. Friedman-Einat M, Cogburn LA, Yosefi S, Hen G, Shinder D, Shirak A, Seroussi E: Discovery and characterization of the first genuine avian leptin gene in the rock dove (Columba livia). Endocrinology 2014, 155(9):3376-3384.

67. Mohajeri K, Cantsilieris S, Huddleston J, Nelson BJ, Coe BP, Campbell CD, Baker C, Harshman L, Munson KM, Kronenberg ZN et al: Interchromosomal core duplicons drive both evolutionary instability and disease susceptibility of the Chromosome 8p23.1 region. Genome research 2016, 26(11):1453-1467.

68. Church DM, Schneider VA, Graves T, Auger K, Cunningham F, Bouk N, Chen HC, Agarwala R, McLaren WM, Ritchie GR et al: Modernizing reference genome assemblies. PLoS biology 2011, 9(7):e1001091.

69. Yu C, Zavaljevski N, Desai V, Reifman J: QuartetS: a fast and accurate algorithm for large-scale orthology detection. Nucleic acids research 2011, 39(13):e88.

70. Katoh K, Standley DM: MAFFT multiple sequence alignment software version 7: improvements in performance and usability. Molecular biology and evolution 2013, 30(4):772-780.

71. Capella-Gutiérrez S, Silla-Martínez JM, Gabaldón T: trimAl: a tool for automated alignment trimming in large-scale phylogenetic analyses. Bioinformatics 2009, 25(15):1972-1973.

72. Minh BQ, Schmidt HA, Chernomor O, Schrempf D, Woodhams MD, von Haeseler A, Lanfear R: IQ-TREE 2: New Models and Efficient Methods for Phylogenetic Inference in the Genomic Era. Molecular biology and evolution 2020, 37(5):1530-1534.

73. Yang Z: PAML 4: phylogenetic analysis by maximum likelihood. Molecular biology and evolution 2007, 24(8):1586-1591.

74. Hedges SB, Dudley J, Kumar S: TimeTree: a public knowledge-base of divergence times among organisms. Bioinformatics 2006, 22(23):2971-2972.

75. Wang Y, Tang H, Debarry JD, Tan X, Li J, Wang X, Lee TH, Jin H, Marler B, Guo H et al: MCScanX: a toolkit for detection and evolutionary analysis of gene synteny and collinearity. Nucleic acids research 2012, 40(7):e49.

76. Chen C, Chen H, Zhang Y, Thomas HR, Frank MH, He Y, Xia R: TBtools: An Integrative Toolkit Developed for Interactive Analyses of Big Biological Data. Molecular plant 2020, 13(8):1194-1202.

77. Zhang Z, Li J, Zhao XQ, Wang J, Wong GK, Yu J: KaKs_Calculator: calculating Ka and Ks through model selection and model averaging. Genomics, proteomics & bioinformatics 2006, 4(4):259-263.

78. De Bie T, Cristianini N, Demuth JP, Hahn MW: CAFE: a computational tool for the study of gene family evolution. Bioinformatics 2006, 22(10):1269-1271.

79. Rice P, Longden I, Bleasby A: EMBOSS: the European Molecular Biology Open Software Suite. Trends in genetics : TIG 2000, 16(6):276-277.

80. Löytynoja A: Phylogeny-aware alignment with PRANK. Methods in molecular biology (Clifton, NJ) 2014, 1079:155-170.

81. Xie C, Mao X, Huang J, Ding Y, Wu J, Dong S, Kong L, Gao G, Li CY, Wei L: KOBAS 2.0: a web server for annotation and identification of enriched pathways and diseases. Nucleic acids research 2011, 39(Web Server issue):W316-322.

82. Li H, Handsaker B, Wysoker A, Fennell T, Ruan J, Homer N, Marth G, Abecasis G, Durbin R: The Sequence Alignment/Map format and SAMtools. Bioinformatics 2009, 25(16):2078-2079.

83. McKenna A, Hanna M, Banks E, Sivachenko A, Cibulskis K, Kernytsky A, Garimella K, Altshuler D, Gabriel S, Daly M et al: The Genome Analysis Toolkit: a MapReduce framework for analyzing next-generation DNA sequencing data. Genome research 2010, 20(9):1297-1303.

84. Dobin A, Davis CA, Schlesinger F, Drenkow J, Zaleski C, Jha S, Batut P, Chaisson M, Gingeras TR: STAR: ultrafast universal RNA-seq aligner. Bioinformatics 2013, 29(1):15-21.

85. Liao Y, Smyth GK, Shi W: The Subread aligner: fast, accurate and scalable read mapping by seed-and-vote. Nucleic acids research 2013, 41(10):e108.

86. Ashburner M, Ball CA, Blake JA, Botstein D, Butler H, Cherry JM, Davis AP, Dolinski K, Dwight SS, Eppig JT et al: Gene ontology: tool for the unification of biology. The Gene Ontology Consortium. Nat Genet 2000, 25(1):25-29.

87. Database Resources of the National Genomics Data Center in 2020. Nucleic acids research 2020, 48(D1):D24-d33.

88. Emms DM, Kelly S: OrthoFinder: solving fundamental biases in whole genome comparisons dramatically improves orthogroup inference accuracy. Genome Biol 2015, 16(1):157-157.

89. Fischer L, Herkner C, Kitte R, Dohnke S, Riewaldt J, Kretschmer K, Garbe AI: Foxp3(+) Regulatory T Cells in Bone and Hematopoietic Homeostasis. Frontiers in endocrinology 2019, 10:578.

90. Thoenen E, Curl A, Iwakuma T: TP53 in bone and soft tissue sarcomas. Pharmacology & therapeutics 2019, 202:149-164.

91. Li C, Nguyen V, Clark KN, Zahed T, Sharkas S, Filipp FV, Boiko AD: Down-regulation of FZD3 receptor suppresses growth and metastasis of human melanoma independently of canonical WNT signaling. Proceedings of the National Academy of Sciences of the United States of America 2019, 116(10):4548-4557.

92. Cai T, Fjeldså J, Wu Y, Shao S, Chen Y, Quan Q, Li X, Song G, Qu Y, Qiao G et al: What makes the Sino- Himalayan mountains the major diversity hotspots for pheasants? Journal of Biogeography 2017.

93. Liu H, Luo Q, Zhang J, Mo C, Wang Y, Li J: Endothelins (EDN1, EDN2, EDN3) and their receptors (EDNRA, EDNRB, EDNRB2) in chickens: Functional analysis and tissue distribution. General and comparative endocrinology 2019, 283:113231.

94. Stuart CA, Stone WL, Howell ME, Brannon MF, Hall HK, Gibson AL, Stone MH: Myosin content of individual human muscle fibers isolated by laser capture microdissection. American journal of physiology Cell physiology 2016, 310(5):C381-389.

95. Toepfer CN, Garfinkel AC, Venturini G, Wakimoto H, Repetti G, Alamo L, Sharma A, Agarwal R, Ewoldt JF, Cloonan P et al: Myosin Sequestration Regulates Sarcomere

Function, Cardiomyocyte Energetics, and Metabolism, Informing the Pathogenesis of Hypertrophic Cardiomyopathy. Circulation 2020, 141(10):828-842.

96. Steiger SS, Kuryshev VY, Stensmyr MC, Kempenaers B, Mueller JC: A comparison of reptilian and avian olfactory receptor gene repertoires: species-specific expansion of group gamma genes in birds. BMC genomics 2009, 10:446.

97. Dong C, Yang XZ, Zhang CY, Liu YY, Zhou RB, Cheng QD, Yan EK, Yin DC: Myocyte enhancer factor 2C and its directly-interacting proteins: A review. Progress in biophysics and molecular biology 2017, 126:22-30.

98. Mackie EJ, Ahmed YA, Tatarczuch L, Chen KS, Mirams M: Endochondral ossification: how cartilage is converted into bone in the developing skeleton. The international journal of biochemistry & cell biology 2008, 40(1):46-62.

99. Potthoff MJ, Arnold MA, McAnally J, Richardson JA, Bassel-Duby R, Olson EN: Regulation of skeletal muscle sarcomere integrity and postnatal muscle function by Mef2c. Molecular and cellular biology 2007, 27(23):8143-8151.

100. Arnold MA, Kim Y, Czubryt MP, Phan D, McAnally J, Qi X, Shelton JM, Richardson JA, Bassel-Duby R, Olson EN: MEF2C transcription factor controls chondrocyte hypertrophy and bone development. Developmental cell 2007, 12(3):377-389.

101. Kofler R, Pandey RV, Schlötterer C: PoPoolation2: identifying differentiation between populations using sequencing of pooled DNA samples (Pool-Seq). Bioinformatics 2011, 27(24):3435-3436.

102. Cingolani P, Platts A, Wang le L, Coon M, Nguyen T, Wang L, Land SJ, Lu X, Ruden DM: A program for annotating and predicting the effects of single nucleotide polymorphisms, SnpEff: SNPs in the genome of Drosophila melanogaster strain w1118; iso-2; iso-3. Fly 2012, 6(2):80-92.

103. Naseer J, Anjum KM, Khan WA, Imran M, Ishaque M, Hafeez S, Munir MA, Nazir MAJIJoAR: Phylogenetic analysis based studies on genetic variation of Cytochrome b gene of Indian peafowl (Pavo cristatus) in Pakistan. 2017, 52(OF):343-346.

104. Armstrong MH, Braun EL, Kimball RT: Phylogenetic Utility of Avian Ovomucoid Intron G: A Comparison of Nuclear and Mitochondrial Phylogenies in Galliformes. 2001, 118 %J The Auk(3):799-804, 796.

105. Meng Y, Dai B, Ran J, Li J, Yue B: Phylogenetic position of the genus Tetraophasis (Aves, Galliformes, Phasianidae) as inferred from mitochondrial and nuclear sequences. Biochemical Systematics and Ecology 2008, 36(8):626-637.

106. Jaiswal SK, Gupta A, Saxena R, Prasoodanan VPK, Sharma AK, Mittal P, Roy A, Shafer ABA, Vijay N, Sharma VK: Genome Sequence of Peacock Reveals the Peculiar Case of a Glittering Bird. Front Genet 2018, 9:392.

107. Shibusawa M, Nishibori M, Nishida-Umehara C, Tsudzuki M, Masabanda J, Griffin DK, Matsuda Y: Karyotypic evolution in the Galliformes: an examination of the process of karyotypic evolution by comparison of the molecular cytogenetic findings with the molecular phylogeny. Cytogenetic and genome research 2004, 106(1):111-119.

108. Kaiser VB, van Tuinen M, Ellegren H: Insertion events of CR1 retrotransposable elements elucidate the phylogenetic branching order in galliform birds. Molecular biology and evolution 2007, 24(1):338-347.

109. Harrington WF, Rodgers ME: Myosin. Annual review of biochemistry 1984, 53:35-73.

110. Lu Q, Wang K, Lei F, Yu D, Zhao H: Penguins reduced olfactory receptor genes common to other waterbirds. Scientific reports 2016, 6:31671.

111. Khan I, Yang Z, Maldonado E, Li C, Zhang G, Gilbert MT, Jarvis ED, O'Brien SJ, Johnson WE, Antunes A: Olfactory Receptor Subgenomes Linked with Broad Ecological Adaptations in Sauropsida. Molecular biology and evolution 2015, 32(11):2832-2843.

112. Krause ET, Krüger O, Kohlmeier P, Caspers BA: Olfactory kin recognition in a songbird. Biology letters 2012, 8(3):327-329.

113. Holland RA, Thorup K, Gagliardo A, Bisson IA, Knecht E, Mizrahi D, Wikelski M: Testing the role of sensory systems in the migratory heading of a songbird. The Journal of experimental biology 2009, 212(Pt 24):4065-4071.

114. Blackburn T, Gaston K: The Distribution of Body Sizes of the World's Bird Species. Oikos 1994, 70:127-130.

115. Jones RG, Pearce EJ: MenTORing Immunity: mTOR Signaling in the Development and Function of Tissue-Resident Immune Cells. Immunity 2017, 46(5):730-742.

116. Xu J, Ji J, Yan XH: Cross-talk between AMPK and mTOR in regulating energy balance. Critical reviews in food science and nutrition 2012, 52(5):373-381.

117. Buijs JT, Henriquez NV, van Overveld PG, van der Horst G, ten Dijke P, van der Pluijm G: TGF-beta and BMP7 interactions in tumour progression and bone metastasis. Clinical & experimental metastasis 2007, 24(8):609-617.

118. Huang X, Zhong L, Post JN, Karperien M: Co-treatment of TGF-β3 and BMP7 is superior in stimulating chondrocyte redifferentiation in both hypoxia and normoxia compared to single treatments. Scientific reports 2018, 8(1):10251.

119. Fang G, Jia X, Li H, Tan S, Nie Q, Yu H, Yang Y: Characterization of microRNA and mRNA expression profiles in skin tissue between early-feathering and late-feathering chickens. BMC genomics 2018, 19(1):399.

120. Trajanoska K, Rivadeneira F, Kiel DP, Karasik D: Genetics of Bone and Muscle Interactions in Humans. Current osteoporosis reports 2019, 17(2):86-95.

121. Liu CF, Samsa WE, Zhou G, Lefebvre V: Transcriptional control of chondrocyte specification and differentiation. Seminars in cell & developmental biology 2017, 62:34-49.

122. Tang R, Xu X, Yang W, Yu W, Hou S, Xuan Y, Tang Z, Zhao S, Chen Y, Xiao X et al: MED27 promotes melanoma growth by targeting AKT/MAPK and NF-κB/iNOS signaling pathways. Cancer letters 2016, 373(1):77-87.

123. Robic A, Morisson M, Leroux S, Gourichon D, Vignal A, Thebault N, Fillon V, Minvielle F, Bed'Hom B, Zerjal T et al: Two new structural mutations in the 5' region of the ASIP gene cause diluted feather color phenotypes in Japanese quail. Genetics, selection, evolution : GSE 2019, 51(1):12.

124. Domyan ET, Guernsey MW, Kronenberg Z, Krishnan S, Boissy RE, Vickrey AI, Rodgers C, Cassidy P, Leachman SA, Fondon JW, 3rd et al: Epistatic and combinatorial effects of pigmentary gene mutations in the domestic pigeon. Current biology : CB 2014, 24(4):459-464.

125. Watt B, van Niel G, Raposo G, Marks MS: PMEL: a pigment cell-specific model for functional amyloid formation. Pigment cell & melanoma research 2013, 26(3):300-315.

126. Ishishita S, Takahashi M, Yamaguchi K, Kinoshita K, Nakano M, Nunome M, Kitahara S, Tatsumoto S, Go Y, Shigenobu S et al: Nonsense mutation in PMEL is associated with yellowish plumage colour phenotype in Japanese quail. Scientific reports 2018, 8(1):16732.

127. Kerje S, Sharma P, Gunnarsson U, Kim H, Bagchi S, Fredriksson R, Schütz K, Jensen P, von Heijne G, Okimoto R et al: The Dominant white, Dun and Smoky color variants in chicken are associated with insertion/deletion polymorphisms in the PMEL17 gene. Genetics 2004, 168(3):1507-1518.

128. Andersson LS, Wilbe M, Viluma A, Cothran G, Ekesten B, Ewart S, Lindgren G: Equine multiple congenital ocular anomalies and silver coat colour result from the pleiotropic effects of mutant PMEL. PloS one 2013, 8(9):e75639.

# Figures legend

**Fig. 1. Photographs of the Indian blue peafowl and white peafowl**. The Indian blue peafowl and white peafowl showed in **a** and **b**, respectively.

**Fig. 2. The global maps of de novo genome assembly of Indian peafowl**. **a**, 55 scaffolds with the length greater than 5 Mb (scaffolds N70) of the assembled Indian blue peafowl. The perimeter of ring represented the length of scaffolds, and the light orange links in the middle circle indicated the synteny in the peafowl genome. The GC density, gene density and tandem repeat sequence density of peafowl genome were displayed in **b**, **c** and **d**, respectively. And the red and green bars in the gene density diagram represented the positive strand (+) and negative strand (-) in peafowl genome.

**Fig. 3. Gene family and genome evolution among the peafowl and other 14 species**. **a**, Statistics of orthologs among 15 species. “1:1:1” indicated the single-copy orthologs were shared by 15 species with one copy. “N:N:N” represented any other orthologous group (missing in one species). Specices-specific showed the specific orthologs in each species. Other orthologs were unclustered into gene families. **b**, Venn diagram of the shared orthologous gene families among the Phasianidae species (peafowl, Japanese quail, chicken and turkey). The numbers represented the unique or common gene family among the species. **c,** The phylogenetic relationship tree among 15 species was constructed by maximum likelihood with JTT model based on the single-copy orthologous sequences, human and mouse as outgroups. The divergence time of species was estimated by five calibration time from TimeTree database, including human-mouse (85~97Mya), human-zebra finch (294~323Mya), zebra finch-medium ground

finch (30.4~46.8Mya), common mallard-zebra finch (93.2~104.6Mya) and saker falcon-peregrine falcon (1.66~3.68Mya). Of them, the divergence time of human and mouse was used as a timeline at the bottom of the figure which was divided into four different periods such as tertiary, cretaceous, jurassic and triassic, and showed by different colours. In addition, the expansion and contraction of gene family in 15 species were showed at the right of species name. The red (+) and blue (-) numbers represented the expanded and contracted genes, respectively.

**Fig. 4. Genome synteny and collinearity among the Indian peafowl, chicken, and turkey. a**, A syntenic map of the peafowl and turkey genomes. The perimeter of ring represented the length of chromosomes labeled by different colours or scaffolds. It displayed the scaffolds eith the length greater than 5 Mb (scaffolds N70) of the assembled Indian blue peafowl, of which, the first 32 scaffolds was showed in red and other scaffolds were marked in gray. **b**, A syntenic map of the peafowl and chicken genomes. The first 35 scaffolds were showed in red, and other scaffolds were marked in gray. **c**, The distribution of Ka/Ks ratio in the genomes of peafowl, turkey, and chicken.

**Fig. 5. Causal genes for white feather in blue and white peafowl**. **a**, Allele frequency differences between blue and white peafowl. Scaffolds were distinguished by different colours. The candidate SNPs along with causal genes were marked by arrows, including *EDNRB* and *PMEL*. **b**, Differentially expressed genes (DEGs) related to the plumage pigmentation. The red and blue dots were used to mark the up- and down- regulated genes in blue and white peafowl, respectively. A total of 69 down-regulated genes and 52 up-regulated genes were identified, of which ten up-regulated genes were associated with the melanin deposition, and so marked out. **c**, KEGG and GO enrichment of DEGs

related to the plumage pigmentation in blue and white peafowl. The darker the colour was, the more significant the difference. The top significant pathway was enriched in the process of the melanin synthesis based on the criterion of $P < 0.05$ as significant. **d**, Allele frequencies of differentially expressed genes in blue and white peafowl. B: blue peafowl, W: white peafowl. An observation showed that the top two differential sites were located in *PMEL* and *EDNRB*. **e**, *PMEL* transcripts in the feather pulp of blue and white peafowl. B: blue peafowl, W: white peafowl. The RNA sequencing reads of *PMEL* were aligned to the assembly peafowl genome in the feather tissue of blue and white peafowl. The red arc represented the mRNA expression level of *PMEL*. Apparently, *PMEL* was normally expressed in blue peafowl but almost not expressed in white peafowl. **f**, RT- qPCR of *PMEL* transcripts in the feather pulp of blue and white peafowl. The result indicated that the relative expression of *PMEL* was significantly decreased in white peafowl compared to the mRNA expression of *PMEL* in blue peafowl ($P = 0.013$).

# Tables

**Table 1. Quality metrics for the peafowl genome assembly generated in the current work and for other peafowl genome assemblies published in previous studies.**

| Items | This study | Shubham et al. (2018) | Ruby et al. (2019) |
|---|---|---|---|
| Sequencing technology | Illumina NovaSeq 6000, PacBio RS-II, 10X Genomics, Chicoga | Illumina NextSeq 500 | Illumina HiSeq, ONT |
| Total sequencing depth | 362× | 136× | 236× |
| Total scaffolds | 726 | 98,687 | 179,332 |
| Scaffolds N50 (bp) | 11,421,185 | 25,613 | 190,304 |
| Contigs N50 (bp) | 6,188,159 | 19,387 | 103,131 |
| Longest scaffold length (bp) | 38,857,732 | 286,113 | 2,488,982 |
| Total sequence length (bp) | 1,046,718,946 | 1,137,150,029 | 1,027,510,962 |
| Total number of predicted protein-coding genes | 19,465 | 15,970 | 23,153 |

## Supplementary materials

**Supplementary Figure S1**. Pipeline of the draft genome assembly of Indian blue peafowl

**Supplementary Figure S2**. Workflow of the genome annotation of Indian blue peafowl

**Supplementary Figure S3**. 17-kmer frequency distribution of peafowl genome

**Supplementary Figure S4**. Divergence distribution of transposable element of peafowl genome by using RepeatMasker software

**Supplementary Figure S5**. Phylogenetic tree of 15 species constructed with IQ-tree

**Supplementary Figure S6**. Phylogenetic tree of 15 species constructed with RAxML

**Supplementary Figure S7**. *EDNRB* transcripts in the feather tissue of peafowl by IGV visualization

**Supplementary Table S1**. Statistics of genome assembly data of peafowl

**Supplementary Table S2**. Summary of de novo genome assembly of peafowl

**Supplementary Table S3**. Percentage of the base contents of peafowl genome

**Supplementary Table S4**. Statistics of paired-end reads mapping in peafowl genome

**Supplementary Table S5**. Number of SNPs of peafowl genome

**Supplementary Table S6**. Assembly assessment of completeness by using BUSCOs

**Supplementary Table S7**. Whole genome repetitive sequences of Indian peafowl genome predicted by homologous alignment and de novo search

**Supplementary Table S8**. Composition of repetitive sequences in peafowl genome

**Supplementary Table S9**. Prediction of protein-coding genes for peafowl genome

**Supplementary Table S10**. Statistics of functional annotation of protein-coding genes in the peafowl genome assembly

**Supplementary Table S11**. Statistics of non-coding RNAs in the assembly of peafowl

**Supplementary Table S12**. Functional enrichment of species-specific genes in peafowl compared with the Phasianidaes (chicken, turkey, and Japanese quail)

**Supplementary Table S13**. Functional categories of positively selected genes (dN/dS>1) between peafowl and chicken

**Supplementary Table S14**. Functional categories of positively selected genes (dN/dS>1) between peafowl and turkey

**Supplementary Table S15**. Functional enrichment of significantly expansive genes in peafowl

**Supplementary Table S16**. Functional enrichment of significantly contractive genes in peafowl

**Supplementary Table S17**. GO terms enrichment of positively selected genes in peafowl under branch-site model

**Supplementary Table S18**. KEGG pathways of positively selected genes in peafowl under branch-site model

**Supplementary Table S19**. Functional categories of positively selected genes in peafowl under branch model

**Supplementary Table S20**. Primer sequences of *PMEL* for RT-qPCR

Figure 1

Click here to access/download;Figure;Figure 1.tif

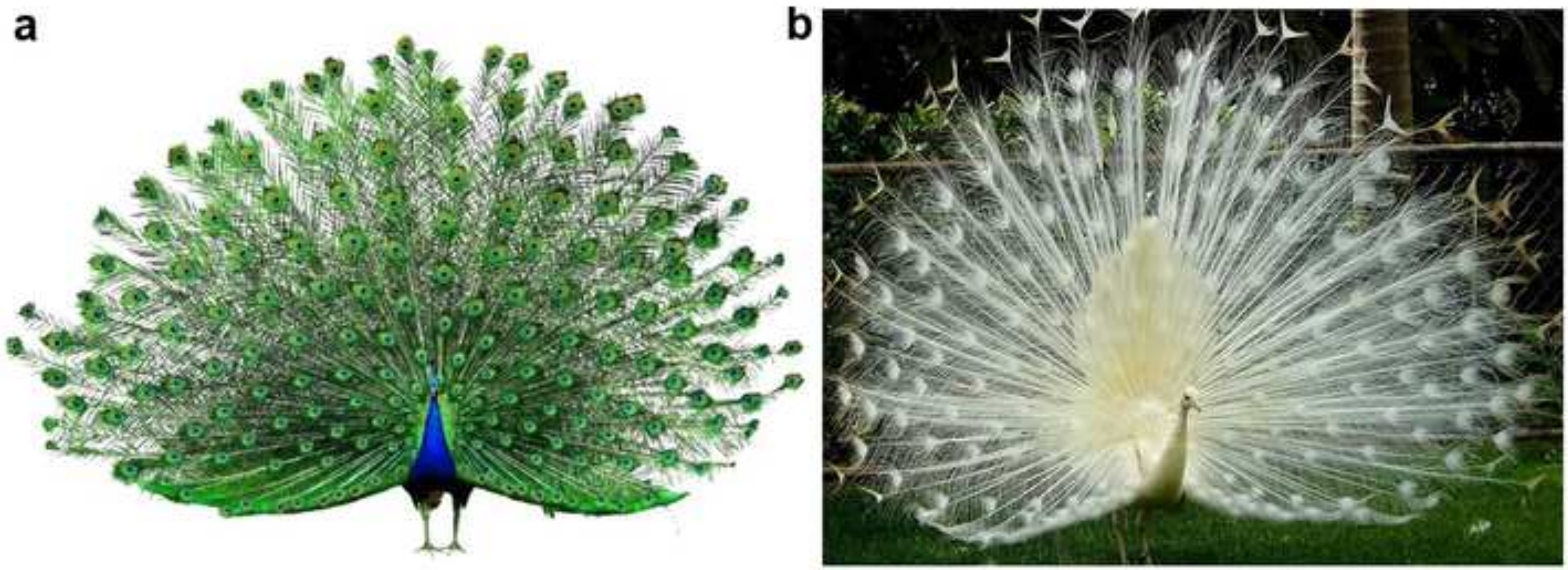

Figure 2

Click here to access/download;Figure;Figure 2.tif

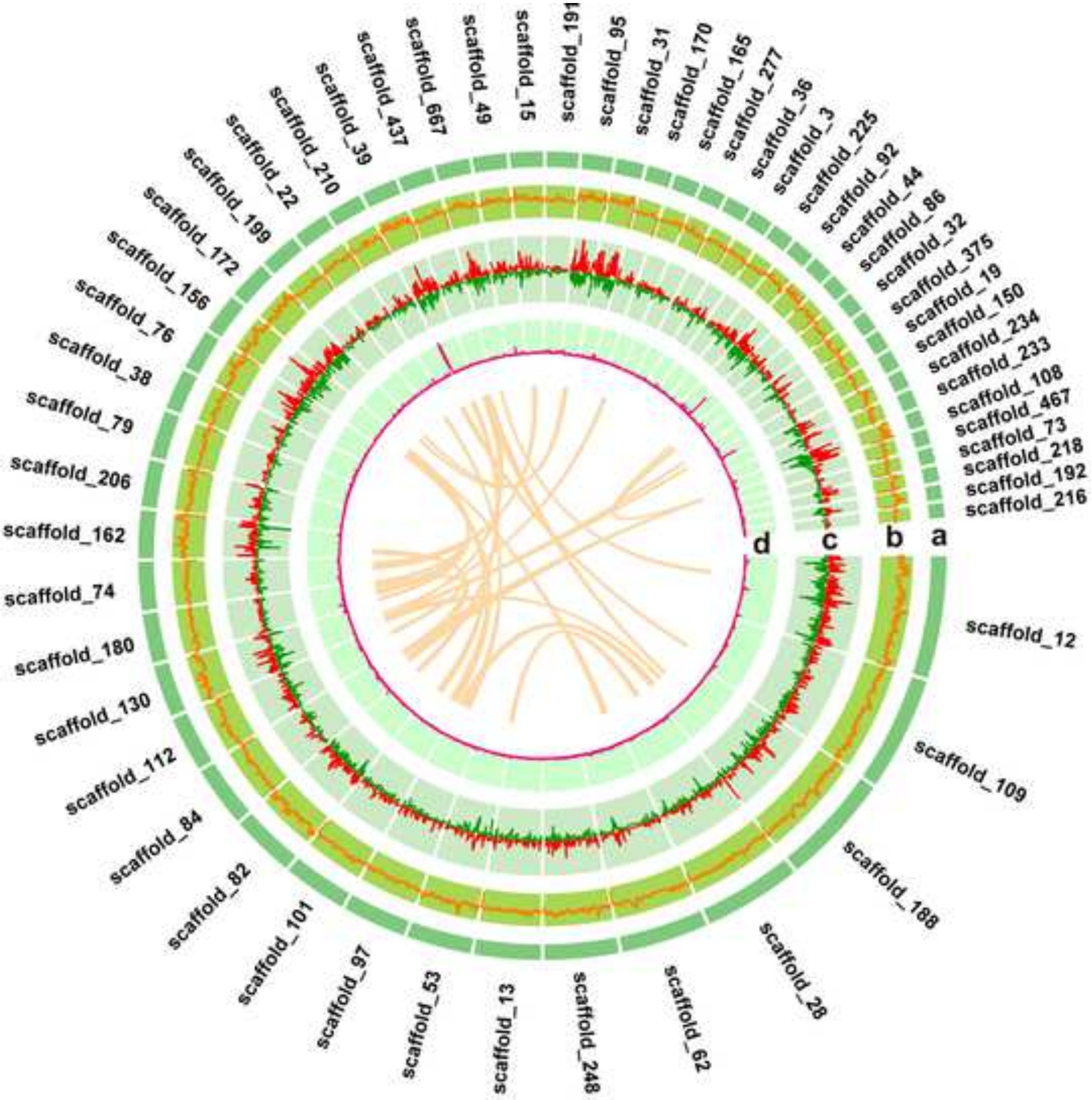

Figure 3

Click here to access/download;Figure;Figure 3.tif

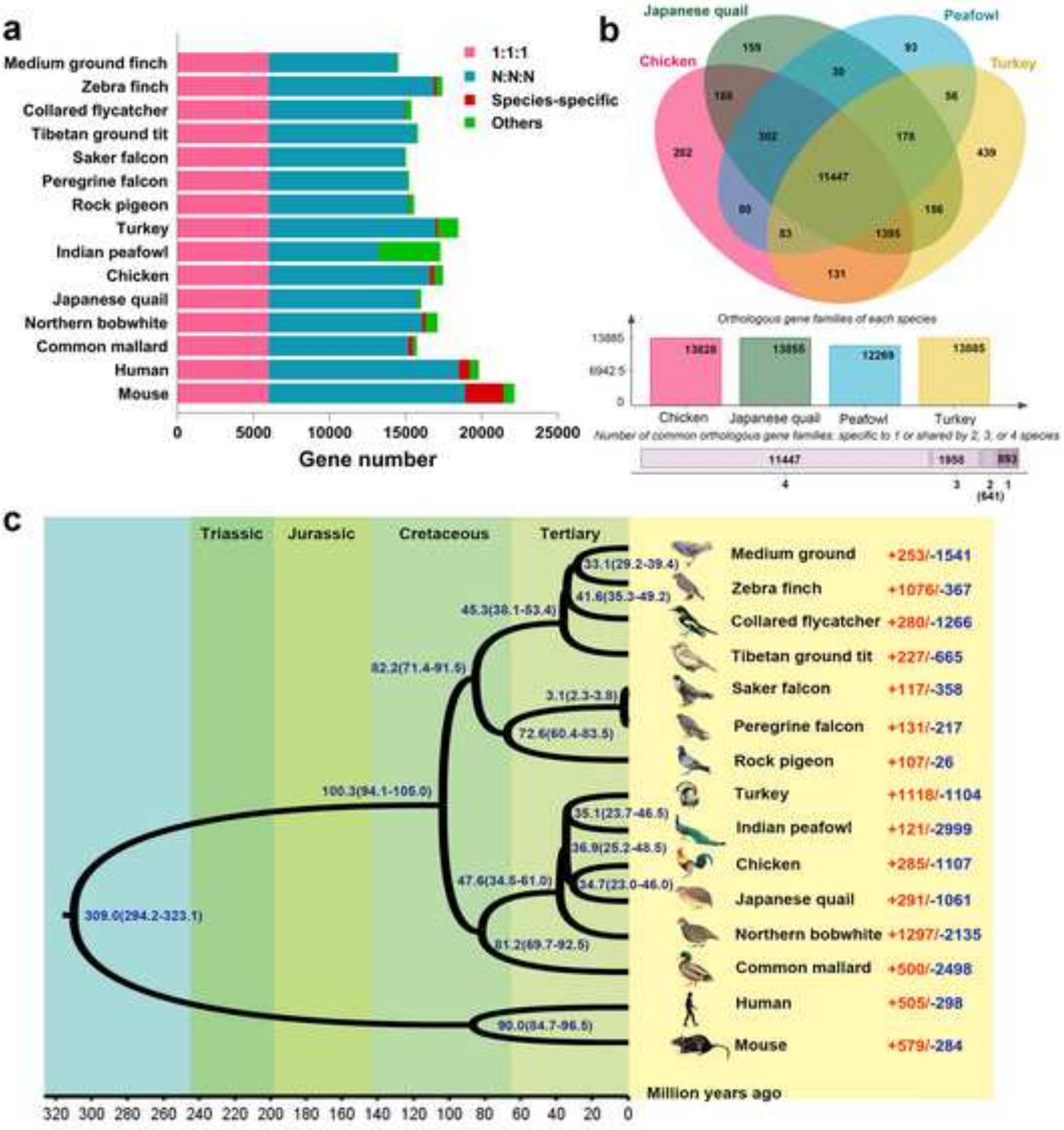

Figure 4

Click here to access/download;Figure;Figure 4.tif

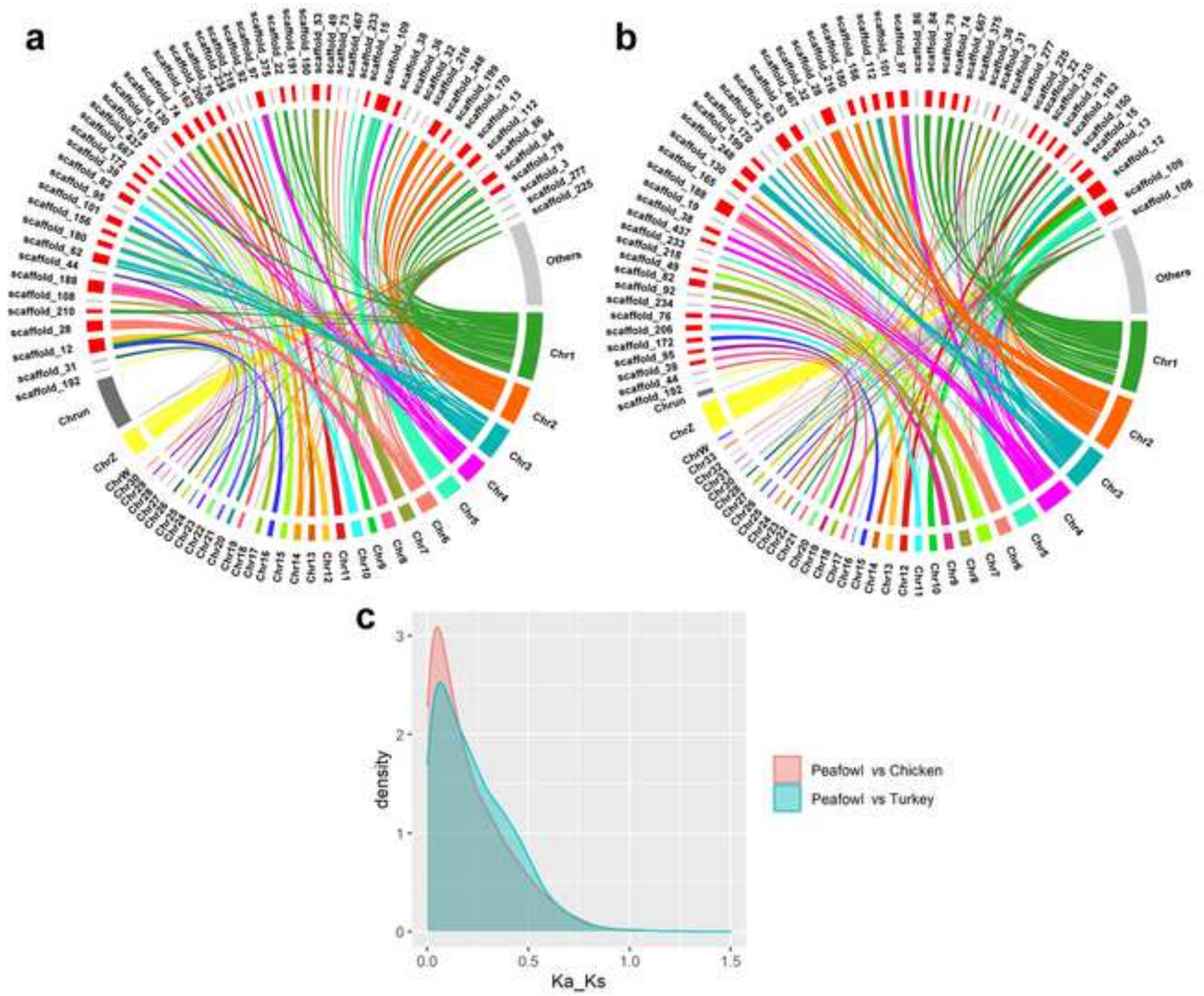

Figure 5

Click here to access/download;Figure;Figure 5.tif

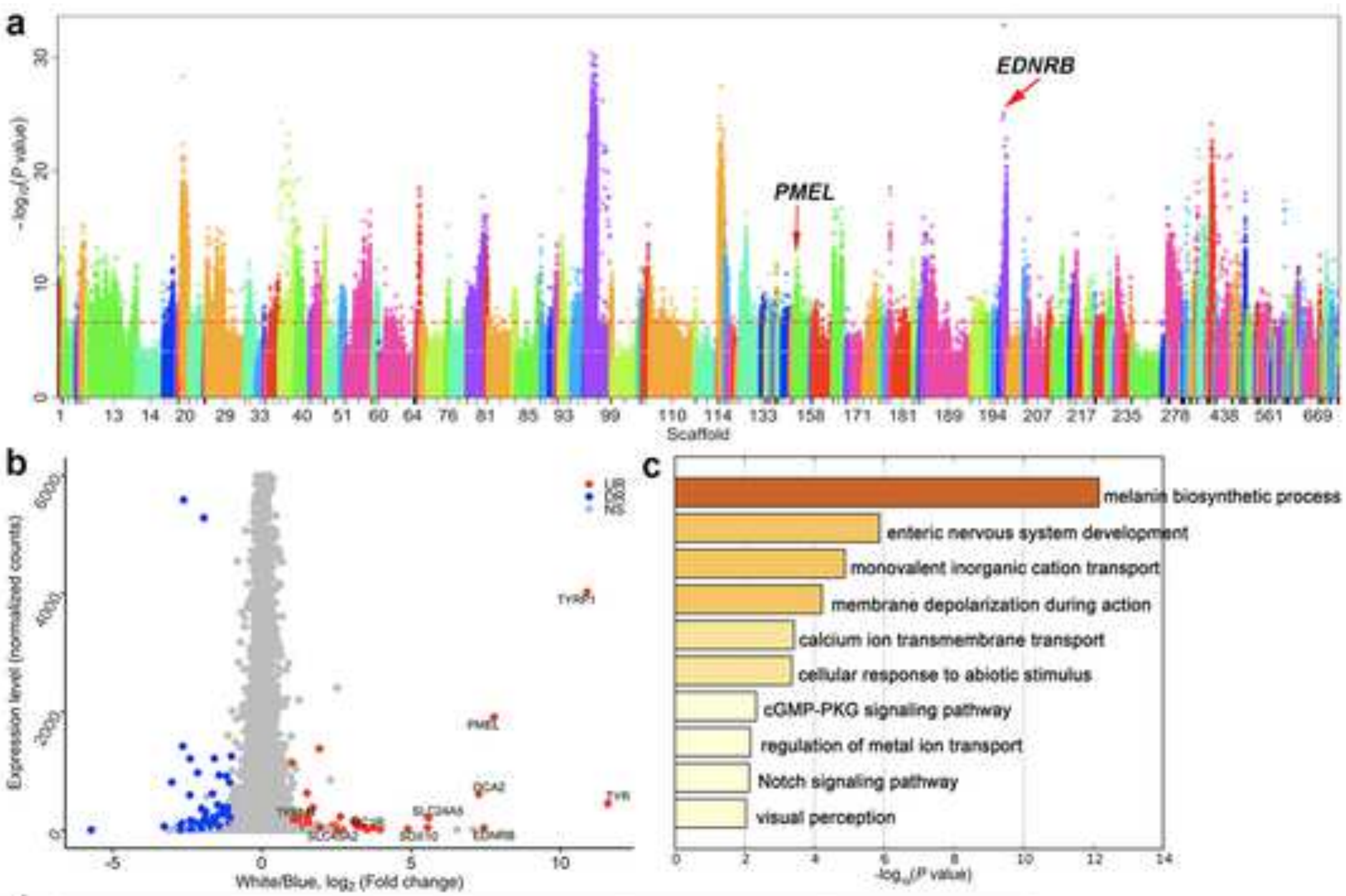


d

| Scaffold | Position | Gene | Ref | Alt | Variant type | Ref | | Alt | |
|---|---|---|---|---|---|---|---|---|---|
| | | | | | | B_freq | W_freq | B_freq | W_freq |
| 144 | 428756 | PMEL | C | T | Synonymous | 0.573 | 0.063 | 0.427 | 0.937 |
| 144 | 428768 | PMEL | C | T | Synonymous | 0.568 | 0.065 | 0.432 | 0.935 |
| 196 | 718028 | EDNRB | G | A | Synonymous | 0.983 | 0 | 0.017 | 1 |

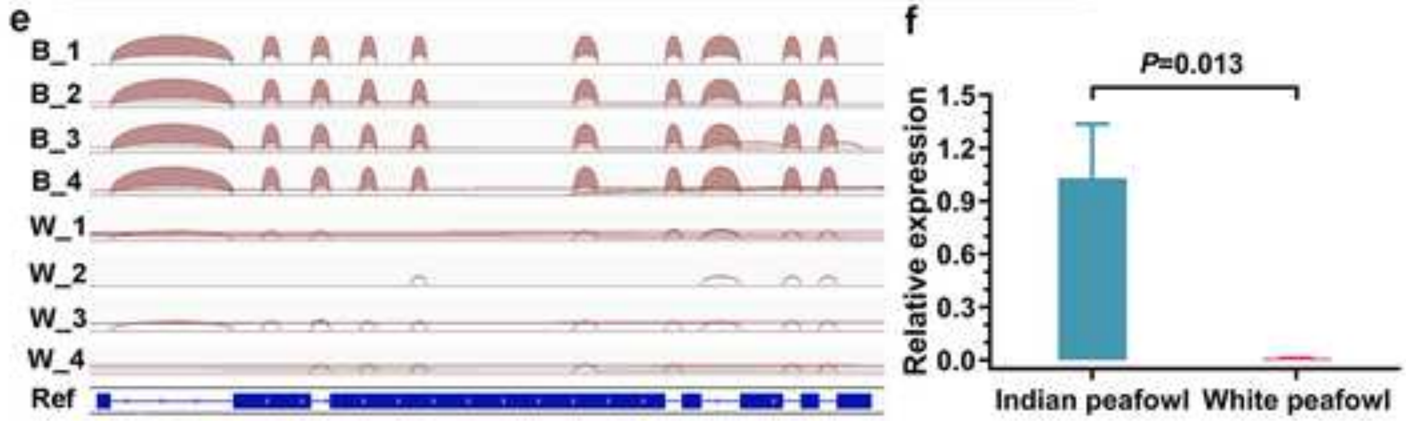

Click here to access/download
**Supplementary Material**
Supplementary materials.docx

Supplementary Table S12-Table S19

Click here to access/download
**Supplementary Material**
Supplementary Table S12-Table S19.xlsx
