# Supplementary material for: A high-quality assembly reveals genomic characteristics, phylogenetic status, and causal genes for leucism plumage of Indian peafowl
Source: Gigascience. 2022 Apr 6;11:giac018. doi: 10.1093/gigascience/giac018 (PMC8985102; doi:10.1093/gigascience/giac018)
Supplement: giac018_GIGA-D-21-00190_Revision_1 [file giac018_giga-d-21-00190_revision_1.pdf]

GigaScience

A high-quality assembly reveals genomic characteristics, phylogenetic status and causal genes for white feather of Indian peafowl

--Manuscript Draft--

**Manuscript Number:**	GIGA-D-21-00190R1
**Full Title:**	A high-quality assembly reveals genomic characteristics, phylogenetic status and causal genes for white feather of Indian peafowl
**Article Type:**	Research
**Funding Information:**	educational commission of jiangxi province of china — Dr. huirong Mao<br>key research and development program of jiangxi province — Dr. huirong Mao
**Abstract:**	Background<br><br>The dazzling phenotypic characteristics of male Indian peafowl (Pavo cristatus) are attractive to both the female of the species and to humans. However, little is known about the evolution of the phenotypic and phylogeny of these birds at the whole-genome level. So far, there are no reports regarding the genetic mechanism of the formation of white feathers in this variant of Indian peafowl.<br><br>Results<br><br>A draft genome of Indian peafowl was assembled, with a genome size of 1.05 Gb (the sequencing depth is 362×), and contig and scaffold N50 were up to 6.2 Mb and 11.4 Mb, respectively. Compared with other birds, Indian peafowl showed changes in terms of metabolism, immunity, skeletal development and feather development, which provided a novel insight into the phenotypic evolution of peafowl, such as the large body size and feather morphologies. Moreover, we determined that the phylogeny of Indian peafowl was more closely linked to turkey than chicken. Specifically, we first identified that PMEL was a potential causal gene leading to the formation of the white feather variant in Indian peafowl.<br><br>Conclusions<br><br>This study provides an Indian peafowl genome of high-quality as well as a novel understanding of phenotypic evolution and phylogeny of Indian peafowl. These results provide a valuable reference for the study of avian genome evolution. Furthermore, the discovery of the genetic mechanism for the development of white feathers is both a breakthrough in the exploration of peafowl plumage, and also offers clues and directions for further investigations of the avian plumage coloration and artificial breeding in peafowl.
**Corresponding Author:**	huirong Mao<br>Jiangxi Agricultural University<br>Nanchang, Jiangxi CHINA
**Corresponding Author Secondary Information:**	
**Corresponding Author's Institution:**	Jiangxi Agricultural University
**Corresponding Author's Secondary Institution:**	
**First Author:**	Shaojuan Liu
**First Author Secondary Information:**	
**Order of Authors:**	Shaojuan Liu<br>Hao Chen

Powered by Editorial Manager® and ProduXion Manager® from Aries Systems Corporation

	Jing Ouyang
	Min Huang
	Hui Zhang
	Sumei Zheng
	Suwang Xi
	Hongbo Tang
	Yuren Gao
	Yanpeng Xiong
	Di Cheng
	Kaifeng Chen
	Bingbing Liu
	Wanbo Li
	Xueming Yan
	huirong Mao
	Jun Ren
**Order of Authors Secondary Information:**	
**Response to Reviewers:**	Hongfang Zhang<br>Editor, GigaScience<br>Nov 15, 2021<br><br>Dear Dr. Hongfang Zhang,<br><br>Thank you for your letter and for the reviewers’ comments concerning our manuscript entitled "A high-quality assembly reveals genomic characteristics, phylogenetic status and causal genes for white feather of Indian peafowl" (GIGA-D-21-00190). We greatly appreciate the opportunity to further revise this manuscript.<br><br>We sincerely appreciate the very thoughtful and constructive comments from the editor(s) and reviewers that help us to improve this manuscript. We hope our revised draft can meet your requirements and be published. Our article was polished by a professional editing company and we believe that the readability of polished article increased greatly. In addition, we carefully checked the style of revised manuscript follows the “Instruction for Authors”. On the basis the reviewers' comments, we have revised the description of instruction and phylogenetic analysis. We hope that the revised manuscript now meets the standards required for publication in GigaScience.<br><br>Revised portion are marked in red in the paper. We sincerely hope that these revisions would satisfy you. The point-to-point responses to the comments are shown as follows. Please do not hesitate to contact me if you have any other questions or comments.<br><br>With best regards,<br>Huirong Mao<br>Email: maohuirong82@hotmail.com

Powered by Editorial Manager® and ProduXion Manager® from Aries Systems Corporation

Response to the reviewers' comments:
Reviewer # 1
This study generates a new genome assembly of the Indian peafowl and uses it to explore a variety of questions. Overall, I found the study to be well done and thorough. The authors generate a well-resolved genome using three sequencing approaches (Illumina, PacBio long-read, and 10×) and then perform a number of phylogenetic and functional/molecular evolution analyses to explore a series of questions. As someone who does not build genomes, my comments are more about the structure of the paper and the phylogenetic analysis. I have a few comments and concerns below:

1) I found this paper very hard to read in places. There are numerous grammatical errors throughout, and in places it is sometime hard to decipher the meaning of what has been written. A revision will need substantial help in the writing.
Response: We are grateful to your positive feedback on our study and greatly appreciate these valuable comments, which help us build our confidence in revising our paper comprehensively. We are deeply sorry about the writing, the revised article was polished by a professional editing company.

2) The introduction went off in a number of different tangents, making it hard to follow what the authors were actually doing. It would help to more clearly lay out the study goals (e.g. the phylogenetic component, functional component, etc.) and keep the introduction more focused. The final paragraph does this to some degree, but laying out the goals earlier would help in understanding the introduction.
Response: We are grateful to this constructive comment. We are very sorry for vaguely expressing the meaning. Here, we have reorganized the introduction and made it clear.

3) It is unclear how the other bird species were chosen for the phylogenetic analyses. There are now many more bird genomes available than used here. Rather than arbitrarily choosing a dozen, it would be better to try and fit the peafowl into the framework of previously analyses (see papers by Erich Jarvis's group). I am a bit skeptical about the relationships between the peafowl and the turkey and chicken with so few species used in the analysis. It would strengthen this section to include more species and generate a more robust phylogeny.
Response: Thanks for your advice. The other bird species we chosen were referenced from previous bird references about comparative genomic analysis (doi: 10.1093/gigascience/giy049; doi: 10.1093/gigascience/giy044; doi: 10.1093/gigascience/giy113; doi: 10.3389/fgene.2018.00392; et al.). In addition, the focus of our study is the adaptive and phenotypic evolution of peafowl by comparative genomic analysis. The fewer species we retained, the more single-copy homologous genes we obtained in the analysis, which is more accurate to estimate the evolutionary relationship of species. Moreover，the construction of phylogenetic relationship between peafowl and other birds is a critical background for subsequent comparative genomic analysis. Thus，the current results revealed that peafowl is mostly related to the turkey than chicken among the 15 selected species. We have rewritten many statements and improper expression about the phylogeny in the introduction, results and discussion referred to your suggestion.

4) Figure 5A. Why were only PMEL and ENDRB discussed in the text and highlighted here, when there are other genes of equal or higher significance showing up? I realize these two genes have been implicated in other feather color studies, but so have many of the other genes you list (e.g. TRYP1, TYR, PMEL, SLC24A5, MC1R, etc.). What do the RNAseq data show about these genes? I think you are overemphasizing two genes and leaving out some critical information about a host of other important genes. I would like to see a more balanced discussion in this section.
Response: Thanks. Plumage colours are often determined by causal genes that may have a difference in allele frequency between different plumage colour populations. On the one hand, we used resequencing data to make selective signal analysis by detecting the allele frequency difference in blue and white peafowl results suggested that only PMEL and EDNRB were involved in pigmentation. On the other hand, we used RNA-seq data to determine the DEGs between blue and white peafowl, and results indicated that 10 significantly up-regulated genes were associated with melanin deposition. Subsequently, we used resequencing data to identify the allele imbalance difference sites of 10 up-regulated genes and found that only PMEL and EDNRB had differential sites. Although there were many significant sites in the allele frequency

difference, they did not cause differences in transcripts and resulted in the differential expression of related genes in the analysis of DEGs, with the exception of PMEL and EDNRB. By overlapping with the results based on resequencing and RNA-seq data, we determined that PMEL and EDNRB were candidate genes for the formation of white plumage in peafowl. Furthermore, we observed the transcripts of PMEL and EDNRB based on RNA-seq data by IGV visualization and discovered that PMEL was hardly expressed in white peafowl compared to blue peafowl; moreover, EDNRB was normally expressed in both variants. Finally, we verified the low mRNA expression of PMEL in white peafowl by RNT-qPCR; this results, suggested that PMEL was a strong candidate causative gene for the formation of white plumage.

Reviewer # 2
The authors had finished very systematic and comprehensive research. They obtained a near-chromosomal reference genome by combined several sequencing technologies and completeness of the assembled genome had reached 97.4%. In addition, it is finding a causal gene of white plumage that identifies an important gap on the genetic mechanism of the white plumage in the peafowl. The results and resources obtained from this study are valuable further comparative genomic studies in birds. The analyses are also sound and comprehensive. However, the writing of the paper is not concise and focused. The first concern is that I don't think this study is able to resolve the phylogenetic position of Pavo cristatus if whole genome data of only five species of Galliforme species were involved. Rather the purpose of phylogeny was for comparative genomics. Therefore, they should down-tune their texts about phylogeny in the introduction and discussion. Second, the apparent sister species of Pavo cristatus is Pavo muticus. The de novo assembly of this species has been published (see Dong et al. 2021 Proc. R. Soc. B.288202100732021007[3http://doi.org/10.1098/rspb.2021.0073](http://doi.org/10.1098/rspb.2021.0073)). I wonder why author did not include the data of this species in order to increase power of their comparative genomic analysis.There are also other minor comments and suggestions for author I provide below：

Response: Thank you for your positive feedback and greatly appreciate your valuable suggestion about phylogenetic analysis in this article. (1) Firstly, concerning about the phylogenetic position of Pavo cristatus, the focus of our study is the adaptive and phenotypic evolution of peafowl by comparative genomic analysis. The construction of phylogenetic relationship between peafowl and other birds is a critical background for comparative genomic analysis. In the article, the current results only mean that peafowl is closer to turkey than chicken among the 15 selected species. Thus, we have decreased the description about phylogeny in the introduction and discussion according to your advice. Additionally, according to your advice, we supplemented and used all the available Phasianidae genome and only added two species (Bambusicola thoracica and Phasianus colchicus) to construct the phylogenetic tree. We found that the relationship of chicken and Bambusicola thoracica was closer, the genetic distance between turkey and Phasianus colchicus was closer and the phylogeny of peafowl was not changed. This new result is not much different from the current result in this study. The phylogenetic tree supplemented was shown below (supplementary figure 1 in response letter). (2) Second, about the de novo assembly of green peafowl, we also considered this species but it had not been published yet when we were working on this study. At present, although we attempt to include the data of green peafowl genome in comparative genomic analysis according to your advice, we are unable to download the annotation files of gene structure and function because the authors have not uploaded them in the NCBI. Additionally, even if we include the green peafowl in the comparative genomics, the result of phylogenetic tree should be show that the blue peafowl is close to the green peafowl. Because blue peafowl and green peafowl are different species of the same genus and have closer relationship than other Phasianidae birds. The focus of our study is not the phylogenetic position of peafowl. Thus, we think whether include the green peafowl genome data for comparative genomic analysis may be little effect on the results.

Powered by Editorial Manager® and ProduXion Manager® from Aries Systems Corporation

1. Line 47-57: This paragraph is less focused and authors should be more cautious about their language. Some sentences are not professional and science. For instance, "Pavo cristatus, xxxx is the king of birds".
Response: Thanks for your advice. We are very sorry for vaguely expressing the meaning. Here, we have revised this paragraph and made it clear.

2. Line 58: Author should cite some recent papers about avian genomes, for instance, Feng et al 2020 in Nature provide a dense sampling of avian genomes.
Response: Thanks a lot. We have revised it and cited some recent papers about avian genomes.

3. Line 70-82: I think this paragraph should be rephrased for two reasons: first authors did not cite some recent papers about the most updated phylogenies of Galliformes (e.g.Wang et al. PloS one, 8(5), e643120). So there arguments on the uncertainties of phylogenetic position of Pavo cristatus to some of its closely related species are not valid. Second, the focus of the present study is not the precise phylogenetic position of Pavo cristatus. Therefore, I suggest the authors to rephrase this paragraph.
Response: Thanks for your valuable suggestions. We agree that the focus of this study is not the precise phylogenetic position of Pavo cristatus but the comparative genomics. Here, we have rephrased this paragraph.

4. Line 222: How did you determine convergence of your results?
Response: We use the Tracer software to analyze the output file “mcmc.txt” and detect its efficient sampling size (ESS) value. When all of ESS value is greater than 200, we consider that the iterative operation reaches convergence. Otherwise, if it does not converge, we need to adjust the number of burnin, samplefreq or nsample and rerun the MCMCtree program until convergence.

5. Line 310-367: This part is very descriptive and needs to be condensed.
Response: Thanks for your advance. We have condensed this part.

6. Line 369: If the authors really want to reconstruct the precise phylogenetic position of Pavo cristatus, they should involve all published de novo assemblies of birds from the family Galliformes. The current results only means that among the 15 selected species, Pavo cristatus is mostly related to the turkey.
Response: Thanks for this valuable suggestion. The focus of our study is the adaptive and phenotypic evolution of peafowl by comparative genomic analysis. The construction of phylogenetic relationship between peafowl and other birds is a critical background for comparative genomic analysis. In fact, the current results only indicate that peafowl is closer to the turkey than chicken among the 15 selected species. Thus, we have revised statements about the phylogeny of peafowl in the paper.

7. Line 377-392: This part reads like the part that should appear in Discussion.
Response: Thanks. We have revised and moved this part in Discussion.

8. Line 582-611: The structure here is not well organize and statements are not tightly linked to results.
Response: In combination with your previous suggestions, we have reorganized this part of the results and discussion and reduced the statements of the phylogeny part in discussion.

9. Line 623-627: I don't agree with these statement. So authors expected wild populations would show different patterns?
Response: Thanks for your question. We just make a hypothesis that the function of olfaction and flying of peafowl may be weakened under the condition of artificial long-term domestication. However, whether there are different patterns to wild peafowl needs further investigation.

10. Lines 679-681：the results of detecting the SNP and Indel variation did not appear in the "Results" section.
Response: Thanks. In order to save computing resources and improve operating speed, we only detected the SNP and Indel of the scaffold 144 that PMEL was located in and did not detect the all SNP and Indel of sequencing data. Thus, we did not put

Powered by Editorial Manager® and ProduXion Manager® from Aries Systems Corporation

	the results of SNP and Indel variation in the “Results” section.<br><br>11. The language of the paper is acceptable but would definitely benefit from editing by a native speaker.<br>Response: Thanks for your advice. The revised article was polished by a professional editing company.
Additional Information:	
Question	Response
Are you submitting this manuscript to a special series or article collection?	No
**Experimental design and statistics**<br><br>Full details of the experimental design and statistical methods used should be given in the Methods section, as detailed in our Minimum Standards Reporting Checklist. Information essential to interpreting the data presented should be made available in the figure legends.<br><br>Have you included all the information requested in your manuscript?	Yes
**Resources**<br><br>A description of all resources used, including antibodies, cell lines, animals and software tools, with enough information to allow them to be uniquely identified, should be included in the Methods section. Authors are strongly encouraged to cite Research Resource Identifiers (RRIDs) for antibodies, model organisms and tools, where possible.<br><br>Have you included the information requested as detailed in our Minimum Standards Reporting Checklist?	Yes
**Availability of data and materials**<br><br>All datasets and code on which the conclusions of the paper rely must be either included in your submission or deposited in publicly available repositories (where available and ethically	Yes

appropriate), referencing such data using a unique identifier in the references and in the “Availability of Data and Materials” section of your manuscript.<br><br>Have you have met the above requirement as detailed in our Minimum Standards Reporting Checklist?	

 Click here to access/download;Manuscript;Revised manuscript-GIGA-D-21-00190.docx

A high-quality assembly reveals genomic characteristics, phylogenetic status and causal genes for white feather of Indian peafowl

Shaojuan Liu[1#], Hao Chen[3#], Jing Ouyang[3], Min Huang[1], Hui Zhang[1], Sumei Zheng[1], Suwang Xi[2], Hongbo Tang[3], Yuren Gao[3], Yanpeng Xiong[3], Di Cheng[2], Kaifeng Chen[2], Bingbing Liu[1],Wanbo Li[4], Xueming Yan[3*], Huirong Mao[2*], Jun Ren [1*]

[1] College of Animal Science, South China Agricultural University, Guangzhou 510642, China

[2] School of Animal Science and Technology, Jiangxi Agricultural University, Nanchang 330045, China

[3] College of Life Science, Jiangxi Science & Technology Normal University, Nanchang 330013, China

[4] Key Laboratory of Healthy Mariculture for the East China Sea, Ministry of Agriculture and Rural Affairs, Jimei University, Xiamen 361021, China

[#] Both authors contribute equally to this paper.

[*] Corresponding author.

E-mail address: maohuirong82@hotmail.com (H. M); xuemingyan@hotmail.com (X. Y)

Abstract

Background: The dazzling phenotypic characteristics of male Indian peafowl (*Pavo cristatus*) are attractive to both the female of the species and to humans. However, little is known about the evolution of the phenotypic and phylogeny of these birds at the whole-genome level. So far, there are no reports regarding the genetic mechanism of the formation of white feathers in this variant of Indian peafowl.

Results: A draft genome of Indian peafowl was assembled, with a genome size of 1.05 Gb (the sequencing depth is 362×), and contig and scaffold N50 were up to 6.2 Mb and 11.4 Mb, respectively. Compared with other birds, Indian peafowl showed changes in terms of metabolism, immunity, skeletal development and feather development, which provided a novel insight into the phenotypic evolution of peafowl, such as the large body size and feather morphologies. Moreover, we determined that the phylogeny of Indian peafowl was more closely linked to turkey than chicken. Specifically, we first identified that *PMEL* was a potential causal gene leading to the formation of the white feather variant in Indian peafowl.

Conclusions: This study provides an Indian peafowl genome of high-quality as well as a novel understanding of phenotypic evolution and phylogeny of Indian peafowl. These results provide a valuable reference for the study of avian genome evolution. Furthermore, the discovery of the genetic mechanism for the development of white feathers is both a breakthrough in the exploration of peafowl plumage, and also offers clues and directions for further investigations of the avian plumage coloration and artificial breeding in peafowl.

Keywords: Indian peafowl; Genome assembly; Phylogeny; *PMEL*; White feather

Introduction

Pavo cristatus, commonly called the Indian peafowl or blue peafowl, represents elegance, honour, beauty, luck and romance in many Asian cultures (Figure 1a) (Gadagkar, 2003; Kushwaha and Kumar, 2016). Peafowl, belongs to Aves, Galliformes, Phasianidae, *Pavo*, and has two species: green peafowl and blue peafowl. The Indian peafowl is the national bird of India and is widely distributed in Bangladesh, Bhutan, India, Nepal, Pakistan, and Sri Lanka (Kushwaha and Kumar, 2016; Ramesh and McGowan, 2009). Indian peafowl has exclusive characteristics, even the Phasianidae family; for instance, it has a larger body size, fan-shaped crests, glittering plumage and an iridescent tail, and is of great ornamental value. These qualities have attracted scientific and research attention. Moreover, many studies have suggested that Indian peafowl is a protein resource with high nutritional values, including its meat, internal organs and bones; furthermore, it has medicinal value and is therefore widely bred in many countries (Mushtaq-ul-Hassan et al., 2012; Paranjpe and Dange, 2019; Talha et al., 2018).

With the improvement of whole genome sequencing technology, an increasing number of avian genomes are being assembled, such as *Numida meleagris* (Shen et al., 2021), *Phasianus colchicus* (He et al., 2021) and *Fringilla coelebs* (Recuerda et al., 2021), which provide basic references for the study of phenotypic characteristics, evolution, economic traits and environmental adaptation of birds (Feng et al., 2020). Comparative genomics analysis is an important tool for revealing the adaptive evolution, phenotypic evolution, and genome characteristics of species (Alföldi and Lindblad-Toh, 2013), and it is widely applied to studies of the evolution and origin of animals or plants (Huang et al., 2020; Lin et al., 2019; Zhang et al., 2014). The first draft of Indian peafowl

genome assembly was released in 2018. However, the length of scaffold and contig N50 of the assembly were only 25.6 kb and 19.3 kb, respectively (Jaiswal et al., 2018). Subsequently, Dhar et al. improved the Indian peafowl genome using Illumina and Oxford Nanopore technology (ONT), and the length of scaffold N50 was determined up to 0.23 Mb (Dhar et al., 2019); however, the assembly quality still needed improvement. Additionally, previous studies of Indian peafowl were mainly focused on courtship behaviour (Dakin et al., 2016), immunity (Wang et al., 2019) and productivity (Samour et al., 2010; Shen et al., 2014). Some studies regarding the phylogeny of Indian peafowl have been based on the mitochondrial genome, DNA transposable factors and partial DNA nucleotide sequences, but few reports have addressed the whole genome level (Naseer et al., 2018; Shen et al., 2014; Zhou et al., 2015). Therefore, an improved genome of the Indian peafowl is needed to provide baseline data for further studies on this species, including genomic characteristics, and adaptive and phenotypic evolution.

Avian plumage is colourful and attractive; it has functions in protection, courtship, signal identification and provides an excellent model for the exploration of plumage formation, behaviour and phenotypic evolution in animals. Interestingly, studies on Indian peafowl plumage colour report that there are many plumage colour mutants, including white, black, variegated, cameo and oaten (Ouyang et al., 2009; Somes and Burger, 1993; Somes and Burger, 1991), among which, the most ornamental colour is the white plumage, caused by leucism rather than albinism, since the feather is white but the eyes contain melanin pigmentation (Figure 1b). The inherited basis of plumage colour has attracted researchers for a long time. The first reports suggested that the plumage phenotype of peafowl was determined by autosomal genes in a recessive model (Somes and Burger, 1991). A later study verified that a single autosomal locus

was in control of all plumage phenotypes in peafowl, where the pied colour appeared in two heterozygous mutant alleles, with black on the recessive mutant allele and the all-white plumage on the homozygous mutant allele as the most dominant (Somes and Burger, 1993). Nevertheless, further studies on the genetic mechanism of the white plumage in peafowl were needed to clarify the causative mutations of this phenotype.

A high-quality (near-chromosomal) reference genome of the Indian peafowl was constructed using third-generation de novo assembly technology. Based on the assembly, comparative genomics analysis was performed to investigate the biological characteristics of evolution at the genome-wide level through comparing the Indian peafowl genome with the high-quality genomes of other birds, humans and the mouse. Furthermore, transcriptomic and pooled resequencing data were analysed to identify the genetic mechanism of the white plumage variant in Indian peafowl. This work will provide an updated understanding and key reference for genomic characteristics, adaptive and phenotypic evolution and the genetic mechanism of the white feather trait in Indian peafowl.

Materials and Methods

Sample collection

All procedures used for this study and involved in animals were fully complied with guidelines for the care and utility of experimental animals established by the Ministry of Agriculture of China. The ethics committee of South China Agricultural University approved this study. A blood sample was collected from a female Indian peafowl for genome assembly and 51 blood samples from 35 blue feather peafowls and 16 white feather peafowls for pooled resequencing in Leping Sentai special breeding Co., Ltd in Jiangxi Province, China, under the principles and standards of animal welfare ethics.

Meanwhile, two liver and two muscle tissues were sampled from a female Indian peafowl to assist the process of genome assembly. Additionally, feather pulps from 8 blue and 8 white peafowls were collected for RNA-seq.

DNA and RNA extraction

Genomic DNA was extracted from blood samples using a routine phenol-chloroform protocol. The concentration of the extracted DNA was evaluated using a Nanodrop 2000 spectrophotometer (Thermo Fisher Scientific, Waltham, MA, USA), and diluted to a final concentration of 100 ng/μL. The integrity of DNA was checked via electrophoresis on 0.8% agarose gel. Total RNA of feather pulp was extracted using TRIzol reagent (Thermo Fisher Scientific, Waltham, MA, USA). The purity and degradation of RNA was detected by Nanodrop 2000 spectrophotometer and agarose gel electrophoresis.

De novo assembling of the Indian peafowl reference genome

Library Preparation and Sequencing: Genomic DNA was used to make a 350 bp insert fragment libraries using Illumina TruSeq Nano method, starting with 100 ng DNA. Mate pair libraries were made by Nextera Mate Pair Sample Preparation Kit (Illumina) with the gel plus option, and sequenced using Illumina NovaSeq 6000 platform. For PacBio sequencing, genomic DNA was sheared by a g-TUBE device (Covaris) with 20 kb settings for further preparing a 20 kb Single-Molecule Real Time (SMRT) bell, and then the single-molecule sequencing was completed on a PacBio RS-II platform. For 10× genomics sequencing, each GEM was amplified by PCR and added P7 sequencing adapters for Illumina sequencing.

Genome Assembly: The genome assembly of Indian peafowl was performed in five steps, which was illustrated in Supplementary Figure S1. The raw reads were generated from two paired-end libraries sequenced on Illumina NovaSeq 6000 platform. The sequencing adapters, contaminated reads, and low-quality reads were removed using megablast v2.2.26 (Chen et al., 2015). The genome size was calculated by the formula: Genome size = kmer_Number / Peak_Depth. Secondly, PacBio sequencing was used to control and correct errors. The error corrected data were assembled by falcon software (Chin et al., 2016), and the Overlap-Layout-Consensus algorithm was used to obtain the consensus sequences, which were then corrected by quiver software (Chin et al., 2013). Combined with the second-generation sequencing data, the consensus sequences were recalibrated using the pilon software (Walker et al., 2014) to improve the accuracy, and high-quality consensus sequences were obtained. Thirdly, the 10× Genomics sequencing was used to assist the genome assembly. The 10× Genomics library was sequenced to obtain linked-reads, which were aligned to the consensus sequences obtained from the PacBio sequencing assembly, and then linked reads were added to assemble the super-scaffolds by fragScaff software (Adey et al., 2014). Fourthly, similar to the third step, Chicago sequencing data was used to assist the mapping of draft genome assembly. Finally, the Illumina reads were mapped to the draft genome using BWA (Burrows-Wheeler Aligner) (Li and Durbin, 2009). Then, pilon (version 1.22) was used to correct the assembled errors based on the mapped results.

Consistency and completeness: The consistency and integrity of assembled peafowl genome were separately assessed using the BUSCO (Benchmarking Universal Single-Copy Orthologs) (Simão et al., 2015) and CEGMA (Core Eukaryotic Genes Mapping

Approach) (Parra et al., 2007; Parra et al., 2009), based on single-copy orthologues from the AVES (odb9) database. In order to evaluate the accuracy, integrity and sequencing uniformity of the genome assembly, small fragment library reads were selected and aligned to the assembled genome using BWA software. All the genomic sequences were generated by Novogene Inc, Beijing, China.

Genome Annotation: Genome annotation mainly included three aspects: repetitive sequence annotation, gene annotation (including gene structure prediction and gene function prediction) and non-coding RNA (ncRNA) annotation (Supplementary Figure S2). The repetitive sequence annotation included the annotation through homologous sequence alignment and ab initio prediction. The RepeatMasker and RepeatproteinMask software (Tempel, 2012) were employed to identify known repetitive sequences against the RepBase library (Jurka et al., 2005). In ab initio prediction, LTR_FINDER (Xu and Wang, 2007), RepeatScout (Price et al., 2005), and RepeatModeler (Flynn et al., 2020) were used to establish the de novo repeat sequence library, and then repetitive sequences were predicted by Repeatmasker software. The Tandem Repeats (TEs) in the genome were found by Tandem Repeat Finder software (Benson, 1999). In gene annotation, it mainly combined three prediction methods: homology-based prediction, de novo prediction, and other evidence-backed predictions. Homology-based prediction used the protein sequences of Gallus gallus, Meleagris gallopavo, Peking duck, Struthio camelus, Nipponia nippon, and Eastern Zhejiang white goose, downloaded from Ensembl (release 74), to align to the Indian peafowl genome using TblastN (Kent, 2002). Genewise (Birney et al., 2004) was used to align to the matched proteins for a precise gene model.

In addition, Augustus (Stanke et al., 2006), GlimmerHMM (Majoros et al., 2004), Geneid (Alioto et al., 2018), GenScan (Burge and Karlin, 1997), and SNAP software (Korf, 2004) were used for the ab initio predictions of gene structures. The above predictions with transcriptome-based data being combined, EVidenceModeler software (Haas et al., 2008) was used to integrate the gene set and generate a non-redundant and more complete gene set. Finally, PASA was used to correct the annotation results of EVidenceModeler for the final gene set. Gene function of the final gene set was annotated using the protein database of SwissProt (Bairoch and Apweiler, 2000), NR (O'Leary et al., 2016), Pfam (El-Gebali et al., 2019), KEGG (Kanehisa and Goto, 2000), and InterPro (Zdobnov and Apweiler, 2001). tRNAscan-SE software (Lowe and Eddy, 1997) was used to search for the tRNA sequence of genome, with INFERNAL software (http://infernal.janelia.org/) (Nawrocki and Eddy, 2013) from Rfam (Griffiths-Jones et al., 2005) to predict miRNA and snRNA of genome.

Gene family

The amino acid sequences of the following were downloaded from NCBI database to identify the gene families and single-copy orthologous genes. They are: Japanese quail (*Coturnix japonica*) (Nishibori et al., 2001), chicken (*Gallus gallus*) (Bellott et al., 2017), turkey (*Meleagris gallopavo*) (Dalloul et al., 2010), northern bobwhite (*Colinus virginianus*) (Oldeschulte et al., 2017), common mallard (*Anas platyrhynchos*) (Gregory and James, 2014), zebra finch (*Taeniopygia guttata*) (Korlach et al., 2017), collared flycatcher (*Ficedula albicollis*) (Ellegren et al., 2012), medium ground-finch (*Geospiza fortis*) (Zhang et al., 2014), tibetan ground-tit (*Pseudopodoces humilis*) (Cai

et al., 2013), rock pigeon (*Columba livia*) (Shapiro et al., 2013), peregrine falcon (*Falco peregrinus*) (Zhan et al., 2013), saker falcon (*Falco cherrug*) (Friedman-Einat et al., 2014), human (*Homo sapiens*) (Mohajeri et al., 2016), and mouse (*Mus musculus*) (Church et al., 2011). The longest transcript of each gene was extracted and then the genes with the length of protein sequences shorter than 50 amino acids were filtered. Based on the filtered protein-coding sequences data set, Orthofinder v2.3.7 (Yu et al., 2011) was used to identify gene families and orthologous gene clusters of 15 species. The single-copy orthologous sequences from the gene families were aligned using MAFFT v7.450 software (Katoh and Standley, 2013), and then the poorly sequences were removed using Trimal software with default parameters (Capella-Gutiérrez et al., 2009). The final result was used as a single data set for the subsequent comparative genome analyses.

Phylogenetic tree and divergence time

To determine the phylogenetic relationship of 15 species, IQ-tree v2.1.2 software was first used to find the best model for constructing phylogenetic tree with options "-m MF" and the species tree with bootstrap 1000 based on the concatenated alignment of single-copy orthologues sequences from 15 species (Minh et al., 2020). RAxML software was used to construct phylogenetic tree with parameters "-m PROTGAMMALGX -f a" with bootstrap 1000. Divergence time of 15 species was estimated by using MCMCtree program implemented in PAML packages (Yang, 2007). Five calibration time (human-mouse (85~97Mya), human-zebra finch (294~323Mya), zebra finch-medium ground finch (30.4~46.8Mya), common mallard-zebra finch (93.2~104.6Mya) and saker falcon-peregrine falcon (1.66~3.68Mya)) from TimeTree database (Hedges et al., 2006) were used as constrains in the divergence time estimation.

The MCMC process was run to sample 1,000,000 times, sample frequency set to 10, and burn-in 40,000, to finally achieve a convergence until the value of efficient sampling size (ESS) greater than 200 using Tracer v1.7.1 (http://tree.bio.ed.ac.uk/software/tracer/).

Genome Synteny and Collinearity Analysis

To compare the genome synteny of peafowl with chicken and turkey, the homologue of the genome was identified using BLASTp (E-value < $1e^{-10}$). Gene pairs of synteny blocks within the genome were identified using MCScanX (Wang et al., 2012), and the synteny blocks were showed by circos program from TBtools (Chen et al., 2020). To estimate the positively selected genes for peafowl-chicken and peafowl-turkey, the value of Ka/Ks (ω) for each gene pair was calculated by KaKs_calculator (Zhang et al., 2006), and the density curve of values was visualized by R software. The positively selected genes (ω > 1) were conducted based on functional enrichment analysis.

Gene-family expansion and contraction

To identify the gene family expansion and contraction in peafowl, the gene families in 15 species and phylogenetic tree with divergent times were taken into account to estimate the significance of gene gain and loss in gene family using the CAFE v4.2.1 with a random birth and death model and significance of P-values < 0.05 (De Bie et al., 2006). The parameter λ represents the probability of gene gain and loss in a divergent time. In order to investigate the evolutionary rates of different branches of the tree, the argument with "-t" was used to define three different branches for 15 species: the first branch included mouse and human, the second branch was the Phasianidae, and other

birds were regarded as the third branch. Then, they were conjunct with the "-s" option to search the optimal λ value for different branches using the maximum likelihood.

Positive Selection Analyses

To determine the adaptive evolution under the positive selection in peafowl, the single-copy orthologous protein sequences shared among the 11 species (peafowl, chicken, turkey, common mallard, zebra finch, collared flycatcher, medium ground-finch, tibetan ground-tit, rock pigeon, peregrine falcon and saker falcon) were searched, filtered, and then converted to coding gene sequence (CDS) using EMBOSS backtranseq program (Rice et al., 2000). The CDS were aligned to codon by using PRANK with the option "-codon" (Löytynoja, 2014). The above alignments were analysed by CODEML program of the PAML package 4.9 (Yang, 2007). A branch-site model (TEST-II) (model = 2, NSsites = 2) was conducted to identify the positively selected genes of peafowl. The model assumed that a particular branch (foreground, alternative hypothesis) had a different ω value from all the sites compared to all other branches (background, null hypothesis), suggesting that positive selection occurred at only a few sites on a particular branch (foreground) (Yang, 2007). The peafowl was regarded as a foreground branch and other species as a background branch. Additionally, the branch model was used to identify the rapidly evolving genes in peafowl, assuming that the branch of peafowl was an alternative hypothesis (model = 2) and the branches of other species were the null hypothesis (model = 0). The dN/dS (ω) values between foreground branch and background branch were estimated using Likelihood Ratio Test (LRT) values based on chi-square test. When the ω value in the foreground branch was greater than the background branch, it suggested that the genes of the foreground branch were under positive selection ($P < 0.05$) and the positively selected sites were

determined using the Bayesian Empirical Bayes method. All the positively selected genes were performed on functional enrichment analysis using KOBAS (Xie et al., 2011).

Whole-genome resequencing and variants calling

The genomic DNA from 35 blue feather peafowls and 16 white feather peafowls were pooled, respectively. Then 1.5 μg DNA per pool was used for constructing the sequencing libraries using Truseq Nano DNA HT Sample preparation Kit (Illumina, USA) following manufacturer's constructions. Each pooled DNA was fragmented through sonication to a size of 350bp and end repaired, A-tailed, and ligated with the full-length adapter for Illumina sequencing with further PCR amplification. PCR-amplified sequencing libraries were purified (AMPure XP system) and analysed for size distribution on Agilent2100 Bioanalyzer, and were quantified using real-time PCR. These libraries constructed above were sequenced on an Illumina NovaSeq platform and 150bp paired-end reads were generated with insert size around 350 bp. The raw data were filtered by removing reads with ≥10% unidentified nucleotides (N), reads with > 50% bases having phred quality < 5, and reads with > 10 nt aligned to the adapter allowing ≤10% mismatches. The clean reads were mapped to the assembled reference genome using BWA with parameters "mem -t 4 -k 32 –M –R". Alignment files were converted to BAM files using SAMtools software (settings: –bS –t) (Li et al., 2009). In addition, potential PCR duplications were removed using SAMtools command "rmdup". Single nucleotide polymorphisms (SNPs) and insertions/ deletions (Indels) (< 50 bp) were detected using Genome Analysis Toolkit v 4.0 (GATK) pipeline (McKenna et al., 2010).

RNA sequencing (RNA-seq) on PacBio platform

The cDNA of feather was acquired through PrimeScript™ RT reagent Kit with gDNA Eraser (TaKaRa Bio. Inc, Dalian, China) according to the manufacturer's instructions. The cDNA was performed damage repair, end repair, SMRT (single-molecule, real-time) dumbbell-shaped adapters, and ligation of the adapters to construct a mixed library. Primers and DNA polymerase were then combined to form a complete SMRT bell library. The qualified library was used for sequencing on a PacBio Sequel platform. The clean data were aligned to the reference genome of Indian peafowl by STAR v2.5.3a (Dobin et al., 2013). The Transcript assembly and gene expression levels were conducted by StringTie v1.3.3 (Pertea, et al., 2015) and featureCounts (Liao, et al., 2014) in Subread software (Liao et al., 2013). Differentially expressed genes (DEGs) between blue and white feather were identified through DESeq2 (Love, et al., 2014) in condition of fold change >2 and P-value < 0.01. Subsequently, the functional enrichment analyses of DEGs were annotated through GO (Gene Ontology) (Ashburner et al., 2000) and KEGG (Kyoto Encyclopedia of Genes and Genomes) database.

cDNA amplification

cDNA of feathers was reversely transcribed with PrimeScript™ RT reagent Kit with gDNA Eraser (TaKaRa). The reverse transcription quantitative PCR (RT-qPCR) was conducted in a total volume of 10 µl including 5 µl SYBR Taq II kit (TaKaRa), 0.3 µl Rox Reference Dye (50x), 2.7 µl distilled water, 1 µl cDNA and 1 µl primers, and performed on a 7900HT RT-qPCR system (ABI). β-actin was selected as the internal reference gene. All primer sequences were shown in Supplementary Table S20.

Results

Genomic characteristics of Indian peafowl

Third-generation PacBio single-molecule real-time sequencing technology and second-generation Illumina sequencing technology were used and combined with 10× genomics to assemble the Indian peafowl genome. We obtained a sequencing volume of 164.03 Gb using an Illumina NovaSep 6000 platform, 112.57 Gb of sequencing data on a 10× Genomics sequencing platform and 110.74 Gb of sequencing data using the PacBio sequencing platform (Supplementary Table S1). In total, 387.34 Gb of sequencing data and a total coverage of 362× was obtained from the three sequencing strategies with the lengths of scaffold N50 and contig N50 separately up to 6.2 Mb and 11.4 Mb, respectively, which exhibited a 446-fold and 50-fold improvement in the scaffold N50 compared to the previously published Indian blue peafowl genome reported by Jaiswal et al. (Jaiswal et al., 2018) and Dhar et al. (Dhar et al., 2019) (Figure 2, Table 1 and Supplementary Table S2). The distribution of 17-kmer showed a major peak at 154× (Supplementary Figure S3). The Indian peafowl genome size was estimated to be 1.05 Gb. Current peafowl assembly was anchored into 726 scaffolds and guanine-cytosine (GC) content was 42.03% with a normal ratio of A, T, G and C (Figure 2 and Supplementary Table S2 - S3).

We assessed the completeness and base accuracy of Indian peafowl genome assembly using CEGMA and BUSCO. On the one hand, assembly of the draft genome presented a high mapping rate (98.05%) and coverage rate (99.87%) and low homozygous SNP rate (0.0002%) by mapping to the short reads, generally reflecting the high accuracy of genome assembly (Supplementary Table S4-S5). On the other hand, the BUSCO results showed that 88.71% of 248 core genes selected from six eukaryotic model organisms were covered. Additionally, 97.4% complete genes (including 96.8% complete and single-copy genes and 0.6% complete and duplicated

genes) were predicted, and 1.7% fragmented genes and 0.9% missing genes were identified from 2586 genes in the Aves dataset (Supplementary Table S6). Collectively, these important indicators implied relatively high genome coverages and continuity for the Indian peafowl genome, providing an important resource for molecular breeding and evolutionary studies of peafowl.

According to the homologous alignment and ab initio prediction, the Indian peafowl genome comprised 15.20% non-redundant repeat sequences, including 1.27% tandem repeats, 14.12% transposable elements and 7.35% transposable element proteins (Supplementary Table S7). A total of 14.56% of transposable elements were identified after combined TEs, 0.70% of which were DNA transposons, 3.93% were long terminal repeats (LTRs), 0.01% were short interspersed nuclear elements (SINE), and 10.68% was the long interspersed nuclear elements (LINE) (Supplementary Figure S4 and Supplementary Table S7-S8). Altogether, 19 465 non-redundant protein-coding genes were predicted, of which 15,766 (81%) were annotated to function according to six public databases (Table 1 and Supplementary Table S9-S10). Additionally, 354 microRNAs (miRNAs), 308 transfer RNAs (tRNAs), 151 ribosomal RNAs (rRNAs) and 334 small nuclear RNAs (snRNAs) were also identified (Supplementary Table S11). Overall, this assembly had improved continuity, completeness and accuracy.

Gene families and phylogenetic relationship of 15 species

The protein sequences of 15 species were used to search the orthologues using OrthoFinder (Emms and Kelly, 2015). The results showed that a total of 18 038 orthogroups were identified in 15 species, of which 5999 single-copy orthologues were

shared among these species (Figure 3a). In addition, 93 gene families were specific to peafowl and 11 447 gene families were shared by peafowl and other Phasianidae (chicken, turkey and Japanese quail; Figure 3b). The peafowl species-specific gene families were mainly involved in immune response and biological process; for example, *FOXP3*, *FZD3*, and *TP53* participated in many immunological processes and played an important role in melanoma and bone homeostasis (Supplementary Table S12) (Fischer et al., 2019; Li et al., 2019; Thoenen et al., 2019).

We concatenated 5999 single-copy orthologues of 15 species and then aligned them to construct a phylogenetic tree with a bootstrap value of 1000 using the maximum likelihood method (Supplementary Figure S5 and S6). The results showed that the Galliformes order was clustered, within which the Phasianidae family formed a group. Moreover, peafowl were found to be closer to turkey than chicken in the Phasianidae family; these findings were inconsistent with those reported by Jaiswal et al (Jaiswal et al., 2018). We found that the relationship of chicken and quail was closer than turkey; white duck, belonging to the Anseriformes order, was closer to the Galliformes order (Figure 3c). Additionally, the divergence time of all species was estimated and calibrated through the divergence time between human and mouse, human and zebra finch, zebra finch and medium ground finch, common mallard and zebra finch, and saker falcon and peregrine falcon from the TimeTree database. The divergence between Galliformes and Anseriformes was estimated to be 81.2 million years ago (Mya). The divergence between the northern bobwhite and Phasianidae family was represented by the calibration point of northern bobwhite and turkey. The divergence between the peafowl and turkey was about 35.1 Mya, sharing a common ancestor with the chicken about 36.9 Mya (Figure 3c). However, divergence between chicken and Japanese quail

was estimated to be 34.7 Mya within the range of divergence (33.2~42.3 Mya) according to TimeTree (Cai et al., 2017), suggesting that the relationships between the common ancestor of peafowl and turkey, and chicken and Japanese quail were very close, as well as the relationship between these four species. The divergence of pheasant birds took place in the Tertiary era; this marks the advent of the modern biological era which was the peak period of divergence for animals and plants. At this time, new generation replaced the ancient types with an increase in the number of similar species, the common and diverse divergence of birds, and a rapid evolution of more species.

Genome synteny and collinearity among the Indian peafowl, chicken, and turkey

Collinearity analysis can reflect the homology of different species and genetic relationships. Genes with a pairwise ratio of nonsynonymous to synonymous substitutions (dN/dS) could be used to infer positive selection and contribute to understanding the evolutionary characteristics in species. In this study, pairwise synteny was compared between peafowl and chicken, and peafowl and turkey, and the ratio of dN/dS was calculated. Scaffold lengths greater than scaffold N70 (5 Mb) in the peafowl genome and other collinear scaffolds were marked as others were displayed (Figure 4a and 4b). Moreover, the distribution density of the dN/dS ratio was calculated and is showed in Figure 4c. Ninety-seven positively selected genes (dN/dS $>$ 1) in peafowl compared to chicken were associated with the biological process and immune-related pathways (*IL4*, *CD3D*, *CD3E* and *HLA-DMB*) ($P < 0.05$); for example, Th1 and Th2 cell differentiation, T cell receptor signaling pathway, and intestinal immune network for IgA production. Furthermore, compared with turkey, 43 positively selected genes were notebly enriched in GO terms of organelle (GO:0043226), extracellular space (GO:0005615), and epithelium migration (GO:0090132), and the pathways of

glutathione metabolism (*GPX1*, *GPX2* and *GPX4*) and thyroid hormone synthesis (*GPX1*, *DUOXA2* and *GPX2*) ($P < 0.05$) (Supplementary Table S13 and S14), which were involved in gastrointestinal health, anti-stress, growth development and metabolism. Notably, as a common positive selection gene, *EDN1* was reported to participate in many biological processes, such as epithelium migration and differentiation, pigmentation and their receptors (EDNRs) widely distributed in various tissues in chicken (Liu et al., 2019). These enrichment results indicated that the positively selected genes in peafowl were mainly related to intestinal immunity, anti-stress, growth development and metabolism, and pigmentation, compared with turkey and chicken in evolutionary process. These features were beneficial for peafowl to enhance adaptability, improve disease resistance and anti-stress ability, enrich plumage colour, and better adapt to the living environment during long-term artificial breeding.

Gene family expansion and contraction across the Indian peafowl genome

Likelihood analysis could identify the evolutionary rate and the notable expansion and contraction of gene families in species (De Bie et al., 2006). In this study, changes of gene family in peafowl were examined with a likelihood ratio test. Compared to the gene families in other species, the results suggested that 121 expansions and 2999 contractions of gene families ($P < 0.05$) were detected in peafowl (Figure 3c), of which, 21 significantly gained genes were mainly involved in energy metabolism and storage (*GIMAP1*, *GIMAP2* and *GIMAP8*) and immune response (*CD244*) ($P < 0.05$), such as the GO terms of natural killer cell activation involved in immune response (GO:0002323), MHC class I protein binding (GO:0042288), positive regulation of interleukin-8 production (GO:0032757), positive regulation of interferon-gamma production (GO:0032729), and lipid droplet (GO:0005811) (Supplementary Table S15).

Conversely, 23 significantly contracted genes were mainly relevant to biological processes such as fatty acid degradation (*ALDH3A2*) (GO:0001561), myocardium development (GO:0048739), muscle contraction and cardiac disease (*MYH6*, *MYH7* and *MYH7b*), olfactory receptor activity (*OR52B2*, *OR52K1* and *OR4S1*) (GO:0004984), and the pathways of olfactory transduction, metabolism and cardiac muscle contraction (Supplementary Table S16). For example, the expression of *MYH6* and *MYH7* directly dictated the slow- or fast-twitch phenotype in skeletal muscle and played a vital role in cardiomyocyte energetics and metabolism (Stuart et al., 2016; Toepfer et al., 2020). The olfactory genes were importantly characteristic during adaptive evolution in birds (Steiger et al., 2009). During their long-term domestication, peafowl have been artificially raised and fed a manufactured diet; as a result, their ability to find food and fly has declined, which likely caused the contraction of genes related to the sense of smell and the regulation of skeletal muscle movement. In addition, we observed that Phasianidae had a higher rate of birth and death than that of the other two branches, indicating that this family underwent a rapid evolution.

Positively selected genes in the Indian peafowl genome

To reveal the adaptive divergence and evolution of peafowl, positive selection was analysed by using the branch-site model in the CODEML program. Significantly positive sites were evaluated by Bayes Empirical Bayes values (BEB $\geq$ 0.95), which demonstrated that the sites were under positive selection in branch-site model A (foreground). In the branch of peafowl (foreground), 3417 genes were under significantly positive selection based on BEB values ($P < 0.05$). These genes were annotated and classified through the analysis of GO ontology and KEGG pathways in order to further explore the impact of adaptive evolution on peafowl. According to the

results of functional enrichment analyses, we briefly summarized that these positively selective genes mainly participated in the process of lipid metabolism (i.e., GO:0005811, GO:0030169, and GO:0008289), limb and skeletal development (i.e., GO:0060173, GO:0001503, and GO:0030509), immune response (i.e., GO:0070498, GO:0043123, and GO:1901224), pigmentation (GO:0042470 and GO:0030318), sensory perception (i.e., GO:0008542, GO:0008542, and GO:0007605), and other GO terms (Supplementary Table S17). Additionally, the pathways of positively selected genes were notably enriched in metabolic pathways, PI3K-Akt signaling pathway, NF-kappa B signaling pathway, pathways in cancer, MAPK signaling pathway, TNF signaling pathway, Jak-STAT signaling pathway, mTOR signaling pathway, FoxO signaling pathway, fatty acid metabolism, IL-17 signaling pathway, cholesterol metabolism, Th17 cell differentiation, and so on (Supplementary Table S18), which were mainly associated with immunity, energy metabolism, and cell growth and differentiation.

The branch model was used to identify a total of 10 rapidly evolving genes in peafowl, including *BCL7A*, *MEF2C*, *MED27*, *COPS7A*, *NMNAT2*, *SLC25A25*, *TNIP2*, *ETS1*, *CCDC6* and *GSG1L*. Functional enrichment showed that significant pathways included those pathways in cancer, nicotinate and nicotinamide metabolism, thyroid cancer, renal cell carcinoma, parathyroid hormone synthesis, secretion and action, thyroid hormone signalling pathway, apelin signalling pathway, fluid shear stress and atherosclerosis ($P < 0.05$). Significant GO terms were involved in melanocyte differentiation, skeletal muscle and bone development, immunity, and response to stress (Supplementary Table S19). Notably, *MEF2C* was involved in most GO terms and pathways and played a vital role in bone and muscle development, immunity, and

melanocyte differentiation; therefore, it may have been an important gene in the rapid evolution of peafowl (Liu et al., 2017; Tang et al., 2016; Trajanoska et al., 2019).

Genes with allele frequency between blue and white feathers in Indian peafowl

To localize the genomic region underlying plumage colour, the allele frequency between blue and white peafowl feathers was analysed. The clean data of two pooled resequencing were aligned to the assembled peafowl genome using the Samtools with option "mpileup", and filtered to calculate allele frequency differences using Population2 software (Kofler et al., 2011). The significance of allele frequency differences was estimated by Fisher's exact test. Up- and downstream of 50 Kb with a -log10 (P-value) >30 were extracted as potential candidate regions. As a result, we found that *EDNRB* in scaffold 196 and *PMEL* in scaffold 144 were significantly related to plumage pigmentation (Figure 5a). Additionally, based on RNA-seq data, 69 down-regulated genes and 52 up-regulated genes between blue and white peafowl were detected, of which 10 up-regulated genes (*TRYP1*, *TYR*, *PMEL*, *EDNRB*, *OCA2*, *SLC24A5*, *SOX10*, *MC1R*, *SLC45A2* and *TRPM1*) were associated with melanin deposition (Figure 5b). The functional enrichment of DEGs showed that the most significant pathway was enriched in the process of melanin synthesis ($P < 0.05$; Figure 5c). In order to further investigate differences in allele imbalance in DEGs, we used resequencing data to identify the allelic imbalance by calculating the allele frequency of 10 pigmentation-related genes in the blue and white peafowl and annotated the function of sites using snpEff software (Cingolani et al., 2012). An observation showed that only two differential sites were located in *PMEL* and one in *EDNRB*, but none of the differential sites were obviously functional mutations, such as missense mutations, splicing mutations, or nonsense mutations (Figure 5d). Collectively, overlapping with

these results based on resequencing and RNAseq data, we determined that the formation of white feathers was most likely related to the differential expression of *PMEL* and *EDNRB* in peafowl.

Candidate causative gene for the white feather phenotype in blue and white feather peafowl

To detect the *PMEL* and *EDNRB* transcripts in blue and white feather peafowl, we examined the RNA-seq data of *PMEL* and *EDNRB* using the integrative genomics viewer (IGV) application. The results indicated that there was no difference in the transcript of *EDNRB* in the two types of feather pulp (Supplementary Figure S7), suggesting that *ENDRB* was normally expressed in blue and white feather peafowl. Compared to the transcript of *PMEL* in blue peafowl, we found that this gene was hardly expressed in white feather peafowl (Figure 5e). Moreover, to further determine the mRNA expression of *PMEL* in white feather peafowl, reverse transcription quantitative PCR (RT-qPCR) of *PMEL* was conducted in blue and white feather peafowl (Supplementary Table S20). RNA samples were extracted from feather pulps and used for subsequent PCR. Surprisingly, we observed that the mRNA expression of *PMEL* in white feather peafowl was significantly reduced in comparison to that in blue feather peafowl (P = 0.013; Figure 5f), which was consistent with the results of RNA-seq data. Hence, we confirmed that *PMEL* was a strong candidate causative gene for the formation of white feathers in blue and white feather peafowl. Further investigations are needed regarding the mechanism for the downregulated expression of *PMEL* in white feather peafowl.

Discussion

With the development of sequencing technology, the reduction of sequencing costs and the improvement of assembly methods, an increasing number of genome sequence maps of various species have been published, making the whole genome sequencing an important method for conducting basic genetic research on species. Recently, many avian genomes have been assembled, providing excellent material from which to study the genetic mechanisms of evolution, behaviour and pathology. In this study, three sequencing strategies were combined to construct the Indian peafowl genome, and 1.05 Gb total draft genome sequence was obtained, with a sequencing depth of up to 362×. Moreover, the lengths of contig N50 and scaffold N50 were respectively achieved at 6.2 Mb and 11.4 Mb, which was close to the chromosomal level. Compared with other avian genomes and the draft genomes of peafowl assembled by Jaiswal et al. and Dhar et al. (Dhar et al., 2019; Jaiswal et al., 2018), the current Indian peafowl genome showed a notable improvement of assembly quality, including consistency, accuracy and integrity. This draft genome of peafowl was a considerable improvement in terms of the quality of genome assembly and a strongly supported the subsequent comparative genomic analysis.

In recent years, since the rapid development of genomics and the accumulation of genomic data, comparative genomics has become a research hotspot that can now explain biological functions and evolutionary characteristics at a genome-wide level. In particular, avian genomes are favoured for the investigation of adaptive evolution and species-specific biological characteristics by discovering novel genes and gene function through comparative genomics analysis. In this study, comparative genomics analysis was conducted on peafowl and other avian species to explore the unique biological characteristics of peafowl during evolution. First, the construction of

phylogenetic relationships is key and a basis for many comparative genomic analyses. Generally, most phylogenetic relationship of birds are constructed based on mitochondrial DNA, the cytochrome b gene, nuclear genes or a combination of these (Armstrong et al., 2001; Meng et al., 2008; Naseer et al., 2017). Meanwhile, many studies using different data types to construct the tree have shown that there are controversial uncertainties in the phylogenetic classification of birds and that more evidence is needed for verification. In this study, single copy homologous amino acid sequences from whole genome sequencing data were used to construct a phylogenetic tree of 15 species; results suggested that the position of peafowl was closer to that of turkey than to chicken. This result did not agree with previous studies and may have been caused by different strategies and data (Dhar et al., 2019; Jaiswal et al., 2018). Moreover, the divergent time estimated that the divergence between peafowl and turkey was near to the divergence time among chicken and the ancestors of peafowl and turkey, indicating that the relationship among them was very close. Notably, we observed that *FOXP3* and *TP53* were mainly species-specific genes of peafowl compared to other Phasianidae. *FOXP3* is necessary for the development of regulatory T lymphocytes and is essential for maintaining immune homeostasis and immune self-tolerance to environmental antigens by eliminating natural reactive T cells in the thymus and peripheral organs. Meanwhile, *FOXP3* plays an important role in bone and haematopoietic homeostasis, inflammatory bone loss diseases and abnormal bone weight, which can affect lymphoid haematopoiesis by acting on the development and function of osteoclasts (Fischer et al., 2019). *TP53* plays an important role in inhibiting the progression of bone and soft tissue sarcoma. The loss of *TP53* activity can promote the osteogenic differentiation of bone marrow stromal cells and the development of

osteosarcoma of these cells, which can prevent their malignant transformation (Thoenen et al., 2019). In this study, the enrichment of these genes specific to chicken and turkey in peafowl showed that a healthy development and immunity of bones was important in peafowl evolution, as this was conducive to achieving breeder's demand for rapid growth, large size, and strong disease resistance in domestication.

Species-specific immune-related genes are always positively selected in the adaptive evolution of many species. In this study, the number of GO terms and pathways related to immunity in peafowl was greater than that of others, such as the expansive genes and rapidly evolving genes involved in the process of MHC class I protein binding, TNF signalling pathway, NF-kappaB signalling, IL-17 signalling pathway and Th17 cell differentiation. Likewise, we found that many olfactory genes and myosin genes were lost in peafowl. Myosin is a functional and structural protein that directly regulates muscle contraction, movement and cardiac function in animals (Harrington and Rodgers, 1984). Olfaction plays a crucial role in avian life, which contributes to the recognition of food, courtship, or the detection of danger (Khan et al., 2015; Lu et al., 2016). Birds can recognize close relatives to avoid inbreeding and distinguish the direction of migration by using their acute sense of olfaction (Holland et al., 2009; Krause et al., 2012). However, peafowl in this study were artificially farmed and the manufactured feed supplied throughout domestication has caused a gradual degradation of their ability to find food in the wild and to fly, which may explain the loss of myosin family genes and olfactory family and contribute to inbreeding and a reduction of energy expenditure.

Most birds have a small body size is small owing to the pressure of body weight and reduction in energy expenditure (Blackburn and Gaston, 1994). However, the peafowl

is well known to have a large body size, huge tail, and beautiful plumage, all of which are likely to have gradually evolved owing to better adaption to ecological environment. In this study, the enrichment analysis of positive selection genes was mainly involved in skeletal development, bone morphology, energy metabolism and storage, such as the mTOR signalling pathway, MAPK signalling pathway, BMP signalling pathway, limb development, lipid droplet, and lipid binding. mTOR is a central integrator of cellular growth and metabolism and the mTOR signalling pathway plays a vital role in innate and adaptive immune responses and the regulation of energy balance (Jones and Pearce, 2017; Xu et al., 2012). BMP is an important member of the transforming growth factor-β (TGF-β) superfamily through regulating the activity of downstream genes to participate in many important biological processes, such as nervous system differentiation, tooth and bone development, and cancer (Buijs et al., 2007; Huang et al., 2018). The MAPK signalling pathway also participates in the regulation of feather growth and development (Fang et al., 2018). Moreover, as a rapidly evolving gene, we observed that *MEF2C* could regulate muscle and cardiovascular development and is not only a core component of development in regulating muscle, nerve, cartilage-like, immune and endothelial cells, but is also necessary for normal chondrocyte hypertrophy and ossification (Dong et al., 2017; Mackie et al., 2008). Cartilage formation is a key process in vertebrate bone development and health maintenance, and most bones are developed through cartilage ossification. Potthoff et al. suggest that *MEF2C* can directly regulate transcription of the myosin gene, and the loss of *MEF2C* in skeletal muscle causes improper sarcomere organization, which reveals the key role of *MEF2C* in maintaining sarcomere integrity and skeletal muscle maturation after birth (Potthoff et al., 2007). Arnold et al. indicate that the transcription factor *MEF2C* could regulate

muscle and cardiovascular development, and control skeletal development by activating the genetic program of chondrocyte hypertrophy (Arnold et al., 2007). Hence, in this study, we found that *MEF2C* underwent rapid evolution in peafowl and may be conducive to the development and morphology of bones and the maintenance of body shape. This may well explain the evolutionary phenotype characteristics of the increasing weight and body size of peafowl in order to meet breeder's needs during domestication. Furthermore, the iridescent plumage and long tail are also deeply attractive. Many positively selected genes associated with pigmentation, such as *TYR*, *SZT2*, *NF1*, *ARCN1*, *KIT*, *HPS5*, *FIG4*, *LYST*, *RACK1*, *USP13*, *HPS6*, *OCA2*, *MITF* and *BCL2* were also identified. All the above results contribute to understanding the phenotypic characteristics, such as large body size, long tail and dazzling plumage in peafowl, during evolutionary adaptation.

To date, a number of studies examining the genetic mechanism of plumage colour in avians have been reported (Domyan et al., 2014; Robic et al., 2019). In the present study, the mechanism behind the white plumage phenotype in peafowl was explored combining transcriptome analysis and RT-qPCR with resequencing data. Plumage colours are often determined by causal genes that may have a difference in allele frequency between different plumage colour populations. On the one hand, we used resequencing data to make selective signal analysis by detecting the allele frequency difference in blue and white peafowl results suggested that only *PMEL* and *EDNRB* were involved in pigmentation. On the other hand, we used RNA-seq data to determine the DEGs between blue and white peafowl, and results indicated that 10 significantly up-regulated genes were associated with melanin deposition. Subsequently, we used resequencing data to identify the allele imbalance difference sites of 10 up-regulated

genes and found that only *PMEL* and *EDNRB* had differential sites. Although there were many significant sites in the allele frequency difference, they did not cause differences in transcripts and resulted in the differential expression of related genes in the analysis of DEGs, with the exception of *PMEL* and *EDNRB*. By overlapping with the results based on resequencing and RNA-seq data, we determined that *PMEL* and *EDNRB* were candidate genes for the formation of white plumage in peafowl. Furthermore, we observed the transcripts of *PMEL* and *EDNRB* based on RNA-seq data by IGV visualization and discovered that *PMEL* was hardly expressed in white peafowl compared to blue peafowl; moreover, *EDNRB* was normally expressed in both variants. Finally, we verified the low mRNA expression of *PMEL* in white peafowl by RNT-qPCR; this results, suggested that *PMEL* was a strong candidate causative gene for the formation of white plumage. The formation and deposition of melanin mainly occurs on the amyloid fibres of melanosomes. As a key signal molecule, *PMEL* could directly initiate the formation of melanosomes and promote their synthesis (Watt et al., 2013). Moreover, many studies reported that mutations of *PMEL* could cause its low expression, leading to melanogenesis and further resulting in hypopigmentation phenotypes in animals like silver horses, white chicken and yellowish Japanese quail (Andersson et al., 2013; Ishishita et al., 2018; Kerje et al., 2004). Here, we detected that the low expression of *PMEL* was associated with white plumage in peafowl. However, in order to further investigate the causal mutations of *PMEL* low-expression, the mutations of *PMEL* were examined and annotated, but no functional mutation sites were found. We hypothesized that the low expression of *PMEL* transcription was probably caused by changes in regulatory elements located in the upstream 5 kb promoter region of *PMEL* and thus impeded melanin synthesis. Unfortunately, there

were no mutations in the core promoter region and transcription factor binding sites predicted by promoter prediction websites. In addition, resequencing data were also used to detect the structural variation of the *PMEL* gene and its upstream region. Moreover, the transcriptome data were used to detect SNP and Indel variation, as well as PCR amplification of the *PMEL* gene and its upstream 5 kb promoter region using Sanger sequencing; however, no possible variations were found. In view of these findings, we speculated that the *PMEL* gene was likely to exist as a complex structure as it could not be completely measured through sequencing and the causal sites were not identified; this needs to be further exploration. Nevertheless, for the first time to our knowledge, we identified that *PMEL* was a causal gene of white plumage, providing a novel insight into the formation of the white feather phenotype in blue and white feather peafowl. The results revealed the genetic mechanism of white plumage at the whole-genome transcriptome level.

Conclusion

This study performed an improvedassembly of higher quality and greater sequencing depth of the peafowl genome. First, the assembled genome is superior to two previous draft genomes of peafowl, both in terms of the sequencing depth and assembly quality. Secondly, based on the draft genome, the study determined that peafowl are closer to turkey than chicken at the genome-wide level. Moreover, the comparative genomic analysis indicated that the evolution of Indian peafowl metabolism, immunity, skeletal development and feather development that may be related to the unique characteristics of peafowl in domestication; this investigation was conducted to provide baseline information about the phenotypic evolution of peafowl. Finally, the study was the first to report a combination of resequencing and transcriptome analysis in Indian peafowl

and to reveal the molecular mechanism of white plumage formation. Altogether, the current study provided a novel reference genome of the systematic evolution of peafowl and other birds that can assist in understanding the formation of plumage colouration and suggests new theories for the artificial breeding of peafowl.

Data availability

The whole genome sequence data reported in this paper have been deposited in the Genome Warehouse in National Genomics Data Center (2020), Beijing Institute of Genomics (BIG), Chinese Academy of Sciences, under accession number GWHAZTP00000000 that is publicly accessible at https://bigd.big.ac.cn/gwh. The resequencing raw data has been deposited in the NCBI Sequence Read Archive (SRA) (https://submit.ncbi.nlm.nih.gov/subs/sra/) under accession number PRJNA665082. The transcriptomic raw data has been deposited in the NCBI under accession number PRJNA661158.

Author Contributions

X.Y., H.M. and J.R. designed the study and wrote the paper. S.L. and H.C. analyzed the data and wrote the paper. S.L., H.C., H.M. and W.L. revised the paper. S.L., H.Z. and B.L. conducted the validated experiments. S.L., J.O., M.H., S.Z., S.X., H.T., Y.G., Y.X., D.C., K.C., H.M. and Y.X. collected samples and performed the sequencing and genotyping experiments. All authors contributed and approved the final manuscript.

Funding

This work was supported by Educational Commission of Jiangxi Province of China (No. GJJ190177) and by the Key Research and Development Program of Jiangxi Province of China (No. 20171BBF60003).

Ethics approval and consent to participate

All procedures used for this study and involved in animals fully complied with guidelines for the care and utility of experimental animals established by the Ministry of Agriculture of China. The Animal Care and Use Committee of the South China Agricultural University approved this study.

Consent for publication

Not applicable.

Competing financial interests

The authors declare that they have no competing financial interests.

References

2020. Database Resources of the National Genomics Data Center in 2020. Nucleic acids research 48, D24-d33.

Adey, A., Kitzman, J.O., Burton, J.N., Daza, R., Kumar, A., Christiansen, L., Ronaghi, M., Amini, S., Gunderson, K.L., Steemers, F.J., Shendure, J., 2014. In vitro, long-range sequence information for de novo genome assembly via transposase contiguity. Genome research 24, 2041-2049.

Alföldi, J., Lindblad-Toh, K., 2013. Comparative genomics as a tool to understand evolution and disease. Genome research 23, 1063-1068.

Alioto, T., Blanco, E., Parra, G., Guigó, R., 2018. Using geneid to Identify Genes. Current protocols in bioinformatics 64, e56.

Andersson, L.S., Wilbe, M., Viluma, A., Cothran, G., Ekesten, B., Ewart, S., Lindgren, G., 2013. Equine multiple congenital ocular anomalies and silver coat colour result from the pleiotropic effects of mutant PMEL. PloS one 8, e75639.

Armstrong, M.H., Braun, E.L., Kimball, R.T., 2001. Phylogenetic Utility of Avian Ovomucoid Intron G: A Comparison of Nuclear and Mitochondrial Phylogenies in Galliformes. 118 %J The Auk, 799-804, 796.

Ashburner, M., Ball, C.A., Blake, J.A., Botstein, D., Butler, H., Cherry, J.M., Davis, A.P., Dolinski, K., Dwight, S.S., Eppig, J.T., Harris, M.A., Hill, D.P., Issel-Tarver, L., Kasarskis, A., Lewis, S., Matese, J.C., Richardson, J.E., Ringwald, M., Rubin, G.M., Sherlock, G., 2000. Gene ontology: tool for the unification of biology. The Gene Ontology Consortium. Nat Genet 25, 25-29.

Bairoch, A., Apweiler, R., 2000. The SWISS-PROT protein sequence database and its supplement TrEMBL in 2000. Nucleic acids research 28, 45-48.

Bellott, D.W., Skaletsky, H., Cho, T.J., Brown, L., Locke, D., Chen, N., Galkina, S., Pyntikova, T., Koutseva, N., Graves, T., Kremitzki, C., Warren, W.C., Clark, A.G., Gaginskaya, E., Wilson, R.K., Page, D.C., 2017. Avian W and mammalian Y chromosomes convergently retained dosage-sensitive regulators. Nat Genet 49, 387-394.

Benson, G., 1999. Tandem repeats finder: a program to analyze DNA sequences. Nucleic acids research 27, 573-580.

Birney, E., Clamp, M., Durbin, R., 2004. GeneWise and Genomewise. Genome research 14, 988-995.

Blackburn, T., Gaston, K., 1994. The Distribution of Body Sizes of the World's Bird Species. Oikos 70, 127-130.

Buijs, J.T., Henriquez, N.V., van Overveld, P.G., van der Horst, G., ten Dijke, P., van der Pluijm, G., 2007. TGF-beta and BMP7 interactions in tumour progression and bone metastasis. Clinical & experimental metastasis 24, 609-617.

Burge, C., Karlin, S., 1997. Prediction of complete gene structures in human genomic DNA. Journal of molecular biology 268, 78-94.

Cai, Q., Qian, X., Lang, Y., Luo, Y., Xu, J., Pan, S., Hui, Y., Gou, C., Cai, Y., Hao, M., Zhao, J., Wang, S., Wang, Z., Zhang, X., He, R., Liu, J., Luo, L., Li, Y., Wang, J., 2013. Genome sequence of ground tit Pseudopodoces humilis and its adaptation to high altitude. Genome Biol 14, R29.

Cai, T., Fjeldså, J., Wu, Y., Shao, S., Chen, Y., Quan, Q., Li, X., Song, G., Qu, Y., Qiao, G., Lei, F., 2017. What makes the Sino- Himalayan mountains the major diversity hotspots for pheasants? Journal of Biogeography.

Capella-Gutiérrez, S., Silla-Martínez, J.M., Gabaldón, T., 2009. trimAl: a tool for automated alignment trimming in large-scale phylogenetic analyses. Bioinformatics 25, 1972-1973.

Chen, C., Chen, H., Zhang, Y., Thomas, H.R., Frank, M.H., He, Y., Xia, R., 2020. TBtools: An Integrative Toolkit Developed for Interactive Analyses of Big Biological Data. Molecular plant 13, 1194-1202.

Chen, Y., Ye, W., Zhang, Y., Xu, Y., 2015. High speed BLASTN: an accelerated MegaBLAST search tool. Nucleic acids research 43, 7762-7768.

Chin, C.-S., Peluso, P., Sedlazeck, F.J., Nattestad, M., Concepcion, G.T., Clum, A., Dunn, C., O'Malley, R., Figueroa-Balderas, R., Morales-Cruz, A.J.N.m., 2016. Phased diploid genome assembly with single-molecule real-time sequencing. 13, 1050-1054.

Chin, C.S., Alexander, D.H., Marks, P., Klammer, A.A., Drake, J., Heiner, C., Clum, A., Copeland, A., Huddleston, J., Eichler, E.E., Turner, S.W., Korlach, J., 2013. Nonhybrid, finished microbial genome assemblies from long-read SMRT sequencing data. Nature methods 10, 563-569.

Church, D.M., Schneider, V.A., Graves, T., Auger, K., Cunningham, F., Bouk, N., Chen, H.C., Agarwala, R., McLaren, W.M., Ritchie, G.R., Albracht, D., Kremitzki, M., Rock, S., Kotkiewicz, H., Kremitzki, C., Wollam, A., Trani, L., Fulton, L., Fulton, R., Matthews, L., Whitehead, S., Chow, W., Torrance, J., Dunn, M., Harden, G., Threadgold, G., Wood, J., Collins, J., Heath, P., Griffiths, G., Pelan, S., Grafham, D., Eichler, E.E., Weinstock, G., Mardis, E.R., Wilson, R.K., Howe, K., Flicek, P., Hubbard, T., 2011. Modernizing reference genome assemblies. PLoS biology 9, e1001091.

Cingolani, P., Platts, A., Wang le, L., Coon, M., Nguyen, T., Wang, L., Land, S.J., Lu, X., Ruden, D.M., 2012. A program for annotating and predicting the effects of single

nucleotide polymorphisms, SnpEff: SNPs in the genome of Drosophila melanogaster strain w1118; iso-2; iso-3. Fly 6, 80-92.

Dakin, R., McCrossan, O., Hare, J.F., Montgomerie, R., Amador Kane, S., 2016. Biomechanics of the Peacock's Display: How Feather Structure and Resonance Influence Multimodal Signaling. PloS one 11, e0152759.

Dalloul, R.A., Long, J.A., Zimin, A.V., Aslam, L., Beal, K., Blomberg Le, A., Bouffard, P., Burt, D.W., Crasta, O., Crooijmans, R.P., Cooper, K., Coulombe, R.A., De, S., Delany, M.E., Dodgson, J.B., Dong, J.J., Evans, C., Frederickson, K.M., Flicek, P., Florea, L., Folkerts, O., Groenen, M.A., Harkins, T.T., Herrero, J., Hoffmann, S., Megens, H.J., Jiang, A., de Jong, P., Kaiser, P., Kim, H., Kim, K.W., Kim, S., Langenberger, D., Lee, M.K., Lee, T., Mane, S., Marcais, G., Marz, M., McElroy, A.P., Modise, T., Nefedov, M., Notredame, C., Paton, I.R., Payne, W.S., Pertea, G., Prickett, D., Puiu, D., Qioa, D., Raineri, E., Ruffier, M., Salzberg, S.L., Schatz, M.C., Scheuring, C., Schmidt, C.J., Schroeder, S., Searle, S.M., Smith, E.J., Smith, J., Sonstegard, T.S., Stadler, P.F., Tafer, H., Tu, Z.J., Van Tassell, C.P., Vilella, A.J., Williams, K.P., Yorke, J.A., Zhang, L., Zhang, H.B., Zhang, X., Zhang, Y., Reed, K.M., 2010. Multi-platform next-generation sequencing of the domestic turkey (Meleagris gallopavo): genome assembly and analysis. PLoS biology 8.

De Bie, T., Cristianini, N., Demuth, J.P., Hahn, M.W., 2006. CAFE: a computational tool for the study of gene family evolution. Bioinformatics 22, 1269-1271.

Dhar, R., Seethy, A., Pethusamy, K., Singh, S., Rohil, V., Purkayastha, K., Mukherjee, I., Goswami, S., Singh, R., Raj, A., Srivastava, T., Acharya, S., Rajashekhar, B., Karmakar, S., 2019. De novo assembly of the Indian blue peacock (Pavo cristatus) genome using Oxford Nanopore technology and Illumina sequencing. Gigascience 8, giz038.

Dobin, A., Davis, C.A., Schlesinger, F., Drenkow, J., Zaleski, C., Jha, S., Batut, P., Chaisson, M., Gingeras, T.R., 2013. STAR: ultrafast universal RNA-seq aligner. Bioinformatics 29, 15-21.

Domyan, E.T., Guernsey, M.W., Kronenberg, Z., Krishnan, S., Boissy, R.E., Vickrey, A.I., Rodgers, C., Cassidy, P., Leachman, S.A., Fondon, J.W., 3rd, Yandell, M., Shapiro, M.D., 2014. Epistatic and combinatorial effects of pigmentary gene mutations in the domestic pigeon. Current biology : CB 24, 459-464.

El-Gebali, S., Mistry, J., Bateman, A., Eddy, S.R., Luciani, A., Potter, S.C., Qureshi, M., Richardson, L.J., Salazar, G.A., Smart, A., Sonnhammer, E.L.L., Hirsh, L., Paladin, L., Piovesan, D., Tosatto, S.C.E., Finn, R.D., 2019. The Pfam protein families database in 2019. Nucleic acids research 47, D427-d432.

Ellegren, H., Smeds, L., Burri, R., Olason, P.I., Backström, N., Kawakami, T., Künstner, A., Mäkinen, H., Nadachowska-Brzyska, K., Qvarnström, A., Uebbing, S., Wolf, J.B., 2012. The genomic landscape of species divergence in Ficedula flycatchers. Nature 491, 756-760.

Emms, D.M., Kelly, S., 2015. OrthoFinder: solving fundamental biases in whole genome comparisons dramatically improves orthogroup inference accuracy. Genome Biol 16, 157-157.

Fang, G., Jia, X., Li, H., Tan, S., Nie, Q., Yu, H., Yang, Y., 2018. Characterization of microRNA and mRNA expression profiles in skin tissue between early-feathering and late-feathering chickens. BMC genomics 19, 399.

Feng, S., Stiller, J., Deng, Y., Armstrong, J., Fang, Q., Reeve, A.H., Xie, D., Chen, G., Guo, C., Faircloth, B.C., Petersen, B., Wang, Z., Zhou, Q., Diekhans, M., Chen, W., Andreu-Sánchez, S., Margaryan, A., Howard, J.T., Parent, C., Pacheco, G., Sinding, M.S., Puetz, L., Cavill, E., Ribeiro Â, M., Eckhart, L., Fjeldså, J., Hosner, P.A., Brumfield, R.T., Christidis, L., Bertelsen, M.F., Sicheritz-Ponten, T., Tietze, D.T., Robertson, B.C., Song, G., Borgia, G., Claramunt, S., Lovette, I.J., Cowen, S.J., Njoroge, P., Dumbacher, J.P., Ryder, O.A., Fuchs, J., Bunce, M., Burt, D.W., Cracraft, J., Meng, G., Hackett, S.J., Ryan, P.G., Jønsson, K.A., Jamieson, I.G., da Fonseca, R.R., Braun, E.L., Houde, P., Mirarab, S., Suh, A., Hansson, B., Ponnikas, S., Sigeman, H., Stervander, M., Frandsen, P.B., van der Zwan, H., van der Sluis, R., Visser, C., Balakrishnan, C.N., Clark, A.G., Fitzpatrick, J.W., Bowman, R., Chen, N., Cloutier, A., Sackton, T.B., Edwards, S.V., Foote, D.J., Shakya, S.B., Sheldon, F.H., Vignal, A., Soares, A.E.R., Shapiro, B., González-Solís, J., Ferrer-Obiol, J., Rozas, J., Riutort, M., Tigano, A., Friesen, V., Dalén, L., Urrutia, A.O., Székely, T., Liu, Y., Campana, M.G., Corvelo, A., Fleischer, R.C., Rutherford, K.M., Gemmell, N.J., Dussex, N., Mouritsen,

H., Thiele, N., Delmore, K., Liedvogel, M., Franke, A., Hoeppner, M.P., Krone, O., Fudickar, A.M., Milá, B., Ketterson, E.D., Fidler, A.E., Friis, G., Parody-Merino Á, M., Battley, P.F., Cox, M.P., Lima, N.C.B., Prosdocimi, F., Parchman, T.L., Schlinger, B.A., Loiselle, B.A., Blake, J.G., Lim, H.C., Day, L.B., Fuxjager, M.J., Baldwin, M.W., Braun, M.J., Wirthlin, M., Dikow, R.B., Ryder, T.B., Camenisch, G., Keller, L.F., DaCosta, J.M., Hauber, M.E., Louder, M.I.M., Witt, C.C., McGuire, J.A., Mudge, J., Megna, L.C., Carling, M.D., Wang, B., Taylor, S.A., Del-Rio, G., Aleixo, A., Vasconcelos, A.T.R., Mello, C.V., Weir, J.T., Haussler, D., Li, Q., Yang, H., Wang, J., Lei, F., Rahbek, C., Gilbert, M.T.P., Graves, G.R., Jarvis, E.D., Paten, B., Zhang, G., 2020. Dense sampling of bird diversity increases power of comparative genomics. Nature 587, 252-257.

Fischer, L., Herkner, C., Kitte, R., Dohnke, S., Riewaldt, J., Kretschmer, K., Garbe, A.I., 2019. Foxp3(+) Regulatory T Cells in Bone and Hematopoietic Homeostasis. Frontiers in endocrinology 10, 578.

Flynn, J.M., Hubley, R., Goubert, C., Rosen, J., Clark, A.G., Feschotte, C., Smit, A.F., 2020. RepeatModeler2 for automated genomic discovery of transposable element families. Proceedings of the National Academy of Sciences of the United States of America 117, 9451-9457.

Friedman-Einat, M., Cogburn, L.A., Yosefi, S., Hen, G., Shinder, D., Shirak, A., Seroussi, E., 2014. Discovery and characterization of the first genuine avian leptin gene in the rock dove (Columba livia). Endocrinology 155, 3376-3384.

Gadagkar, R., 2003. Is the peacock merely beautiful or also honest? Current Science 85, 1012-1020.

Gregory, M.K., James, M.J., 2014. Functional characterization of the duck and turkey fatty acyl elongase enzymes ELOVL5 and ELOVL2. The Journal of nutrition 144, 1234-1239.

Griffiths-Jones, S., Moxon, S., Marshall, M., Khanna, A., Eddy, S.R., Bateman, A., 2005. Rfam: annotating non-coding RNAs in complete genomes. Nucleic acids research 33, D121-124.

Haas, B.J., Salzberg, S.L., Zhu, W., Pertea, M., Allen, J.E., Orvis, J., White, O., Buell, C.R., Wortman, J.R., 2008. Automated eukaryotic gene structure annotation using EVidenceModeler and the Program to Assemble Spliced Alignments. Genome Biol 9, R7.

Harrington, W.F., Rodgers, M.E., 1984. Myosin. Annual review of biochemistry 53, 35-73.

He, C., Zhao, L., Xiao, L., Xu, K., Ding, J., Zhou, H., Zheng, Y., Han, C., Akinyemi, F., Luo, H., Yang, L., Luo, L., Yuan, H., Lu, X., Meng, H., 2021. Chromosome level assembly reveals a unique immune gene organization and signatures of evolution in the common pheasant. Molecular ecology resources 21, 897-911.

Hedges, S.B., Dudley, J., Kumar, S., 2006. TimeTree: a public knowledge-base of divergence times among organisms. Bioinformatics 22, 2971-2972.

Holland, R.A., Thorup, K., Gagliardo, A., Bisson, I.A., Knecht, E., Mizrahi, D., Wikelski, M., 2009. Testing the role of sensory systems in the migratory heading of a songbird. The Journal of experimental biology 212, 4065-4071.

Huang, L., Feng, G., Yan, H., Zhang, Z., Bushman, B.S., Wang, J., Bombarely, A., Li, M., Yang, Z., Nie, G., Xie, W., Xu, L., Chen, P., Zhao, X., Jiang, W., Zhang, X., 2020. Genome assembly provides insights into the genome evolution and flowering regulation of orchardgrass. Plant biotechnology journal 18, 373-388.

Huang, X., Zhong, L., Post, J.N., Karperien, M., 2018. Co-treatment of TGF-β3 and BMP7 is superior in stimulating chondrocyte redifferentiation in both hypoxia and normoxia compared to single treatments. Scientific reports 8, 10251.

Ishishita, S., Takahashi, M., Yamaguchi, K., Kinoshita, K., Nakano, M., Nunome, M., Kitahara, S., Tatsumoto, S., Go, Y., Shigenobu, S., Matsuda, Y., 2018. Nonsense mutation in PMEL is associated with yellowish plumage colour phenotype in Japanese quail. Scientific reports 8, 16732.

Jaiswal, S.K., Gupta, A., Saxena, R., Prasoodanan, V.P.K., Sharma, A.K., Mittal, P., Roy, A., Shafer, A.B.A., Vijay, N., Sharma, V.K., 2018. Genome Sequence of Peacock Reveals the Peculiar Case of a Glittering Bird. Front Genet 9, 392-392.

Jones, R.G., Pearce, E.J., 2017. MenTORing Immunity: mTOR Signaling in the Development and Function of Tissue-Resident Immune Cells. Immunity 46, 730-742.

Jurka, J., Kapitonov, V.V., Pavlicek, A., Klonowski, P., Kohany, O., Walichiewicz, J., 2005. Repbase Update, a database of eukaryotic repetitive elements. Cytogenetic and genome research 110, 462-467.

Kanehisa, M., Goto, S., 2000. KEGG: kyoto encyclopedia of genes and genomes. Nucleic acids research 28, 27-30.

Katoh, K., Standley, D.M., 2013. MAFFT multiple sequence alignment software version 7: improvements in performance and usability. Molecular biology and evolution 30, 772-780.

Kent, W.J., 2002. BLAT--the BLAST-like alignment tool. Genome research 12, 656-664.

Kerje, S., Sharma, P., Gunnarsson, U., Kim, H., Bagchi, S., Fredriksson, R., Schütz, K., Jensen, P., von Heijne, G., Okimoto, R., Andersson, L., 2004. The Dominant white, Dun and Smoky color variants in chicken are associated with insertion/deletion polymorphisms in the PMEL17 gene. Genetics 168, 1507-1518.

Khan, I., Yang, Z., Maldonado, E., Li, C., Zhang, G., Gilbert, M.T., Jarvis, E.D., O'Brien, S.J., Johnson, W.E., Antunes, A., 2015. Olfactory Receptor Subgenomes Linked with Broad Ecological Adaptations in Sauropsida. Molecular biology and evolution 32, 2832-2843.

Kofler, R., Pandey, R.V., Schlötterer, C., 2011. PoPoolation2: identifying differentiation between populations using sequencing of pooled DNA samples (Pool-Seq). Bioinformatics 27, 3435-3436.

Korf, I., 2004. Gene finding in novel genomes. BMC bioinformatics 5, 59.

Korlach, J., Gedman, G., Kingan, S.B., Chin, C.S., Howard, J.T., Audet, J.N., Cantin, L., Jarvis, E.D., 2017. De novo PacBio long-read and phased avian genome assemblies correct and add to reference genes generated with intermediate and short reads. Gigascience 6, 1-16.

Krause, E.T., Krüger, O., Kohlmeier, P., Caspers, B.A., 2012. Olfactory kin recognition in a songbird. Biology letters 8, 327-329.

Kushwaha, S., Kumar, A.J.J.W.R., 2016. A review on Indian peafowl (Pavo cristatus) Linnaeus, 1758. 4, 42-59.

Li, C., Nguyen, V., Clark, K.N., Zahed, T., Sharkas, S., Filipp, F.V., Boiko, A.D., 2019. Down-regulation of FZD3 receptor suppresses growth and metastasis of human melanoma independently of canonical WNT signaling. Proceedings of the National Academy of Sciences of the United States of America 116, 4548-4557.

Li, H., Durbin, R., 2009. Fast and accurate short read alignment with Burrows-Wheeler transform. Bioinformatics 25, 1754-1760.

Li, H., Handsaker, B., Wysoker, A., Fennell, T., Ruan, J., Homer, N., Marth, G., Abecasis, G., Durbin, R., 2009. The Sequence Alignment/Map format and SAMtools. Bioinformatics 25, 2078-2079.

Liao, Y., Smyth, G.K., Shi, W., 2013. The Subread aligner: fast, accurate and scalable read mapping by seed-and-vote. Nucleic acids research 41, e108.

Lin, Z., Chen, L., Chen, X., Zhong, Y., Yang, Y., Xia, W., Liu, C., Zhu, W., Wang, H., Yan, B., Yang, Y., Liu, X., Sternang Kvie, K., Røed, K.H., Wang, K., Xiao, W., Wei, H., Li, G., Heller, R., Gilbert, M.T.P., Qiu, Q., Wang, W., Li, Z., 2019. Biological adaptations in the Arctic cervid, the reindeer (Rangifer tarandus). Science 364.

Liu, C.F., Samsa, W.E., Zhou, G., Lefebvre, V., 2017. Transcriptional control of chondrocyte specification and differentiation. Seminars in cell & developmental biology 62, 34-49.

Liu, H., Luo, Q., Zhang, J., Mo, C., Wang, Y., Li, J., 2019. Endothelins (EDN1, EDN2, EDN3) and their receptors (EDNRA, EDNRB, EDNRB2) in chickens: Functional analysis and tissue distribution. General and comparative endocrinology 283, 113231.

Lowe, T.M., Eddy, S.R., 1997. tRNAscan-SE: a program for improved detection of transfer RNA genes in genomic sequence. Nucleic acids research 25, 955-964.

Löytynoja, A., 2014. Phylogeny-aware alignment with PRANK. Methods in molecular biology (Clifton, N.J.) 1079, 155-170.

Lu, Q., Wang, K., Lei, F., Yu, D., Zhao, H., 2016. Penguins reduced olfactory receptor genes common to other waterbirds. Scientific reports 6, 31671.

Majoros, W.H., Pertea, M., Salzberg, S.L., 2004. TigrScan and GlimmerHMM: two open source ab initio eukaryotic gene-finders. Bioinformatics 20, 2878-2879.

McKenna, A., Hanna, M., Banks, E., Sivachenko, A., Cibulskis, K., Kernytsky, A., Garimella, K., Altshuler, D., Gabriel, S., Daly, M., DePristo, M.A., 2010. The Genome Analysis Toolkit: a MapReduce framework for analyzing next-generation DNA sequencing data. Genome research 20, 1297-1303.

Meng, Y., Dai, B., Ran, J., Li, J., Yue, B., 2008. Phylogenetic position of the genus Tetraophasis (Aves, Galliformes, Phasianidae) as inferred from mitochondrial and nuclear sequences. Biochemical Systematics and Ecology 36, 626-637.

Minh, B.Q., Schmidt, H.A., Chernomor, O., Schrempf, D., Woodhams, M.D., von Haeseler, A., Lanfear, R., 2020. IQ-TREE 2: New Models and Efficient Methods for

Phylogenetic Inference in the Genomic Era. Molecular biology and evolution 37, 1530-1534.

Mohajeri, K., Cantsilieris, S., Huddleston, J., Nelson, B.J., Coe, B.P., Campbell, C.D., Baker, C., Harshman, L., Munson, K.M., Kronenberg, Z.N., Kremitzki, M., Raja, A., Catacchio, C.R., Graves, T.A., Wilson, R.K., Ventura, M., Eichler, E.E., 2016. Interchromosomal core duplicons drive both evolutionary instability and disease susceptibility of the Chromosome 8p23.1 region. Genome research 26, 1453-1467.

Mushtaq-ul-Hassan, M., Ali, Z., Arshad, M.I., Mahmood, S., Research, M.M.-u.-H.J.I.J.o.V., 2012. Effects of mating sex ratios in Indian peafowl (Pavo cristatus) on production performance at Wildlife Research Institute, Faisalabad (Pakistan). 13, 143-146.

Naseer, J., Anjum, K., Khan, W., Imran, M., Ishaque, M., Hafeez, S., Munir, M.A., Nazir, M.A., 2018. Phylogenetic analysis based studies on genetic variation of cytochrome B gene of Indian peafowl (Pavo cristatus) in Pakistan. Indian Journal of Animal Research 52, 343-346.

Naseer, J., Anjum, K.M., Khan, W.A., Imran, M., Ishaque, M., Hafeez, S., Munir, M.A., Nazir, M.A.J.I.J.o.A.R., 2017. Phylogenetic analysis based studies on genetic variation of Cytochrome b gene of Indian peafowl (Pavo cristatus) in Pakistan. 52, 343-346.

Nawrocki, E.P., Eddy, S.R., 2013. Infernal 1.1: 100-fold faster RNA homology searches. Bioinformatics 29, 2933-2935.

Nishibori, M., Hayashi, T., Tsudzuki, M., Yamamoto, Y., Yasue, H., 2001. Complete sequence of the Japanese quail (Coturnix japonica) mitochondrial genome and its genetic relationship with related species. Animal genetics 32, 380-385.

O'Leary, N.A., Wright, M.W., Brister, J.R., Ciufo, S., Haddad, D., McVeigh, R., Rajput, B., Robbertse, B., Smith-White, B., Ako-Adjei, D., Astashyn, A., Badretdin, A., Bao, Y., Blinkova, O., Brover, V., Chetvernin, V., Choi, J., Cox, E., Ermolaeva, O., Farrell, C.M., Goldfarb, T., Gupta, T., Haft, D., Hatcher, E., Hlavina, W., Joardar, V.S., Kodali, V.K., Li, W., Maglott, D., Masterson, P., McGarvey, K.M., Murphy, M.R., O'Neill, K., Pujar, S., Rangwala, S.H., Rausch, D., Riddick, L.D., Schoch, C., Shkeda, A., Storz, S.S., Sun, H., Thibaud-Nissen, F., Tolstoy, I., Tully, R.E., Vatsan, A.R., Wallin, C., Webb, D., Wu, W., Landrum, M.J., Kimchi, A., Tatusova, T., DiCuccio, M., Kitts, P., Murphy, T.D., Pruitt, K.D., 2016. Reference sequence (RefSeq) database at NCBI: current status, taxonomic expansion, and functional annotation. Nucleic acids research 44, D733-745.

Oldeschulte, D.L., Halley, Y.A., Wilson, M.L., Bhattarai, E.K., Brashear, W., Hill, J., Metz, R.P., Johnson, C.D., Rollins, D., Peterson, M.J., Bickhart, D.M., Decker, J.E., Sewell, J.F., Seabury, C.M., 2017. Annotated Draft Genome Assemblies for the Northern Bobwhite (Colinus virginianus) and the Scaled Quail (Callipepla squamata) Reveal Disparate Estimates of Modern Genome Diversity and Historic Effective Population Size. G3 (Bethesda, Md.) 7, 3047-3058.

Ouyang, Y.N., Yang, Z.Y., Da-Lin, L.I., Huo, J.L., Qian, K., Miao, Y.W.J.J.o.Y.A.U., 2009. Genetic Divergence between Pavo muticus and Pavo cristatus by Cyt b Gene.

Paranjpe, D., Dange, P.J.b., 2019. A tale of two species: human and peafowl interactions in human dominated landscape influence each others behaviour. 412254.

Parra, G., Bradnam, K., Korf, I., 2007. CEGMA: a pipeline to accurately annotate core genes in eukaryotic genomes. Bioinformatics 23, 1061-1067.

Parra, G., Bradnam, K., Ning, Z., Keane, T., Korf, I., 2009. Assessing the gene space in draft genomes. Nucleic acids research 37, 289-297.

Price, A.L., Jones, N.C., Pevzner, P.A., 2005. De novo identification of repeat families in large genomes. Bioinformatics 21 Suppl 1, i351-358.

Ramesh, K., McGowan, P., 2009. On the current status of Indian Peafowl Pavo cristatus (Aves: Galliformes: Phasianidae): keeping the common species common. Journal of Threatened Taxa 1, 106-108.

Recuerda, M., Vizueta, J., Cuevas-Caballé, C., Blanco, G., Rozas, J., Milá, B., 2021. Chromosome-Level Genome Assembly of the Common Chaffinch (Aves: Fringilla coelebs): A Valuable Resource for Evolutionary Biology. Genome biology and evolution 13.

Rice, P., Longden, I., Bleasby, A., 2000. EMBOSS: the European Molecular Biology Open Software Suite. Trends in genetics : TIG 16, 276-277.

Robic, A., Morisson, M., Leroux, S., Gourichon, D., Vignal, A., Thebault, N., Fillon, V., Minvielle, F., Bed'Hom, B., Zerjal, T., Pitel, F., 2019. Two new structural mutations in

the 5' region of the ASIP gene cause diluted feather color phenotypes in Japanese quail. Genetics, selection, evolution : GSE 51, 12.

Samour, J., Naldo, J., Rahman, H., Sakkir, M., 2010. Hematologic and plasma biochemical reference values in Indian peafowl (Pavo cristatus). Journal of avian medicine and surgery 24, 99-106.

Shapiro, M.D., Kronenberg, Z., Li, C., Domyan, E.T., Pan, H., Campbell, M., Tan, H., Huff, C.D., Hu, H., Vickrey, A.I., Nielsen, S.C., Stringham, S.A., Hu, H., Willerslev, E., Gilbert, M.T., Yandell, M., Zhang, G., Wang, J., 2013. Genomic diversity and evolution of the head crest in the rock pigeon. Science 339, 1063-1067.

Shen, Q.-K., Peng, M.-S., Adeola, A.C., Kui, L., Duan, S., Miao, Y.-W., Eltayeb, N.M., Lichoti, J.K., Otecko, N.O., Strillacci, M.G., Gorla, E., Bagnato, A., Charles, O.S., Sanke, O.J., Dawuda, P.M., Okeyoyin, A.O., Musina, J., Njoroge, P., Agwanda, B., Kusza, S., Nanaei, H.A., Pedar, R., Xu, M.-M., Du, Y., Nneji, L.M., Murphy, R.W., Wang, M.-S., Esmailizadeh, A., Dong, Y., Ommeh, S.C., Zhang, Y.-P., 2021. Genomic Analyses Unveil Helmeted Guinea Fowl (Numida meleagris) Domestication in West Africa. Genome biology and evolution 13.

Shen, Y.Y., Dai, K., Cao, X., Murphy, R.W., Shen, X.J., Zhang, Y.P., 2014. The updated phylogenies of the phasianidae based on combined data of nuclear and mitochondrial DNA. PloS one 9, e95786.

Simão, F.A., Waterhouse, R.M., Ioannidis, P., Kriventseva, E.V., Zdobnov, E.M., 2015. BUSCO: assessing genome assembly and annotation completeness with single-copy orthologs. Bioinformatics 31, 3210-3212.

Somes, R.G., Burger, R.E., 1993. Inheritance of the White and Pied Plumage Color Patterns in the Indian Peafowl (Pavo cristatus). 14, 53-55.

Somes, R.G., Burger, R.E.J.J.o.H., 1991. Plumage Color Inheritance of the Indian Blue Peafowl (Pavo Cristatus): Blue, Black-Shouldered, Cameo, and Oaten. 1.

Stanke, M., Keller, O., Gunduz, I., Hayes, A., Waack, S., Morgenstern, B., 2006. AUGUSTUS: ab initio prediction of alternative transcripts. Nucleic acids research 34, W435-439.

Steiger, S.S., Kuryshev, V.Y., Stensmyr, M.C., Kempenaers, B., Mueller, J.C., 2009. A comparison of reptilian and avian olfactory receptor gene repertoires: species-specific expansion of group gamma genes in birds. BMC genomics 10, 446.

Stuart, C.A., Stone, W.L., Howell, M.E., Brannon, M.F., Hall, H.K., Gibson, A.L., Stone, M.H., 2016. Myosin content of individual human muscle fibers isolated by laser capture microdissection. American journal of physiology. Cell physiology 310, C381-389.

Talha, M.M.H., Mia, M.M., Momu, J.M.J.I.J.o.D.R., 2018. Morphometric, productive and reproductive traits of Indian peafowl (Pavo cristatus) in Bangladesh. 8, 19039-19043.

Tang, R., Xu, X., Yang, W., Yu, W., Hou, S., Xuan, Y., Tang, Z., Zhao, S., Chen, Y., Xiao, X., Huang, W., Guo, W., Li, M., Deng, W., 2016. MED27 promotes melanoma growth by targeting AKT/MAPK and NF-κB/iNOS signaling pathways. Cancer letters 373, 77-87.

Tempel, S., 2012. Using and understanding RepeatMasker. Methods in molecular biology (Clifton, N.J.) 859, 29-51.

Thoenen, E., Curl, A., Iwakuma, T., 2019. TP53 in bone and soft tissue sarcomas. Pharmacology & therapeutics 202, 149-164.

Toepfer, C.N., Garfinkel, A.C., Venturini, G., Wakimoto, H., Repetti, G., Alamo, L., Sharma, A., Agarwal, R., Ewoldt, J.F., Cloonan, P., Letendre, J., Lun, M., Olivotto, I., Colan, S., Ashley, E., Jacoby, D., Michels, M., Redwood, C.S., Watkins, H.C., Day, S.M., Staples, J.F., Padrón, R., Chopra, A., Ho, C.Y., Chen, C.S., Pereira, A.C., Seidman, J.G., Seidman, C.E., 2020. Myosin Sequestration Regulates Sarcomere Function, Cardiomyocyte Energetics, and Metabolism, Informing the Pathogenesis of Hypertrophic Cardiomyopathy. Circulation 141, 828-842.

Trajanoska, K., Rivadeneira, F., Kiel, D.P., Karasik, D., 2019. Genetics of Bone and Muscle Interactions in Humans. Current osteoporosis reports 17, 86-95.

Walker, B.J., Abeel, T., Shea, T., Priest, M., Abouelliel, A., Sakthikumar, S., Cuomo, C.A., Zeng, Q., Wortman, J., Young, S.K., Earl, A.M., 2014. Pilon: an integrated tool for comprehensive microbial variant detection and genome assembly improvement. PloS one 9, e112963.

Wang, Y., Tang, H., Debarry, J.D., Tan, X., Li, J., Wang, X., Lee, T.H., Jin, H., Marler, B., Guo, H., Kissinger, J.C., Paterson, A.H., 2012. MCScanX: a toolkit for detection and evolutionary analysis of gene synteny and collinearity. Nucleic acids research 40, e49.

Wang, Y., Zhao, H., Liu, J., Shao, Y., Xing, M., 2019. Molecular cloning and transcriptional regulation of Indian peafowl (Pavo cristatus) IFN-α gene. Cell stress & chaperones 24, 323-332.

Watt, B., van Niel, G., Raposo, G., Marks, M.S., 2013. PMEL: a pigment cell-specific model for functional amyloid formation. Pigment cell & melanoma research 26, 300-315.

Xie, C., Mao, X., Huang, J., Ding, Y., Wu, J., Dong, S., Kong, L., Gao, G., Li, C.Y., Wei, L., 2011. KOBAS 2.0: a web server for annotation and identification of enriched pathways and diseases. Nucleic acids research 39, W316-322.

Xu, J., Ji, J., Yan, X.H., 2012. Cross-talk between AMPK and mTOR in regulating energy balance. Critical reviews in food science and nutrition 52, 373-381.

Xu, Z., Wang, H., 2007. LTR_FINDER: an efficient tool for the prediction of full-length LTR retrotransposons. Nucleic acids research 35, W265-268.

Yang, Z., 2007. PAML 4: phylogenetic analysis by maximum likelihood. Molecular biology and evolution 24, 1586-1591.

Yu, C., Zavaljevski, N., Desai, V., Reifman, J., 2011. QuartetS: a fast and accurate algorithm for large-scale orthology detection. Nucleic acids research 39, e88.

Zdobnov, E.M., Apweiler, R., 2001. InterProScan--an integration platform for the signature-recognition methods in InterPro. Bioinformatics 17, 847-848.

Zhan, X., Pan, S., Wang, J., Dixon, A., He, J., Muller, M.G., Ni, P., Hu, L., Liu, Y., Hou, H., Chen, Y., Xia, J., Luo, Q., Xu, P., Chen, Y., Liao, S., Cao, C., Gao, S., Wang, Z., Yue, Z., Li, G., Yin, Y., Fox, N.C., Wang, J., Bruford, M.W., 2013. Peregrine and saker

falcon genome sequences provide insights into evolution of a predatory lifestyle. Nat Genet 45, 563-566.

Zhang, G., Li, C., Li, Q., Li, B., Larkin, D.M., Lee, C., Storz, J.F., Antunes, A., Greenwold, M.J., Meredith, R.W., Ödeen, A., Cui, J., Zhou, Q., Xu, L., Pan, H., Wang, Z., Jin, L., Zhang, P., Hu, H., Yang, W., Hu, J., Xiao, J., Yang, Z., Liu, Y., Xie, Q., Yu, H., Lian, J., Wen, P., Zhang, F., Li, H., Zeng, Y., Xiong, Z., Liu, S., Zhou, L., Huang, Z., An, N., Wang, J., Zheng, Q., Xiong, Y., Wang, G., Wang, B., Wang, J., Fan, Y., da Fonseca, R.R., Alfaro-Núñez, A., Schubert, M., Orlando, L., Mourier, T., Howard, J.T., Ganapathy, G., Pfenning, A., Whitney, O., Rivas, M.V., Hara, E., Smith, J., Farré, M., Narayan, J., Slavov, G., Romanov, M.N., Borges, R., Machado, J.P., Khan, I., Springer, M.S., Gatesy, J., Hoffmann, F.G., Opazo, J.C., Håstad, O., Sawyer, R.H., Kim, H., Kim, K.W., Kim, H.J., Cho, S., Li, N., Huang, Y., Bruford, M.W., Zhan, X., Dixon, A., Bertelsen, M.F., Derryberry, E., Warren, W., Wilson, R.K., Li, S., Ray, D.A., Green, R.E., O'Brien, S.J., Griffin, D., Johnson, W.E., Haussler, D., Ryder, O.A., Willerslev, E., Graves, G.R., Alström, P., Fjeldså, J., Mindell, D.P., Edwards, S.V., Braun, E.L., Rahbek, C., Burt, D.W., Houde, P., Zhang, Y., Yang, H., Wang, J., Jarvis, E.D., Gilbert, M.T., Wang, J., 2014. Comparative genomics reveals insights into avian genome evolution and adaptation. Science 346, 1311-1320.

Zhang, Z., Li, J., Zhao, X.Q., Wang, J., Wong, G.K., Yu, J., 2006. KaKs_Calculator: calculating Ka and Ks through model selection and model averaging. Genomics, proteomics & bioinformatics 4, 259-263.

Zhou, T.C., Sha, T., Irwin, D.M., Zhang, Y.P., 2015. Complete mitochondrial genome of the Indian peafowl (Pavo cristatus), with phylogenetic analysis in phasianidae. Mitochondrial DNA 26, 912-913.

Figures legend

Fig. 1. Photographs of the Indian blue peafowl and white peafowl. The Indian blue peafowl and white peafowl showed in **a** and **b**, respectively.

Fig. 2. The global maps of de novo genome assembly of Indian peafowl. **a**, 55 scaffolds with the length greater than 5 Mb (scaffolds N70) of the assembled Indian blue peafowl. The perimeter of ring represented the length of scaffolds, and the light orange links in the middle circle indicated the synteny in the peafowl genome. The GC density, gene density and tandem repeat sequence density of peafowl genome were displayed in **b**, **c** and **d**, respectively. And the red and green bars in the gene density diagram represented the positive strand (+) and negative strand (-) in peafowl genome.

Fig. 3. Gene family and genome evolution among the peafowl and other 14 species. **a**, Statistics of orthologs among 15 species. "1:1:1" indicated the single-copy orthologs were shared by 15 species with one copy. "N:N:N" represented any other orthologous group (missing in one species). Specices-specific showed the specific orthologs in each species. Other orthologs were unclustered into gene families. **b**, Venn diagram of the shared orthologous gene families among the Phasianidae species (peafowl, Japanese quail, chicken and turkey). The numbers represented the unique or common gene family among the species. **c,** The phylogenetic relationship tree among 15 species was constructed by maximum likelihood with JTT model based on the single-copy orthologous sequences, human and mouse as outgroups. The divergence time of species was estimated by five calibration time from TimeTree database, including human-mouse (85~97Mya), human-zebra finch (294~323Mya), zebra finch-medium ground finch (30.4~46.8Mya), common mallard-zebra finch (93.2~104.6Mya) and saker

falcon-peregrine falcon (1.66~3.68Mya). Of them, the divergence time of human and mouse was used as a timeline at the bottom of the figure which was divided into four different periods such as tertiary, cretaceous, jurassic and triassic, and showed by different colours. In addition, the expansion and contraction of gene family in 15 species were showed at the right of species name. The red (+) and blue (-) numbers represented the expanded and contracted genes, respectively.

Fig. 4. Genome synteny and collinearity among the Indian peafowl, chicken, and turkey. a, A syntenic map of the peafowl and turkey genomes. The perimeter of ring represented the length of chromosomes labeled by different colours or scaffolds. It displayed the scaffolds eith the length greater than 5 Mb (scaffolds N70) of the assembled Indian blue peafowl, of which, the first 32 scaffolds was showed in red and other scaffolds were marked in gray. **b**, A syntenic map of the peafowl and chicken genomes. The first 35 scaffolds were showed in red, and other scaffolds were marked in gray. **c**, The distribution of Ka/Ks ratio in the genomes of peafowl, turkey, and chicken.

Fig. 5. Causal genes for white feather in blue and white peafowl. **a**, Allele frequency differences between blue and white peafowl. Scaffolds were distinguished by different colours. The candidate SNPs along with causal genes were marked by arrows, including *EDNRB* and *PMEL*. **b**, Differentially expressed genes (DEGs) related to the plumage pigmentation. The red and blue dots were used to mark the up- and down- regulated genes in blue and white peafowl, respectively. A total of 69 down-regulated genes and 52 up-regulated genes were identified, of which ten up-regulated genes were associated with the melanin deposition, and so marked out. **c**, KEGG and GO enrichment of DEGs related to the plumage pigmentation in blue and white peafowl. The darker the colour

was, the more significant the difference. The top significant pathway was enriched in the process of the melanin synthesis based on the criterion of $P < 0.05$ as significant. **d**, Allele frequencies of differentially expressed genes in blue and white peafowl. B: blue peafowl, W: white peafowl. An observation showed that the top two differential sites were located in *PMEL* and *EDNRB*. **e**, *PMEL* transcripts in the feather pulp of blue and white peafowl. B: blue peafowl, W: white peafowl. The RNA sequencing reads of *PMEL* were aligned to the assembly peafowl genome in the feather tissue of blue and white peafowl. The red arc represented the mRNA expression level of *PMEL*. Apparently, *PMEL* was normally expressed in blue peafowl but almost not expressed in white peafowl. **f**, RT- qPCR of *PMEL* transcripts in the feather pulp of blue and white peafowl. The result indicated that the relative expression of *PMEL* was significantly decreased in white peafowl compared to the mRNA expression of *PMEL* in blue peafowl ($P = 0.013$).

Tables

Table 1. Quality metrics for the peafowl genome assembly generated in the current work and for other peafowl genome assemblies published in previous studies.

Items	This study	Shubham et al. (2018)	Ruby et al. (2019)
Sequencing technology	Illumina NovaSeq 6000, PacBio RS-II, 10X Genomics, Chicoga	Illumina NextSeq 500	Illumina HiSeq, ONT
Total sequencing depth	362×	136×	236×
Total scaffolds	726	98,687	179,332
Scaffolds N50 (bp)	11,421,185	25,613	190,304
Contigs N50 (bp)	6,188,159	19,387	103,131
Longest scaffold length (bp)	38,857,732	286,113	2,488,982
Total sequence length (bp)	1,046,718,946	1,137,150,029	1,027,510,962
Total number of predicted protein-coding genes	19,465	15,970	23,153

Supplementary materials

Supplementary Figure S1. Pipeline of the draft genome assembly of Indian blue peafowl

Supplementary Figure S2. Workflow of the genome annotation of Indian blue peafowl

Supplementary Figure S3. 17-kmer frequency distribution of peafowl genome

Supplementary Figure S4. Divergence distribution of transposable element of peafowl genome by using RepeatMasker software

Supplementary Figure S5. Phylogenetic tree of 15 species constructed with IQ-tree

Supplementary Figure S6. Phylogenetic tree of 15 species constructed with RAxML

Supplementary Figure S7. *EDNRB* transcripts in the feather tissue of peafowl by IGV visualization

Supplementary Table S1. Statistics of genome assembly data of peafowl

Supplementary Table S2. Summary of de novo genome assembly of peafowl

Supplementary Table S3. Percentage of the base contents of peafowl genome

Supplementary Table S4. Statistics of paired-end reads mapping in peafowl genome

Supplementary Table S5. Number of SNPs of peafowl genome

Supplementary Table S6. Assembly assessment of completeness by using BUSCOs

Supplementary Table S7. Whole genome repetitive sequences of Indian peafowl genome predicted by homologous alignment and de novo search

Supplementary Table S8. Composition of repetitive sequences in peafowl genome

Supplementary Table S9. Prediction of protein-coding genes for peafowl genome

Supplementary Table S10. Statistics of functional annotation of protein-coding genes in the peafowl genome assembly

Supplementary Table S11. Statistics of non-coding RNAs in the assembly of peafowl

Supplementary Table S12. Functional enrichment of species-specific genes in peafowl compared with the Phasianidaes (chicken, turkey, and Japanese quail)

Supplementary Table S13. Functional categories of positively selected genes (dN/dS>1) between peafowl and chicken

Supplementary Table S14. Functional categories of positively selected genes (dN/dS>1) between peafowl and turkey

Supplementary Table S15. Functional enrichment of significantly expansive genes in peafowl

Supplementary Table S16. Functional enrichment of significantly contractive genes in peafowl

Supplementary Table S17. GO terms enrichment of positively selected genes in peafowl under branch-site model

Supplementary Table S18. KEGG pathways of positively selected genes in peafowl under branch-site model

Supplementary Table S19. Functional categories of positively selected genes in peafowl under branch model

Supplementary Table S20. Primer sequences of *PMEL* for RT-qPCR

Figure 1

Click here to access/download;Figure;Figure 1.tif

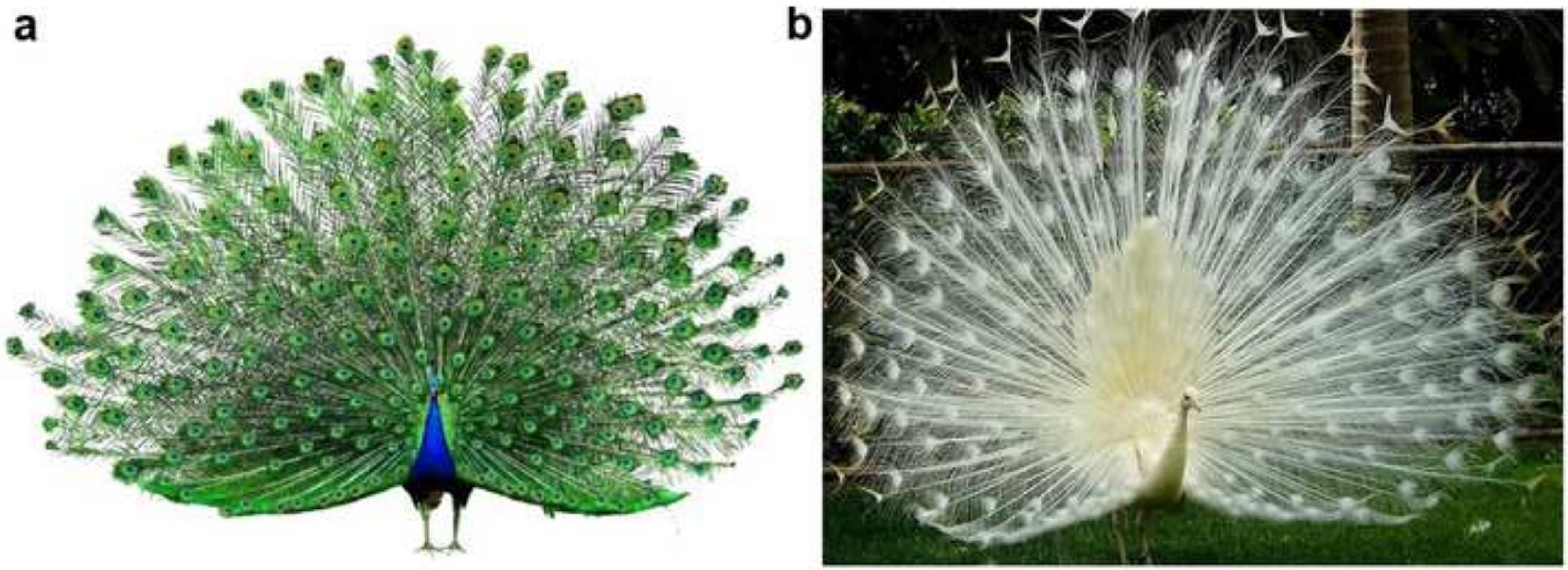

Figure 2

Click here to access/download;Figure;Figure 2.tif

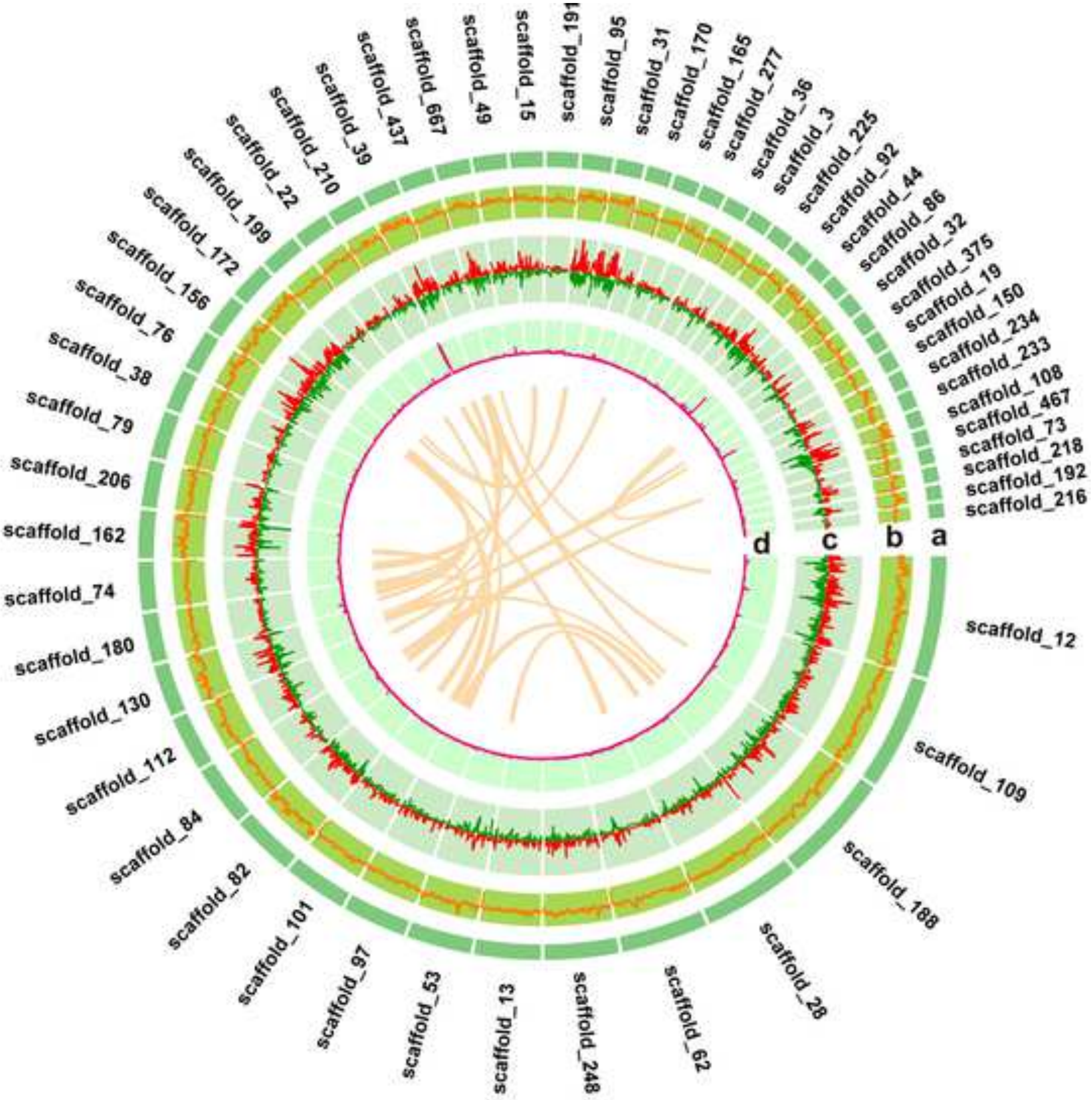

Figure 3 Click here to access/download;Figure;Figure 3.tif

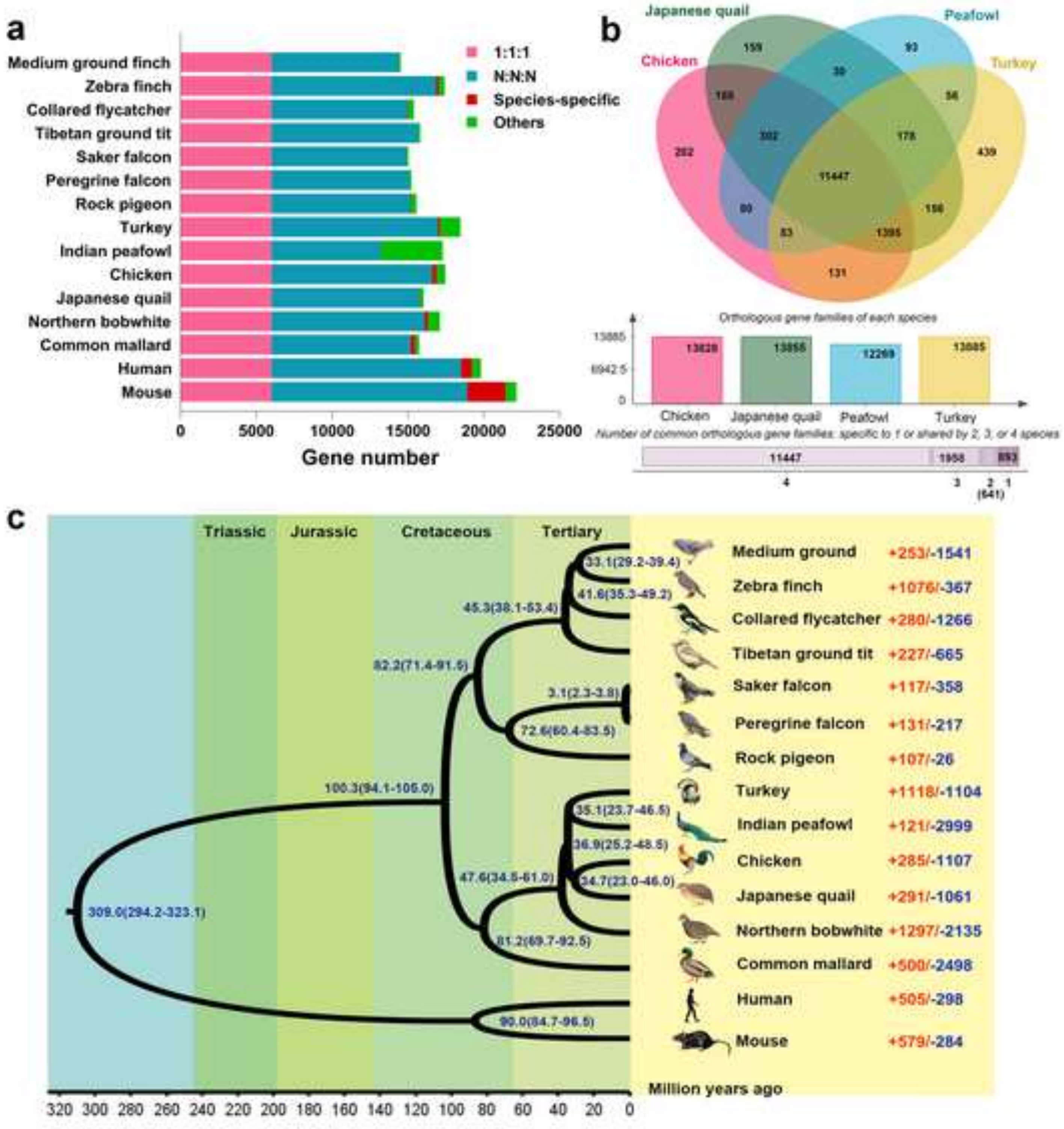

Figure 4 Click here to access/download;Figure;Figure 4.tif

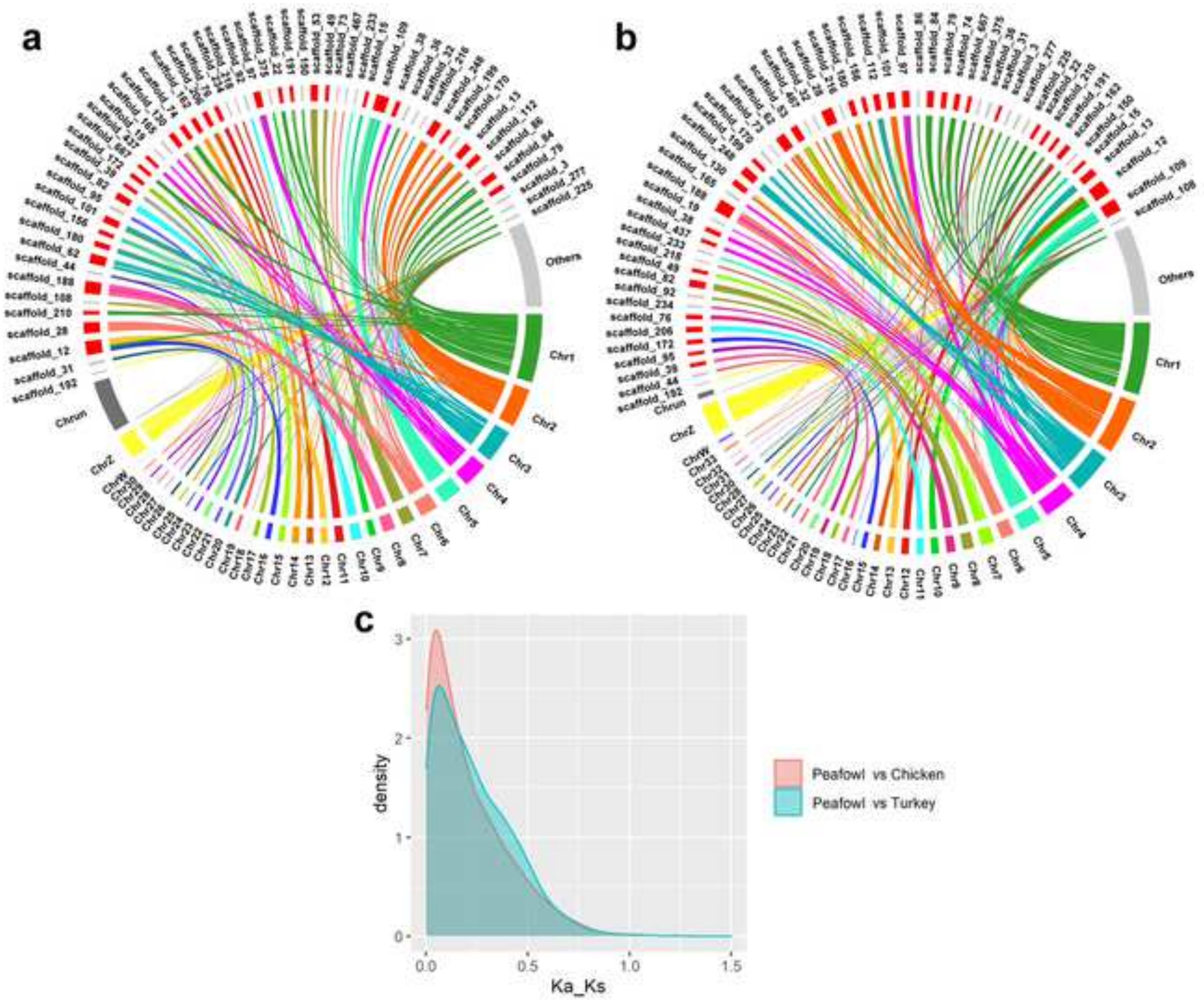

Figure 5 Click here to access/download;Figure;Figure 5.tif

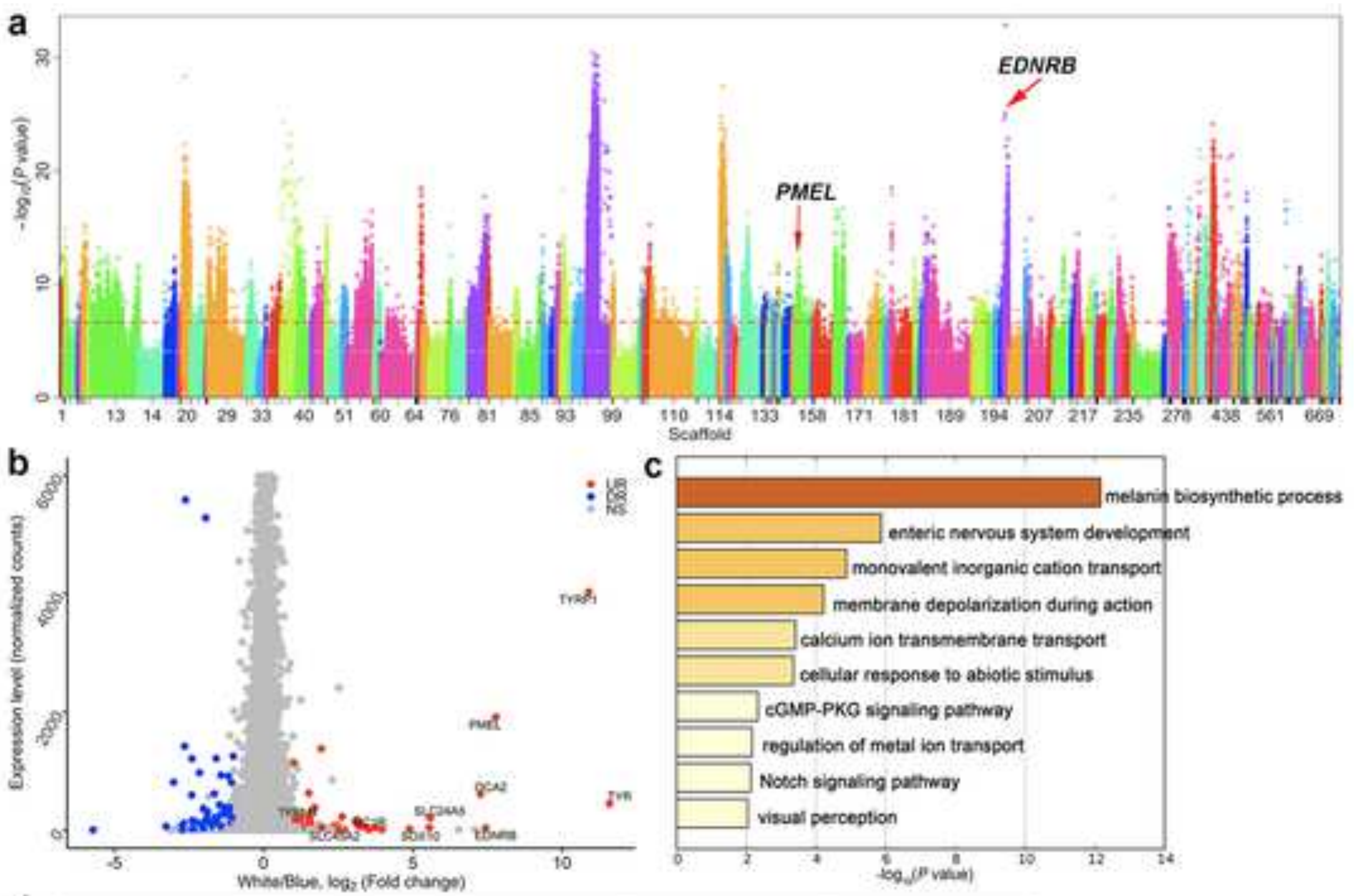

d

Scaffold	Position	Gene	Ref	Alt	Variant type	Ref		Alt	
						B_freq	W_freq	B_freq	W_freq
144	428756	PMEL	C	T	Synonymous	0.573	0.063	0.427	0.937
144	428768	PMEL	C	T	Synonymous	0.568	0.065	0.432	0.935
196	718028	EDNRB	G	A	Synonymous	0.983	0	0.017	1

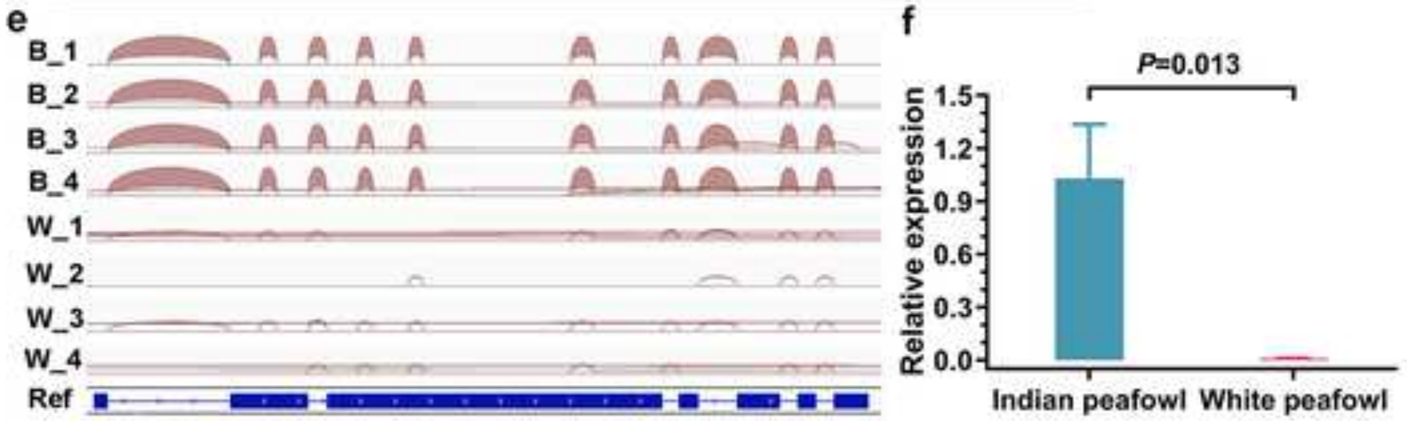

Click here to access/download
Supplementary Material
Supplementary materials.docx

Click here to access/download
Supplementary Material
Supplementary Table S12-Table S19.xlsx

supplementary figure 1 in response letter

Click here to access/download
Supplementary Material
supplementary figure 1 in response letter.jpg
