# Supplementary material for: A high-quality assembly reveals genomic characteristics, phylogenetic status, and causal genes for leucism plumage of Indian peafowl
Source: Gigascience. 2022 Apr 6;11:giac018. doi: 10.1093/gigascience/giac018 (PMC8985102; doi:10.1093/gigascience/giac018)
Supplement: giac018_Supplemental_Files [file giac018_supplemental_files.zip › Supplementary materials.docx]

**Supplementary materials**

**A high-quality assembly reveals genomic characteristics, phylogenetic status and causal genes for leucism plumage of Indian peafowl**

Shaojuan Liu^1#^, Hao Chen^3#^, Jing Ouyang^3^, Min Huang^1^, Hui Zhang^1^, Sumei Zheng^1^, Suwang Xi^2^, Hongbo Tang^3^, Yuren Gao^3^, Yanpeng Xiong^3^, Di Cheng^2^, Kaifeng Chen^2^, Bingbing Liu^1^,Wanbo Li^4^, Xueming Yan^3*^, Huirong Mao^2*^, Jun Ren ^1*^

**Table of Contents**

**Supplementary Methods1**

Genome sequence1

Illumina library preparation and sequencing2

Mate pair libraries3

Illumina sequencing4

PacBio sequencing5

10× Genomics sequencing6

**Genome annotation7**

Repeat annotation7

Structure annotation8

Homolog prediction9

*Ab initio* prediction10

RNA-seq data11

Functional annotations12

Non-coding RNA annotation13

**Supplementary Figures14**

Figure S1: Pipeline of the draft genome assembly of Indian blue peafowl14

Figure S2: Workflow of the genome annotation of Indian blue peafowl15

Figure S3: 17-kmer frequency distribution of peafowl genome16

Figure S4: Divergence distribution of transposable element of peafowl genome using RepeatMasker software17

Figure S5: Phylogenetic tree of 15 species constructed with IQ-tree18

Figure S6: Phylogenetic tree of 15 species constructed with RAxML19

Figure S7: *EDNRB* transcripts in the feather tissue of peafowl by IGV visualization20

**Supplementary Tables21**

Table S1: Statistics of genome assembly data of peafowl21

Table S2: Summary of *de novo* genome assembly of peafowl22

Table S3: Percentage of the bases contents of peafowl genome23

Table S4: Statistics of paired-end reads mapping in peafowl genome24

Table S5: Number of SNPs of peafowl genome25

Table S6: Assembly assessment of completeness using BUSCOs26

Table S7: Whole genome repeat sequences of Indian peafowl genome predicted by homologous alignment and *de novo* search27

Table S8: Composition of repetitive sequences in peafowl genome28

Table S9: Prediction of protein-coding genes for the peafowl genome29

Table S10: Statistics of functional annotation of protein-coding genes in the peafowl genome assembly30

Table S11: Statistics of non-coding RNAs in the assembly of peafowl31

Table S20: Primer sequences of *PMEL* for RT-qPCR32

**References33**

**Supplementary Methods**

**Genome sequence**

**Illumina library preparation and sequencing:**

High molecular weight DNA was extracted from a single individual and used to make a 350 bp insert fragment libraries using the Illumina TruSeq Nano method, starting with 100 ng DNA. DNA was sheared to the relevant size using a Covaris M220 Focused-ultrasonicator (Covaris). The resulting fragmented DNA was purified using Agencourt AMPure XP beads (Beckman Coulter) and checked for size distribution on an Agilent 2100 Bioanalyzer (Agilent Genomics). Following the manufacturer’s protocol, fragments were end repaired, A-tailed and adapter ligated. Enrichment was achieved with eight rounds of PCR. Libraries were checked using a Qubit Fluorometer (Invitrogen) and a high sensitively chip on the Agilent Bioanalyzer.

**Mate pair libraries**

Mate pair libraries were made using the Nextera Mate Pair Sample Preparation Kit (Illumina) using the gel plus option. Following manufacturer’s protocol 4 µg of genomic DNA was tagmented. During this step, by use of a specially formulated mate pair transposome, the genomic DNA sample is simultaneously fragmented and tagged with a biotinylated mate pair junction adapter. In later steps, this biotin adapter served to facilitate purification of mate pair fragments. The DNA was cleaned on a Zymo DNA clean column (Zymo Research). The range of fragment sizes produced was checked using a DNA 12000 Bioanalyzer chip. Subsequently, the strand displacement reaction was performed and the DNA purified with AMPure XP beads. The resulting DNA fragments were size selected using a 0.75% cassette for the BluePippin (Sage Science) using two ranges: 2 Kb, 5 Kb and 10 Kb. DNA was recovered and ligated overnight at 30°C following the manufacturer’s protocol. After incubation and heat inactivation, exonuclease was added to remove DNA which hadn’t circularised. This DNA was then sheared using the Covaris M220 and purified using Streptavidin Magnetic Beads. The samples were subsequently end repaired, A-tailed and adapter ligated according to manufacturer’s instructions and enrichment was achieved with 10 rounds of PCR. Libraries were checked for quantity and quality using a Qubit assay and a high sensitivity Bioanalyzer chip.

The Qubit and Bioanalyzer QC information was used to pool the fragment and mate pair libraries into a single pool. The quality and quantity of the pool was assessed using a Qubit assay and the Agilent Bioanalyzer and subsequently by qPCR using the Illumina Library Quantification Kit (Kapa Biosystems) on a Roche Light Cycler LC480II (Roche Molecular Systems) according to manufacturer's instructions. The template DNA was denatured according to the protocol described in the Illumina cBot user guide and loaded at 10 pM and later 12 pM concentration. At last, the library was sequenced on Illumina NovaSeq 6000 platform and 150 bp paired-end reads were generated.

**Illumina sequencing**

Total genomic DNA was extracted from peripheral blood. Two paired-end genomic sequence libraries were constructed with 350 bp insert size, and sequencing was carried out on the Illumina NovaSeq 6000 platform according to the manufacturer’s instructions. The clean data were obtained by removing reads containing adapter, reads containing ploy-N and low quality reads from raw data using megablast v2.2.26 [1] with parameters ‘-v 1 -b 1 -e 1e-5 -m 8 -a 13’; the duplication_rm.v2 was used to remove the duplicated read pairs; the low-quality reads were filtered satisfying the following conditions: (1) reads with ≥ 10% unidentified nucleotides (N), (2) remove reads with adapters, (3) reads with > 20% bases having Phred quality < 5.

**PacBio** **sequencing**

Genomic DNA was sheared by a g-TUBE device (Covaris) with 20 kb settings. Sheared DNA was purified and concentrated with AmpureXP beads (Agencourt) and further used for Single-Molecule Real Time (SMRT) bell preparation according to manufacturer’s protocol (Pacific Biosciences, 20 kb template preparation using BluePippin size selection). Size selected and isolated SMRT bell fractions were purified using AmpureXP beads (Beckman Coulter, Inc.) and finally these purified SMRT bells were used for primer-and polymerase (P6) binding according to manufacturer’s binding calculator (Pacific Biosciences). DNA-Polymerase complexes were used for Magbead binding and loaded at 0.1nM on-plate concentration spending 285 SMRT cells. Single-molecule sequencing was done on a PacBio RS-II platform with C4 chemistry and yielded data filtered polymerase read base.

**10X Genomics sequencing**

An automated micro uidic system allows the combination of the functionalized gel beads and high molecular weight DNA (HMW gDNA) together with oil to form a ‘Gel bead in emulsion (GEM)’. Each GEM contains ~10 molecules of HMW gDNA and primers with unique barcodes and P5 sequencing adapters. After PCR amplification, P7 sequencing adapters are added for Illumina sequencing.

**Genome annotation**

**Repeat annotation**

A combined strategy based on homology alignment and *de novo* search to identify the whole genome repeats were applied in our repeat annotation pipeline. Tandem Repeat was extracted using TRF [2] (http://tandem.bu.edu/trf/trf.html) by *ab initio* prediction. The homolog prediction commonly used Repbase [3] (http://www.girinst.org/repbase) database employing RepeatMasker (http://www.repeatmasker.org/) software [4] and its in-house scripts (RepeatProteinMask) with default parameters to extracted repeat regions. And *ab initio* prediction built *de novo* repetitive elements database by LTR_FINDER [5] (http://tlife.fudan.edu.cn/ltr_finder/), RepeatScout (http://www.repeatmasker.org/), RepeatModeler [6] (http://www.repeatmasker.org/RepeatModeler.html) with default parameters, then all repeat sequences with lengths > 100bp and gap ‘N’ less than 5% constituted the raw transposable element (TE) library. A custom library (a combination of Repbase and our *de novo* TE library which was processed by uclust to yield a non-redundant library) was supplied to RepeatMasker for DNA-level repeat identification.

**Structure annotation**

Structural annotation of the genome incorporates *ab initio* prediction, homologybased prediction and RNA-Seq assisted prediction, was used to annotate gene models. First, we built a non-redundant protein database of *Gallus gallus*, *Meleagris gallopavo*, *Peking duck*, *Struthio camelus*, *Nipponia nippon*, and *Eastern Zhejiang white goose*. Then the protein sequences were aligned to the genome by using TBlastN with an E-value cutoff by 1E-5 [7]. The blast hits were conjoined by solar. For each blast hit, Genewise was used to predict the exact gene structure in the corresponding genomic regions. Finally, RNA-seq data were mapped to genome using Tophat (version 2.0.8) [8]. Then cufflinks (version 2.1.1) [9] (http://cufflinks.cbcb.umd.edu/) was used to assemble transcripts to gene models. We used the transcript information to revise the gene set.

**Homolog prediction**

Sequences of homologous proteins were downloaded from Ensembl/NCBI/others. Protein sequences were aligned to the genome using TblastN (v2.2.26; E-value ≤ 1e−5) [7], and then the matching proteins were aligned to the homologous genome sequences for accurate spliced alignments with GeneWise (v2.4.1) software [10] which was used to predict gene structure contained in each protein region.

***Ab initio* prediction**

For gene predication based on *ab initio*, Augustus (v3.2.3) [11], Geneid (v1.4), Genescan (v1.0), GlimmerHMM (v3.04) [12] and SNAP [13] were used in our automated gene prediction pipeline.

**RNA-seq data**

Transcriptome reads assemblies were generated with Trinity (v2.1.1) [14] for the genome annotation. To optimize the genome annotation, the RNA-Seq reads from different tissues which were aligned to genome fasta using Hisat (v2.0.4) / TopHat (v2.0.11) [8] with default parameters to identify exons region and splice positions. The alignment results were then used as input for Stringtie (v1.3.3)/Cufflinks (v2.2.1) with default parameters for genome-based transcript assembly. The non-redundant reference gene set was generated by merging genes predicted by three methods with EvidenceModeler (EVM,v1.1.1) [15] using PASA (Program to Assemble Spliced Alignment) terminal exon support and including masked transposable elements as input into gene prediction. Individual families of interest were selected for further manual curation by relevant experts.

**Functional annotations**

Gene functions were assigned according to the best match by aligning the protein sequences to the Swiss-Prot [16] using Blastp (with a threshold of E-value ≤ 1e−5). The motifs and domains were annotated using InterProScan70 [17] (v5.31) by searching against publicly available databases, including ProDom, PRINTS, Pfam, SMRT, PANTHER and PROSITE. The Gene Ontology (GO) IDs for each gene were assigned according to the corresponding InterPro entry. We predicted the proteins function by transferring annotations from the closest BLAST hit (E-value <10-5) in the Swissprot20 database and DIAMOND (v0.8.22) / BLAST hit (E-value <10-5) hit (E-value <10-5) in the NR20 database. We also mapped gene set to a KEGG pathway and identified the best match for each gene.

**Non-coding RNA annotation**

The tRNAs were predicted using the program tRNAscan-SE [18] (http://lowelab.ucsc.edu/tRNAscan-SE/). For rRNAs are highly conserved, we choose relative species’ rRNA sequence as references, predict rRNA sequences using BlastN at E-value of 1E-10. Other ncRNAs, including miRNAs and snRNAs were identified by searching against the Rfam database [19] with default parameters using the INFERNAL software [17] (http://infernal.janelia.org/).

**Supplementary Figures：**

**
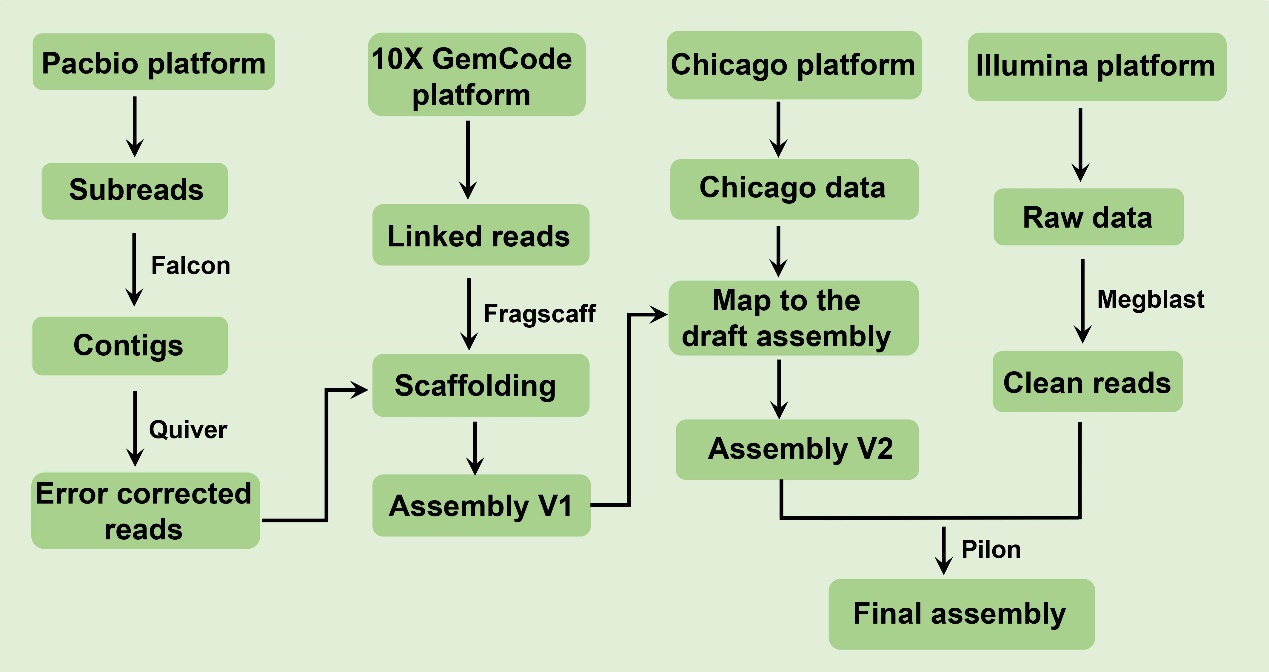
**

**Supplementary Figure S1. Pipeline of the draft genome assembly of** **Indian blue peafowl.**

**
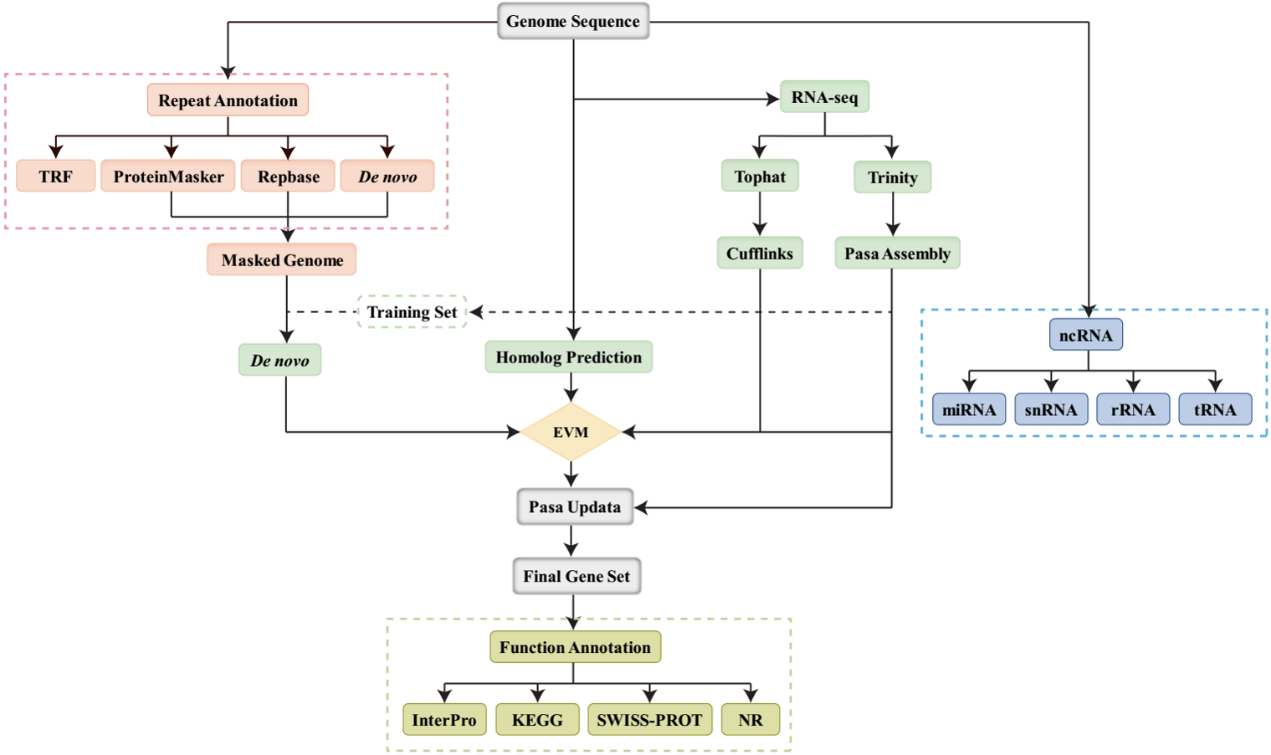
**

**Supplementary Figure S2. Workflow of the genome annotation of Indian blue peafowl.**


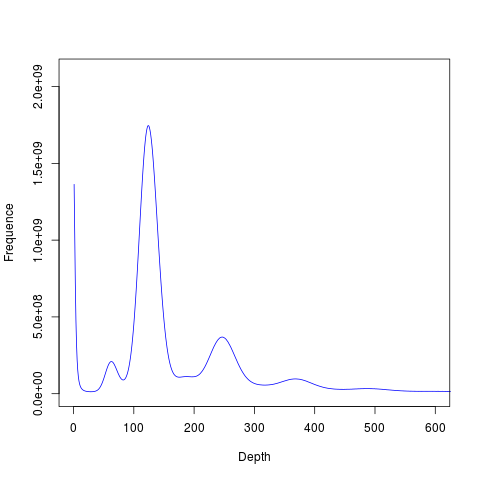


**Supplementary Figure S3. 17-kmer frequency distribution of peafowl genom**e. The genome size of peafowl was estimated to be 1.05 Gb.

**
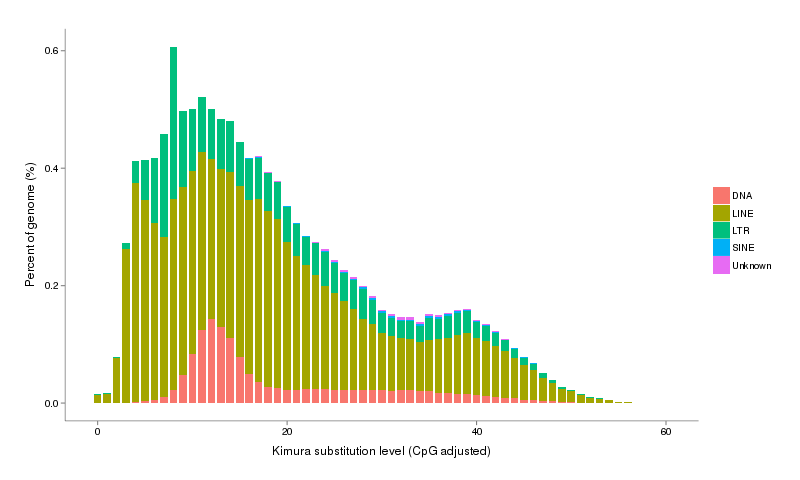
**

**Supplementary Figure S4. Divergence distribution of transposable element of peafowl genome using RepeatMasker software.**

**
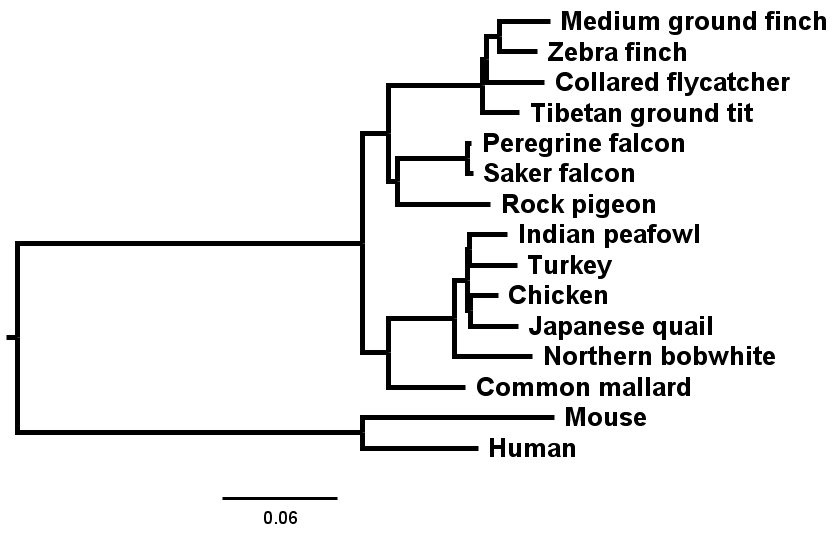
**

**Supplementary Figure S5. Phylogenetic tree of 15 species constructed with IQ-tree.** The tree was constructed by IQ-tree with 1000 bootstraps and JTT model based on the single-copy orthologs of 15 species. The result showed that peafowl was closely to turkey, and chicken was clustered with Japanese quail.

**
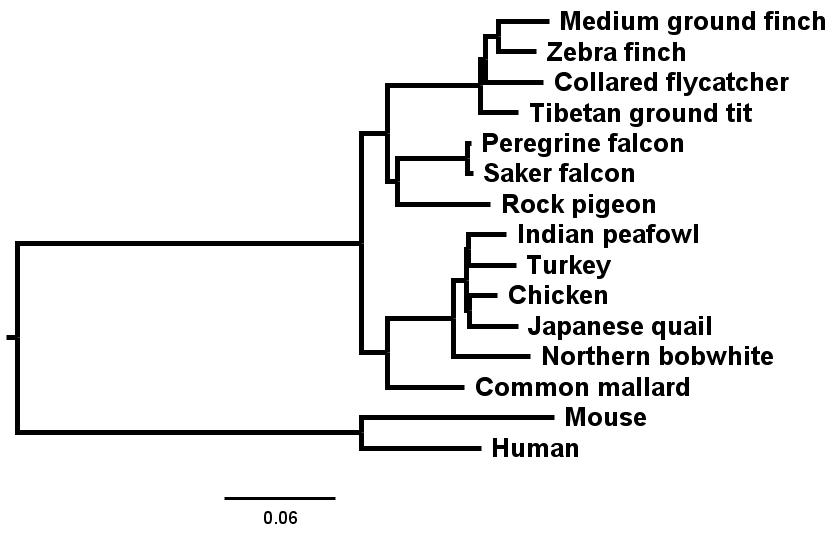
**

**Supplementary Figure S6.** **Phylogenetic tree of 15 species constructed with RAxML.** RAxML software was used to construct phylogenetic tree of 15 species with parameters “-m PROTGAMMALGX -f a” with bootstrap 1000. The position of peafowl among the *Phasianidae* birds was consistent with the result by IQ-tree that closer to turkey than chicken.

**
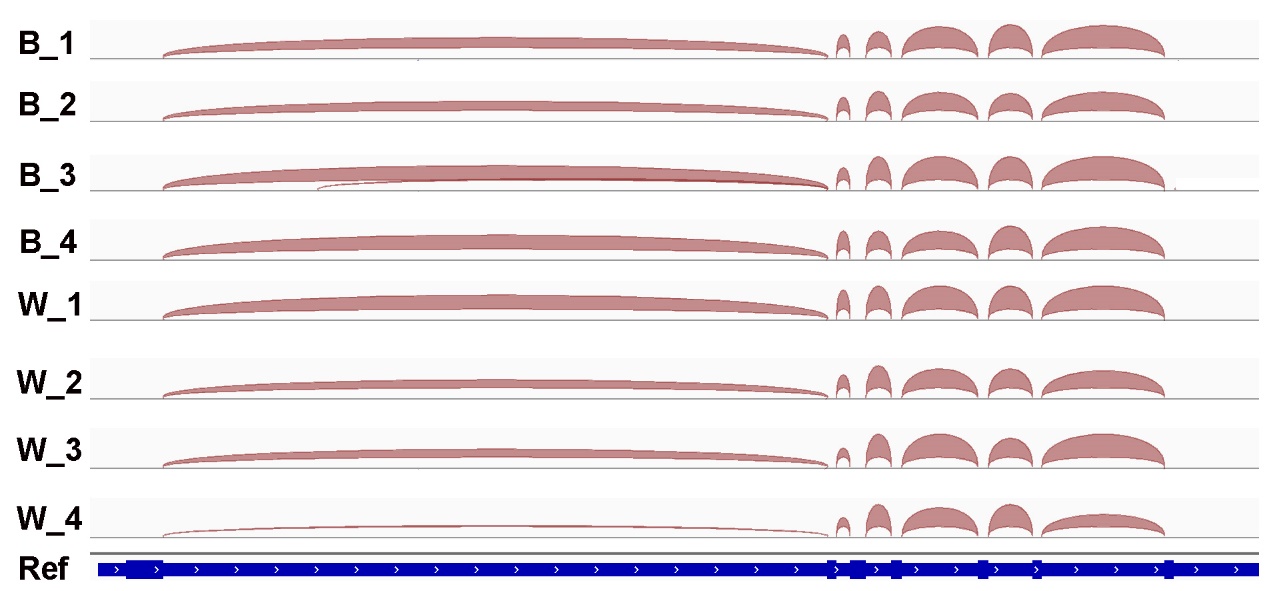
**

**Supplementary Figure S7. *EDNRB* transcripts in the feather tissue of peafowl by IGV visualization.** B: blue peafowl, W: leucism peafowl. The red arc represented the expressed abundance of genes. There was no difference in the mRNA expression of *EDNRB* between blue and leucism peafowl.

**Supplementary Tables**

**Supplementary Table S1. Statistics of genome assembly data of peafowl.**

| **Pair-end libraries** | **Insert size** | **Total data (G)** | **Read length (bp)** | **Sequence coverage (**$\boldsymbol{\times}$**)** |
| --- | --- | --- | --- | --- |
| Illumina reads | 350 | 164.03 | 150 | 154 |
| Pacbio reads | 20 | 110.74 | - | 103 |
| 10× Genomics | - | 112.57 | - | 105 |
| Total | - | 387.34 | - | 362 |

**Supplementary Table S2. Summary of *de novo* genome assembly of peafowl.**

| **Sample ID** | **Length** | | **Number** | |
| --- | --- | --- | --- | --- |
|  | **Contig(bp)** | **Scaffold(bp)** | **Contig** | **Scaffold** |
| Total | 1,043,649,651 | 1,046,718,946 | 1,198 | 726 |
| Max | 23,772,621 | 38,857,732 | - | - |
| Number>=2000 | - | - | 1,158 | 686 |
| N50 | 6,188,159 | 11,421,185 | 49 | 27 |
| N60 | 4,400,165 | 7,915,652 | 69 | 38 |
| N70 | 3,145,120 | 4,996,603 | 97 | 55 |
| N80 | 1,836,661 | 3,704,366 | 141 | 79 |
| N90 | 1,119,826 | 1,758,833 | 215 | 120 |

**Supplementary Table S3. Percentage of the bases contents of peafowl genome.**

|  | **Number (bp)** | **% of genome** |
| --- | --- | --- |
| A | 302,508,917 | 28.90 |
| T | 302,518,685 | 28.90 |
| C | 219,251,624 | 20.95 |
| G | 219,370,425 | 20.96 |
| N | 3,069,295 | 0.29 |
| Total (bp) | 1,046,718,946 | 100 |
| GC | 438,622,049 | 42.03 |

Note: GC content of the genome without N.

**Supplementary Table S4. Statistics of paired-end reads mapping in peafowl genome.**

|  |  | **Percentage** |
| --- | --- | --- |
| Reads | Mapping rate (%) | 98.05 |
| Genome | Average sequencing depth | 137.11 |
|  | Coverage (%) | 99.87 |
|  | Coverage at least 4× (%) | 99.83 |
|  | Coverage at least 10× (%) | 99.77 |
|  | Coverage at least 20× (%) | 99.68 |

**Supplementary Table S5. Number of SNPs of peafowl genome.**

| **Items** | **Number** | **Percentage（%）** |
| --- | --- | --- |
| All SNP | 1,239,760 | 0.1199 |
| Heterozygosis SNP | 1,237,532 | 0.1197 |
| Homology SNP | 2,228 | 0.0002 |

Note: All SNP: All SNPs of genome, including heterozygosis SNP and homology SNP. The percentage of homozygous SNPs showed the correct rate of genome assembly.

**Supplementary Table S6. Assembly assessment of completeness using BUSCOs.**

| BUSCO categories | Gene number | Percentage (%) |
| --- | --- | --- |
| Complete BUSCOs | 2,519 | 97.4 |
| Complete and Single-copy BUSCOs | 2,503 | 96.8 |
| Complete and duplicated BUSCOs | 16 | 0.6 |
| Fragmented BUSCOs | 44 | 1.7 |
| Missing BUSCOs | 23 | 0.9 |

**Supplementary Table S7.** **Whole genome repeat sequences of Indian peafowl genome predicted by homologous alignment and *de novo* search.**

| **Type** | **Repeat Size (bp)** | **Percent (%)** |
| --- | --- | --- |
| TRF | 13,329,377 | 1.27 |
| RepeatMasker | 147,823,121 | 14.12 |
| RepeatProteinMask | 76,804,345 | 7.34 |
| Total | 159,076,720 | 15.20 |

Note: Total is the result obtained by the above various methods, and the non-redundant result after removing the overlap between them.

**Supplementary Table S8. Composition of repetitive sequences in peafowl genome.**

| TE type | Denovo+Repbase | | TE Proteins | | Combined TEs | |
| --- | --- | --- | --- | --- | --- | --- |
|  | Length (bp) | % in Genome | Length (bp) | % in Genome | Length (bp) | % in Genome |
| DNA | 5,877,448 | 0.56 | 4,388,989 | 0.42 | 7,373,107 | 0.70 |
| LINE | 106,989,546 | 10.22 | 59,661,976 | 5.70 | 113,724,510 | 10.86 |
| SINE | 63,844 | 0.01 | 0 | 0 | 63,844 | 0.01 |
| LTR | 39,282,352 | 3.75 | 12,851,110 | 1.23 | 41,084,147 | 3.93 |
| Unknown | 2,829,364 | 0.27 | 0 | 0 | 2,829,364 | 0.27 |
| Total | 147,823,121 | 14.12 | 76,804,345 | 7.34 | 152,420,385 | 14.56 |

Note: *Denovo*+Repbase is a library predicted by RepeatModeler, RepeatScout, Piler and LTR_FINDER software combined with RepBase nucleotide library, using Uclust software to integrate according to the 80-80-80 principle, and then using RepeatMasker software to identify TEs; TE proteins are based on RepBase protein library, using RepeatProteinMask software to identify TEs; Combined TEs is the result of integrating the above two methods and removing redundancy. Unknown indicates that the repeat sequence can’t be classified by RepeatMasker.

**Supplementary Table S9. Prediction of protein-coding genes for the peafowl genome.**

| **Prediction Method** | **Gene set** | **Number** | **Average transcript length(bp)** | **Average CDS length(bp)** | **Average exons per gene** | **Average exon length(bp)** | **Average intron length(bp)** |
| --- | --- | --- | --- | --- | --- | --- | --- |
| **De novo** | **Augustus** | 15,350 | 18,679.48 | 1,492.42 | 8.58 | 173.95 | 2,267.48 |
|  | **GlimmerHMM** | 157,061 | 5,826.55 | 494.43 | 2.91 | 169.90 | 2,791.62 |
|  | **SNAP** | 44,786 | 37,825.24 | 839.36 | 6.58 | 127.62 | 6,632.06 |
|  | **Geneid** | 23,030 | 27,060.74 | 1,277.38 | 6.87 | 185.95 | 4,392.72 |
|  | **Genscan** | 32,979 | 23,773.49 | 1,399.65 | 8.37 | 167.22 | 3,035.70 |
| **Homolog** | **African ostrich** | 19,912 | 14,902.03 | 1,332.09 | 6.93 | 192.30 | 2,289.48 |
|  | **Crested ibis** | 27,835 | 11,207.12 | 1,041.79 | 5.58 | 186.67 | 2,219.06 |
|  | **Peking duck** | 21,801 | 14,382.25 | 1,274.25 | 6.72 | 189.48 | 2,289.68 |
|  | **Red junglefowl** | 21,304 | 13,813.28 | 1,228.22 | 6.62 | 185.45 | 2,238.17 |
|  | **Turkey** | 28,433 | 10,242.10 | 1,020.06 | 5.50 | 185.55 | 2,050.44 |
|  | **Zedong white goose** | 35,210 | 8,808.57 | 923.40 | 4.71 | 196.25 | 2,128.19 |
| **RNAseq** | **PASA** | 89,720 | 12,240.46 | 985.89 | 5.72 | 172.30 | 2,383.49 |
|  | **Cufflinks** | 53,708 | 29,459.41 | 4,405.71 | 9.76 | 451.38 | 2,859.82 |
| **EVM** | | 20,323 | 20,438.39 | 1,384.46 | 8.10 | 170.86 | 2,682.60 |
| **Pasa-update** | | 20,199 | 20,653.08 | 1,391.43 | 8.08 | 172.12 | 2,719.03 |
| **Final set** | | 19,465 | 21,046.08 | 1,408.58 | 8.24 | 171.01 | 2,713.61 |

**Supplementary Table S10. Statistics of functional annotation of protein-coding genes in the peafowl genome assembly.**

|  | **Number** | **Percent(%)** |
| --- | --- | --- |
| **Total** | 19,465 | - |
| **Swissprot** | 15,137 | 77.80 |
| **Nr** | 15,741 | 80.90 |
| **KEGG** | 14,137 | 72.60 |
| **InterPro** | 14,416 | 74.1 |
| **GO** | 10,347 | 53.2 |
| **Pfam** | 13,146 | 67.5 |
| **Annotated** | 15,766 | 81.00 |
| **Unannotated** | 3,699 | 19.00 |

**Supplementary Table S11. Statistics of non-coding RNAs in the assembly of peafowl.**

| **Type** | | **Copy number** | **Average length(bp)** | **Total length(bp)** | **% of genome** |
| --- | --- | --- | --- | --- | --- |
| **miRNA** | | 354 | 102.23 | 36,190 | 0.003457 |
| **tRNA** | | 308 | 76.22 | 23,477 | 0.002243 |
| **rRNA** | rRNA | 151 | 151.53 | 22,881 | 0.002186 |
|  | 18S | 23 | 235.96 | 5,427 | 0.000518 |
|  | 28S | 105 | 142.03 | 14,913 | 0.001425 |
|  | 5.8S | 0 | 0 | 0 | 0 |
|  | 5S | 23 | 110.48 | 2,541 | 0.000243 |
| **snRNA** | snRNA | 334 | 128.77 | 43,010 | 0.004109 |
|  | CD-box | 131 | 101.06 | 13,239 | 0.001265 |
|  | HACA-box | 81 | 144.16 | 11,677 | 0.001116 |
|  | splicing | 101 | 142.10 | 14,352 | 0.001371 |

**Supplementary Table S12-S19 are displayed in excel files.**

**Supplementary Table S20.** **Primer sequences of *PMEL* for RT-qPCR.**

| **Gene name** | **Forward Primer (5’ to 3’)** | **Reverse Primer (5’ to 3’)** |
| --- | --- | --- |
| *PMEL* | GGTGGTTTATCACTACCG | GTCGGTGATGCTGAACTG |
| *β-actin* | ACACGGTATTGTCACCAACT | TAACACCATCACCAGAGTCC |

Note: *β-actin* is used as an internal reference gene.

**References**

1. Chen Y, Ye W, Zhang Y, Xu Y: High speed BLASTN: an accelerated MegaBLAST search tool. Nucleic acids research 2015, 43(16):7762-7768.

2. Gant TW, Sauer UG, Zhang SD, Chorley BN, Hackermüller J, Perdichizzi S, Tollefsen KE, van Ravenzwaay B, Yauk C, Tong W et al: A generic Transcriptomics Reporting Framework (TRF) for 'omics data processing and analysis. Regulatory toxicology and pharmacology : RTP 2017, 91 Suppl 1(Suppl 1):S36-s45.

3. Jurka J, Kapitonov VV, Pavlicek A, Klonowski P, Kohany O, Walichiewicz J: Repbase Update, a database of eukaryotic repetitive elements. Cytogenetic and genome research 2005, 110(1-4):462-467.

4. Tarailo-Graovac M, Chen N: Using RepeatMasker to identify repetitive elements in genomic sequences. Current protocols in bioinformatics 2009, Chapter 4:Unit 4.10.

5. Xu Z, Wang H: LTR_FINDER: an efficient tool for the prediction of full-length LTR retrotransposons. Nucleic acids research 2007, 35(Web Server issue):W265-268.

6. Flynn JM, Hubley R, Goubert C, Rosen J, Clark AG, Feschotte C, Smit AF: RepeatModeler2 for automated genomic discovery of transposable element families. Proceedings of the National Academy of Sciences of the United States of America 2020, 117(17):9451-9457.

7. Gertz EM, Yu YK, Agarwala R, Schäffer AA, Altschul SF: Composition-based statistics and translated nucleotide searches: improving the TBLASTN module of BLAST. BMC biology 2006, 4:41.

8. Trapnell C, Pachter L, Salzberg SL: TopHat: discovering splice junctions with RNA-Seq. Bioinformatics 2009, 25(9):1105-1111.

9. Trapnell C, Roberts A, Goff L, Pertea G, Kim D, Kelley DR, Pimentel H, Salzberg SL, Rinn JL, Pachter L: Differential gene and transcript expression analysis of RNA-seq experiments with TopHat and Cufflinks. Nature protocols 2012, 7(3):562-578.

10. Birney E, Clamp M, Durbin R: GeneWise and Genomewise. Genome research 2004, 14(5):988-995.

11. Stanke M, Keller O, Gunduz I, Hayes A, Waack S, Morgenstern B: AUGUSTUS: ab initio prediction of alternative transcripts. Nucleic acids research 2006, 34(Web Server issue):W435-439.

12. Majoros WH, Pertea M, Salzberg SL: TigrScan and GlimmerHMM: two open source ab initio eukaryotic gene-finders. Bioinformatics 2004, 20(16):2878-2879.

13. Korf I: Gene finding in novel genomes. BMC bioinformatics 2004, 5:59.

14. Grabherr MG, Haas BJ, Yassour M, Levin JZ, Thompson DA, Amit I, Adiconis X, Fan L, Raychowdhury R, Zeng Q et al: Full-length transcriptome assembly from RNA-Seq data without a reference genome. Nature biotechnology 2011, 29(7):644-652.

15. Haas BJ, Salzberg SL, Zhu W, Pertea M, Allen JE, Orvis J, White O, Buell CR, Wortman JR: Automated eukaryotic gene structure annotation using EVidenceModeler and the Program to Assemble Spliced Alignments. Genome Biol 2008, 9(1):R7.

16. Bairoch A, Apweiler R: The SWISS-PROT protein sequence database and its supplement TrEMBL in 2000. Nucleic acids research 2000, 28(1):45-48.

17. Zdobnov EM, Apweiler R: InterProScan--an integration platform for the signature-recognition methods in InterPro. Bioinformatics 2001, 17(9):847-848.

18. Lowe TM, Eddy SR: tRNAscan-SE: a program for improved detection of transfer RNA genes in genomic sequence. Nucleic acids research 1997, 25(5):955-964.

19. Griffiths-Jones S, Moxon S, Marshall M, Khanna A, Eddy SR, Bateman A: Rfam: annotating non-coding RNAs in complete genomes. Nucleic acids research 2005, 33(Database issue):D121-124.
