# Supplementary material for: A high-quality assembly reveals genomic characteristics, phylogenetic status, and causal genes for leucism plumage of Indian peafowl
Source: Gigascience. 2022 Apr 6;11:giac018. doi: 10.1093/gigascience/giac018 (PMC8985102; doi:10.1093/gigascience/giac018)
Supplement: giac018_Response_to_Reviewer_Comments_Revision_1 [file giac018_response_to_reviewer_comments_revision_1.pdf]

Author's Response To Reviewer Comments

Close

Hongfang Zhang
Editor, GigaScience
Nov 15, 2021

Dear Dr. Hongfang Zhang,

Thank you for your letter and for the reviewers' comments concerning our manuscript entitled "A high-quality assembly reveals genomic characteristics, phylogenetic status and causal genes for white feather of Indian peafowl" (GIGA-D-21-00190). We greatly appreciate the opportunity to further revise this manuscript.

We sincerely appreciate the very thoughtful and constructive comments from the editor(s) and reviewers that help us to improve this manuscript. We hope our revised draft can meet your requirements and be published. Our article was polished by a professional editing company and we believe that the readability of polished article increased greatly. In addition, we carefully checked the style of revised manuscript follows the "Instruction for Authors". On the basis the reviewers' comments, we have revised the description of instruction and phylogenetic analysis. We hope that the revised manuscript now meets the standards required for publication in GigaScience.

Revised portion are marked in red in the paper. We sincerely hope that these revisions would satisfy you. The point-to-point responses to the comments are shown as follows. Please do not hesitate to contact me if you have any other questions or comments.

With best regards,
Huirong Mao
Email: maohuirong82@hotmail.com

Response to the reviewers' comments:
Reviewer # 1
This study generates a new genome assembly of the Indian peafowl and uses it to explore a variety of questions. Overall, I found the study to be well done and thorough. The authors generate a well-resolved genome using three sequencing approaches (Illumina, PacBio long-read, and 10×) and then perform a number of phylogenetic and functional/molecular evolution analyses to explore a series of questions. As someone who does not build genomes, my comments are more about the structure of the paper and the phylogenetic analysis. I have a few comments and concerns below:

1) I found this paper very hard to read in places. There are numerous grammatical errors throughout, and in places it is sometime hard to decipher the meaning of what has been written. A revision will need substantial help in the writing.
Response: We are grateful to your positive feedback on our study and greatly appreciate these valuable comments, which help us build our confidence in revising our paper comprehensively. We are deeply sorry about the writing, the revised article was polished by a professional editing company.

2) The introduction went off in a number of different tangents, making it hard to follow what the authors were actually doing. It would help to more clearly lay out the study goals (e.g. the phylogenetic component, functional component, etc.) and keep the introduction more focused. The final paragraph does this to some degree, but laying out the goals earlier would help in understanding the introduction.
Response: We are grateful to this constructive comment. We are very sorry for vaguely expressing the meaning. Here, we have reorganized the introduction and made it clear.

3) It is unclear how the other bird species were chosen for the phylogenetic analyses. There are now many more bird genomes available than used here. Rather than arbitrarily choosing a dozen, it would be better to try and fit the peafowl into the framework of previously analyses (see papers by Erich Jarvis's group). I am a bit skeptical about the relationships between the peafowl and the turkey and chicken with so few species used in the analysis. It would strengthen this section to include more species and generate a more robust phylogeny.
Response: Thanks for your advice. The other bird species we chosen were referenced from previous bird references about comparative genomic analysis (doi: 10.1093/gigascience/giy049; doi: 10.1093/gigascience/giy044; doi: 10.1093/gigascience/giy113; doi: 10.3389/fgene.2018.00392; et al.). In addition, the focus of our study is the adaptive and phenotypic evolution of peafowl by comparative genomic analysis. The fewer species we retained, the more single-copy homologous genes we obtained in the analysis, which is more accurate to estimate the evolutionary relationship of species. Moreover, the construction of phylogenetic relationship between peafowl and other birds is a critical background for subsequent comparative genomic analysis. Thus, the current results revealed that peafowl is mostly related to the turkey than chicken among the 15 selected species. We have rewritten many statements and improper expression about the phylogeny in the introduction, results and discussion referred to your suggestion.

4) Figure 5A. Why were only PMEL and ENDRB discussed in the text and highlighted here, when there are other genes of equal or higher significance showing up? I realize these two genes have been implicated in other feather color studies, but so have many of the other genes you list (e.g. TRYP1, TYR, PMEL, SLC24A5, MC1R, etc.). What do the RNAseq data show about these genes? I think you are overemphasizing two genes and leaving out some critical information about a host of other important genes. I would like to see a more balanced discussion in this section.
Response: Thanks. Plumage colours are often determined by causal genes that may have a difference in allele frequency between different plumage colour populations. On the one hand, we used resequencing data to make selective signal analysis by detecting the allele frequency difference in blue and white peafowl results suggested that only PMEL and EDNRB were involved in pigmentation. On the other hand, we used RNA-seq data to determine the DEGs between blue and white peafowl, and results indicated that 10 significantly up-regulated genes were associated with melanin deposition. Subsequently, we used resequencing data to identify the allele imbalance difference sites of 10 up-regulated genes and found that only PMEL and EDNRB had differential sites. Although there were many significant sites in the allele frequency difference, they did not cause differences in transcripts and resulted in the differential expression of related genes in the analysis of DEGs, with the exception of PMEL and EDNRB. By overlapping with the results based on resequencing and RNA-seq data, we determined that PMEL and EDNRB were candidate genes for the formation of white plumage in peafowl. Furthermore, we observed the transcripts of PMEL and EDNRB based on RNA-seq data by IGV visualization and discovered that PMEL was hardly expressed in white peafowl compared to blue peafowl; moreover, EDNRB was normally expressed in both variants. Finally, we verified the low mRNA expression of PMEL in white peafowl by RNT-qPCR; this results, suggested that PMEL was a strong candidate causative gene for the formation of white plumage.

Reviewer # 2
The authors had finished very systematic and comprehensive research. They obtained a near-chromosomal reference genome by combined several sequencing technologies and completeness of the assembled genome had reached 97.4%. In addition, it is finding a causal gene of white plumage that identifies an important gap on the genetic mechanism of the white plumage in the peafowl. The results and resources obtained from this study are valuable further comparative genomic studies in birds. The analyses are also sound and comprehensive. However, the writing of the paper is not concise and focused. The first concern is that I don't think this study is able to resolve the phylogenetic position of Pavo cristatus if whole genome data of only five species of Galliforme species were involved. Rather the purpose of phylogeny was for comparative genomics. Therefore, they should down-tune their texts about phylogeny in the introduction and discussion. Second, the apparent sister species of Pavo cristatus

is Pavo muticus. The de novo assembly of this species has been published (see Dong et al. 2021 Proc. R. Soc. B.2882021007320210073http://doi.org/10.1098/rspb.2021.0073). I wonder why author did not include the data of this species in order to increase power of their comparative genomic analysis.There are also other minor comments and suggestions for author I provide below：

Response: Thank you for your positive feedback and greatly appreciate your valuable suggestion about phylogenetic analysis in this article. (1) Firstly, concerning about the phylogenetic position of Pavo cristatus, the focus of our study is the adaptive and phenotypic evolution of peafowl by comparative genomic analysis. The construction of phylogenetic relationship between peafowl and other birds is a critical background for comparative genomic analysis. In the article, the current results only mean that peafowl is closer to turkey than chicken among the 15 selected species. Thus, we have decreased the description about phylogeny in the introduction and discussion according to your advice. Additionally, according to your advice, we supplemented and used all the available Phasianidae genome and only added two species (Bambusicola thoracica and Phasianus colchicus) to construct the phylogenetic tree. We found that the relationship of chicken and Bambusicola thoracica was closer, the genetic distance between turkey and Phasianus colchicus was closer and the phylogeny of peafowl was not changed. This new result is not much different from the current result in this study. The phylogenetic tree supplemented was shown below (supplementary figure 1 in response letter). (2) Second, about the de novo assembly of green peafowl, we also considered this species but it had not been published yet when we were working on this study. At present, although we attempt to include the data of green peafowl genome in comparative genomic analysis according to your advice, we are unable to download the annotation files of gene structure and function because the authors have not uploaded them in the NCBI. Additionally, even if we include the green peafowl in the comparative genomics, the result of phylogenetic tree should be show that the blue peafowl is close to the green peafowl. Because blue peafowl and green peafowl are different species of the same genus and have closer relationship than other Phasianidae birds. The focus of our study is not the phylogenetic position of peafowl. Thus, we think whether include the green peafowl genome data for comparative genomic analysis may be little effect on the results.

1. Line 47-57: This paragraph is less focused and authors should be more cautious about their language. Some sentences are not professional and science. For instance, "Pavo cristatus, xxxx is the king of birds".
Response: Thanks for your advice. We are very sorry for vaguely expressing the meaning. Here, we have revised this paragraph and made it clear.

2. Line 58: Author should cite some recent papers about avian genomes, for instance, Feng et al 2020 in Nature provide a dense sampling of avian genomes.
Response: Thanks a lot. We have revised it and cited some recent papers about avian genomes.

3. Line 70-82: I think this paragraph should be rephrased for two reasons: first authors did not cite some recent papers about the most updated phylogenies of Galliformes (e.g.Wang et al. PloS one, 8(5), e643120). So there arguments on the uncertainties of phylogenetic position of Pavo cristatus to some of its closely related species are not valid. Second, the focus of the present study is not the precise phylogenetic position of Pavo cristatus. Therefore, I suggest the authors to rephrase this paragraph.
Response: Thanks for your valuable suggestions. We agree that the focus of this study is not the precise phylogenetic position of Pavo cristatus but the comparative genomics. Here, we have rephrased this paragraph.

4. Line 222: How did you determine convergence of your results?
Response: We use the Tracer software to analyze the output file “mcmc.txt” and detect its efficient sampling size (ESS) value. When all of ESS value is greater than 200, we consider that the iterative operation reaches convergence. Otherwise, if it does not converge, we need to adjust the number of burnin, samplefreq or nsample and rerun the MCMCtree program until convergence.

5. Line 310-367: This part is very descriptive and needs to be condensed.
Response: Thanks for your advance. We have condensed this part.

6. Line 369: If the authors really want to reconstruct the precise phylogenetic position of Pavo cristatus,

they should involve all published de novo assemblies of birds from the family Galliformes. The current results only means that among the 15 selected species, Pavo cristatus is mostly related to the turkey.
Response: Thanks for this valuable suggestion. The focus of our study is the adaptive and phenotypic evolution of peafowl by comparative genomic analysis. The construction of phylogenetic relationship between peafowl and other birds is a critical background for comparative genomic analysis. In fact, the current results only indicate that peafowl is closer to the turkey than chicken among the 15 selected species. Thus, we have revised statements about the phylogeny of peafowl in the paper.

7. Line 377-392: This part reads like the part that should appear in Discussion.
Response: Thanks. We have revised and moved this part in Discussion.

8. Line 582-611: The structure here is not well organize and statements are not tightly linked to results.
Response: In combination with your previous suggestions, we have reorganized this part of the results and discussion and reduced the statements of the phylogeny part in discussion.

9. Line 623-627: I don't agree with these statement. So authors expected wild populations would show different patterns?
Response: Thanks for your question. We just make a hypothesis that the function of olfaction and flying of peafowl may be weakened under the condition of artificial long-term domestication. However, whether there are different patterns to wild peafowl needs further investigation.

10. Lines 679-681 : the results of detecting the SNP and Indel variation did not appear in the "Results" section.
Response: Thanks. In order to save computing resources and improve operating speed, we only detected the SNP and Indel of the scaffold 144 that PMEL was located in and did not detect the all SNP and Indel of sequencing data. Thus, we did not put the results of SNP and Indel variation in the "Results" section.

11. The language of the paper is acceptable but would definitely benefit from editing by a native speaker.
Response: Thanks for your advice. The revised article was polished by a professional editing company.

Close
