# Supplementary material for: A high-quality assembly reveals genomic characteristics, phylogenetic status, and causal genes for leucism plumage of Indian peafowl
Source: Gigascience. 2022 Apr 6;11:giac018. doi: 10.1093/gigascience/giac018 (PMC8985102; doi:10.1093/gigascience/giac018)
Supplement: giac018_Response_to_Reviewer_Comments_Revision_2 [file giac018_response_to_reviewer_comments_revision_2.pdf]

Author's Response To Reviewer Comments

Close

Hongfang Zhang
Editor, GigaScience
Dec 20, 2021

Dear Dr. Hongfang Zhang,

Thank you for your letter and for the reviewers' comments concerning our manuscript entitled "A high-quality assembly reveals genomic characteristics, phylogenetic status and causal genes for leucism plumage of Indian peafowl" (GIGA-D-21-00190R1). We greatly appreciate the second opportunity to improve this manuscript.

We sincerely appreciate the very thoughtful and constructive comments from the editor(s) and reviewers that help us to further improve this manuscript. We hope our revised draft can meet your requirements and be published. On the basis the reviewers' comments, we have carefully revised it and marked in red in the paper. We hope that the revised manuscript now meets the standards required for publication in GigaScience.

We sincerely hope that these revisions would satisfy you. The point-to-point responses to the comments are shown as follows. Please do not hesitate to contact me if you have any other questions or comments.

With best regards,
Huirong Mao
Email: maohuirong82@hotmail.com

Response to the reviewers' comments:
Reviewer # 1: I am satisfied with the changes.
Response: We sincerely appreciate the very thoughtful and constructive comments from you that help us to improve this manuscript. Meanwhile, we are glad to have your approval for the revision. Thank you very much.

Reviewer #2
I appreciate authors' efforts to revise the manuscript according reviewers' comments and suggestions. The revised version reads very well. I only have a few minor comments.

1. The authors used 'white feather' peafowl throughout the manuscript. Actually there are scientific terms about these color abnormality, for instance, leucism or albino plumage. Please define whether your samples from leucitic or albino populations. Also please change the term 'white feather' throughout the manuscript.

Response: Thanks for your carefully and strictly correction. The plumage of Indian peafowl is white but the eyes contain melanin pigmentation that caused by leucism rather than albinism. Therefore, we have revised the term ‘white feather’ to ‘leucism plumage’ throughout the manuscript.

2. For your resequencing datasets (i.e. Indian Peafowl and leucistic individuals), I suggest you calculate average heterozygosity and inbreeding index, e.g. ROH to quantify the magnitude of inbreeding. These information are help to support your arguments of inbreeding depression (Line 604-608). Further, are your candidate genes of coloration situated in ROHs?
Response: Thank you for your suggestion. Currently, we have not found relevant literature or suitable method to estimate the heterozygous rate and runs of homozygosity based on pooled sequencing data. We also attempt to conduct preliminary calculations and observe that the results are not reliable. Therefore, we have deleted the related comment of inbreeding in the article.

3. Line 107: why did you consider 'white feather' is an adaptive trait?
Response: Thanks for your question. The leucism plumage mentioned in the article should belong to phenotypic evolution, which is consistent with the description we described in the article.

4. Line 182-184; You mixed with scientific names (like Gallus callus, should be italic BTW) and captive breed name (i.e. Peking duck).
Response: Thanks for your correction. We have unified the format of species name.

5. Line 208: Should be "Tibetan"
Response: Thanks a lot. We have revised the term ‘tibetan’ to ‘Tibetan’.

6. Line 572-576: Again, your samples did not cover all species of Galliformes and yield biased results of their relationships. Please check the latest phylogenies of Galliformes using genome-wide variants (Chen et al. 2021, https://bmcecolevol.biomedcentral.com/articles/10.1186/s12862-021-01935-1). Perhaps provide a comment about the limitation of your phylogeny.
Response: Thanks for your question. In our study, we used the genomic annotation of species to construct evolutionary tree by annotated genes. We downloaded all currently available gene annotation of the Galliformes in public database, and used the single-copy genes of Galliformes to construct the most complete evolutionary tree of Galliformes by this strategy. The evolutionary tree in Chen's article was constructed by the UCE data of Galliformes and was slightly different from the results of our study, but the overall tree was consistent with each other. In addition, a large number of evolutionary trees was constructed by the same strategies and data as ours (doi: 10.1093/gigascience/giy049; doi: 10.1093/gigascience/giy044; doi: 10.1093/gigascience/giy113; doi: 10.3389/fgene.2018.00392; et al.). Therefore, we believe that our results are also reliable on the existing data.

Close
