# Supplementary material for: A high-quality assembly reveals genomic characteristics, phylogenetic status, and causal genes for leucism plumage of Indian peafowl
Source: Gigascience. 2022 Apr 6;11:giac018. doi: 10.1093/gigascience/giac018 (PMC8985102; doi:10.1093/gigascience/giac018)
Supplement: giac018_Reviewer_1_Report_Original_Submission — Dustin Rubenstein -- 8/17/2021 Reviewed [file giac018_reviewer_1_report_original_submission.pdf]

Reviewer Report

Title: A high-quality assembly reveals genomic characteristics, phylogenetic status and causal genes for leucism plumage of Indian peafowl

Version: Original Submission **Date:** 8/17/2021

Reviewer name: Dustin Rubenstein

Reviewer Comments to Author:

This study generates a new genome assembly of the Indian peafowl and uses it to explore a variety of questions. Overall, I found the study to be well done and thorough. The authors generate a well-resolved genome using three sequencing approaches (Illumina, PacBio long-read, and 10X) and then perform a number of phylogenetic and functional/molecular evolution analyses to explore a series of questions. As someone who does not build genomes, my comments are more about the structure of the paper and the phylogenetic analysis. I have a few comments and concerns below:
1) I found this paper very hard to read in places. There are numerous grammatical errors throughout, and in places it is sometime hard to decipher the meaning of what has been written. A revision will need substantial help in the writing.
2) The introduction went off in a number of different tangents, making it hard to follow what the authors were actually doing. It would help to more clearly lay out the study goals (e.g. the phylogenetic component, functional component, etc.) and keep the introduction more focused. The final paragraph does this to some degree, but laying out the goals earlier would help in understanding the introduction.
3) It is unclear how the other bird species were chosen for the phylogenetic analyses. There are now many more bird genomes available than used here. Rather than arbitrarily choosing a dozen, it would be better to try and fit the peafowl into the framework of previously analyses (see papers by Erich Jarvis's group). I am a bit skeptical about the relationships between the peafowl and the turkey and chicken with so few species used in the analysis. It would strengthen this section to include more species and generate a more robust phylogeny.
4) Figure 5A. Why were only PMEL and ENDRB discussed in the text and highlighted here, when there are other genes of equal or higher significance showing up? I realize these two genes have been implicated in other feather color studies, but so have many of the other genes you list (e.g. TRYP1, TYR, PMEL, SLC24A5, MC1R, etc.). What do the RNAseq data show about these genes? I think you are overemphasizing two genes and leaving out some critical information about a host of other important genes. I would like to see a more balanced discussion in this section.

Methods

Are the methods appropriate to the aims of the study, are they well described, and are necessary controls included? Choose an item.

Conclusions

Are the conclusions adequately supported by the data shown? Choose an item.

Reporting Standards

Does the manuscript adhere to the journal's guidelines on minimum standards of reporting? Choose an item.

Choose an item.

Statistics

Are you able to assess all statistics in the manuscript, including the appropriateness of statistical tests used? Choose an item.

Quality of Written English

Please indicate the quality of language in the manuscript: Choose an item.

Declaration of Competing Interests

Please complete a declaration of competing interests, considering the following questions:

- Have you in the past five years received reimbursements, fees, funding, or salary from an organisation that may in any way gain or lose financially from the publication of this manuscript, either now or in the future?
- Do you hold any stocks or shares in an organisation that may in any way gain or lose financially from the publication of this manuscript, either now or in the future?
- Do you hold or are you currently applying for any patents relating to the content of the manuscript?
- Have you received reimbursements, fees, funding, or salary from an organization that holds or has applied for patents relating to the content of the manuscript?
- Do you have any other financial competing interests?
- Do you have any non-financial competing interests in relation to this paper?

If you can answer no to all of the above, write 'I declare that I have no competing interests' below. If your reply is yes to any, please give details below.

I declare that I have no competing interests

I agree to the open peer review policy of the journal. I understand that my name will be included on my report to the authors and, if the manuscript is accepted for publication, my named report including any attachments I upload will be posted on the website along with the authors' responses. I agree for my report to be made available under an Open Access Creative Commons CC-BY license (http://creativecommons.org/licenses/by/4.0/). I understand that any comments which I do not wish to

be included in my named report can be included as confidential comments to the editors, which will not be published.

Choose an item.

To further support our reviewers, we have joined with Publons, where you can gain additional credit to further highlight your hard work (see: https://publons.com/journal/530/gigascience). On publication of this paper, your review will be automatically added to Publons, you can then choose whether or not to claim your Publons credit. I understand this statement.

Yes Choose an item.
