# Supplementary material for: A high-quality assembly reveals genomic characteristics, phylogenetic status, and causal genes for leucism plumage of Indian peafowl
Source: Gigascience. 2022 Apr 6;11:giac018. doi: 10.1093/gigascience/giac018 (PMC8985102; doi:10.1093/gigascience/giac018)
Supplement: giac018_Reviewer_2_Report_Original_Submission — Yang Liu, Ph.D -- 9/20/2021 Reviewed [file giac018_reviewer_2_report_original_submission.pdf]

Reviewer Report

Title: A high-quality assembly reveals genomic characteristics, phylogenetic status and causal genes for leucism plumage of Indian peafowl

Version: Original Submission **Date:** 9/20/2021

Reviewer name: Yang Liu, Ph.D

Reviewer Comments to Author:

The authors had finished very systematic and comprehensive research. They obtained a near-chromosomal reference genome by combined several sequencing technologies and completeness of the assembled genome had reached 97.4%. In addition, it is finding a causal gene of white plumage that identifies an important gap on the genetic mechanism of the white plumage in the peafowl. The results and resources obtained from this study are valuable further comparative genomic studies in birds. The analyses are also sound and comprehensive. However, the writing of the paper is not concise and focused. The first concern is that I don't think this study is able to resolve the phylogenetic position of Pavo cristatus if whole genome data of only five species of Galliforme species were involved. Rather the purpose of phylogeny was for comparative genomics. Therefore they should down-tune their texts about phylogeny in the introduction and discussion. Second, the apparent sister species of Pavo cristatus is Pavo muticus. The de novo assembly of this species has been published (see Dong et al. 2021 Proc. R. Soc. B.2882021007320210073http://doi.org/10.1098/rspb.2021.0073). I wonder why author did not include the data of this species in order to increase power of their comparative genomic analysis. There are also other minor comments and suggestions for author I provide belowï¼š

1. Line 47-57: This paragraph is less focused and authors should be more cautious about their language. Some sentences are not professional and science. For instance, "Pavo cristatus, xxxx is the king of birds".
2. Line 58: Author should cite some recent papers about avian genomes, for instance, Feng et al 2020 in Nature provide a dense sampling of avian genomes.
3. Line 70-82: I think this paragraph should be rephrased for two reasons: first authors did not cite some recent papers about the most updated phylogenies of galliformes (e.g.Wang et al. PloS one, 8(5), e643120). So there arguments on the uncertainties of phylogenetic position of Pavo cristatus to some of its closely related species are not valid. Second, the focus of the present study is not the precise phylogenetic position of Pavo cristatus. Therefore, I suggest the authors to rephrase this paragraph.
4. Line 222: How did you determine convergence of your results?
5. Line 310-367: This part is very descriptive and needs to be condensed.
6. Line 369: If the authors really want to reconstruct the precise phylogenetic position of Pavo cristatus, they should involve all published de novo assemblies of birds from the family Galliformes. The current results only means that among the 15 selected species, Pavo crostatus is mostly related to the turkey.
7. Line 377-392: This part reads like the part that should appear in Discussion.
8. Line 582-611: The structure here is not well organize and statements are not tightly linked to results.
9. Line 623-627: I don't agree with these statement. So authors expected wild populations would show different patterns?

10. Lines 679-681ï¼šthe results of detecting the SNP and Indel variation did not appear in the "Results" section.
11. The language of the paper is acceptable but would definitely benefit from editing by a native speaker.

Methods

Are the methods appropriate to the aims of the study, are they well described, and are necessary controls included? Choose an item.

Conclusions

Are the conclusions adequately supported by the data shown? Choose an item.

Reporting Standards

Does the manuscript adhere to the journal's guidelines on minimum standards of reporting? Choose an item.

Choose an item.

Statistics

Are you able to assess all statistics in the manuscript, including the appropriateness of statistical tests used? Choose an item.

Quality of Written English

Please indicate the quality of language in the manuscript: Choose an item.

Declaration of Competing Interests

Please complete a declaration of competing interests, considering the following questions:

- Have you in the past five years received reimbursements, fees, funding, or salary from an organisation that may in any way gain or lose financially from the publication of this manuscript, either now or in the future?
- Do you hold any stocks or shares in an organisation that may in any way gain or lose financially from the publication of this manuscript, either now or in the future?
- Do you hold or are you currently applying for any patents relating to the content of the manuscript?
- Have you received reimbursements, fees, funding, or salary from an organization that holds or has applied for patents relating to the content of the manuscript?
- Do you have any other financial competing interests?

- Do you have any non-financial competing interests in relation to this paper?

If you can answer no to all of the above, write 'I declare that I have no competing interests' below. If your reply is yes to any, please give details below.

I declare that I have no competing interests.

I agree to the open peer review policy of the journal. I understand that my name will be included on my report to the authors and, if the manuscript is accepted for publication, my named report including any attachments I upload will be posted on the website along with the authors' responses. I agree for my report to be made available under an Open Access Creative Commons CC-BY license (http://creativecommons.org/licenses/by/4.0/). I understand that any comments which I do not wish to be included in my named report can be included as confidential comments to the editors, which will not be published.

Choose an item.

To further support our reviewers, we have joined with Publons, where you can gain additional credit to further highlight your hard work (see: https://publons.com/journal/530/gigascience). On publication of this paper, your review will be automatically added to Publons, you can then choose whether or not to claim your Publons credit. I understand this statement.

Yes Choose an item.
